# Choice of High-Throughput Proteomics Method Affects Data Integration with Transcriptomics and the Potential Use in Biomarker Discovery

**DOI:** 10.3390/cancers14235761

**Published:** 2022-11-23

**Authors:** Sergio Mosquim Junior, Valentina Siino, Lisa Rydén, Johan Vallon-Christersson, Fredrik Levander

**Affiliations:** 1 Department of Immunotechnology, Lund University, 223 81 Lund, Sweden; 2 Division of Surgery, Department of Clinical Sciences Lund, Lund University, 223 81 Lund, Sweden; 3 Department of Surgery and Gastroenterology, Skåne University Hospital, 214 28 Malmö, Sweden; 4 Division of Oncology, Department of Clinical Sciences Lund, Lund University, 223 81 Lund, Sweden; 5 National Bioinformatics Infrastructure Sweden (NBIS), Science for Life Laboratory, Lund University, 223 81 Lund, Sweden

**Keywords:** breast cancer, proteomics, multi-omics, classification

## Abstract

**Simple Summary:**

Omics analyses provide possibilities for molecular classification of cancers to enable personalized medicine. To allow for multi-layered molecular analysis, we developed an automated protocol for the generation of proteomics data of breast cancer tumor tissue that is subjected to parallel transcriptome analysis. We compare different data acquisition strategies for proteomics and settle on data-independent acquisition, achieving high correlation with RNA between samples. The proteomics data were further used for functional analyses and tumor classification, showing the potential of the methodology.

**Abstract:**

In recent years, several advances have been achieved in breast cancer (BC) classification and treatment. However, overdiagnosis, overtreatment, and recurrent disease are still significant causes of complication and death. Here, we present the development of a protocol aimed at parallel transcriptome and proteome analysis of BC tissue samples using mass spectrometry, via Data Dependent and Independent Acquisitions (DDA and DIA). Protein digestion was semi-automated and performed on flowthroughs after RNA extraction. Data for 116 samples were acquired in DDA and DIA modes and processed using MaxQuant, EncyclopeDIA, or DIA-NN. DIA-NN showed an increased number of identified proteins, reproducibility, and correlation with matching RNA-seq data, therefore representing the best alternative for this setup. Gene Set Enrichment Analysis pointed towards complementary information being found between transcriptomic and proteomic data. A decision tree model, designed to predict the intrinsic subtypes based on differentially abundant proteins across different conditions, selected protein groups that recapitulate important clinical features, such as estrogen receptor status, HER2 status, proliferation, and aggressiveness. Taken together, our results indicate that the proposed protocol performed well for the application. Additionally, the relevance of the selected proteins points to the possibility of using such data as a biomarker discovery tool for personalized medicine.

## 1. Introduction

Breast cancer (BC) was the leading cause of cancer incidence in 2020, representing an estimated 11.7% of all cases diagnosed in 2020. Despite this number, it ranks fifth in terms of mortality [1]. Molecular classification of BC, mostly based on microarray data, has led to the definition of the “intrinsic” subtypes, with key markers such as estrogen receptor (ER), progesterone receptor (PR), human epidermal growth factor receptor (HER2), as well as the proliferation marker Ki67. These subtypes have a relevant clinical use by providing information about prognosis and treatment response [2,3,4,5,6,7]. The mammography screening program has contributed to a reduction in the mortality rate, but it is met with challenges such as overdiagnosis and overtreatment [8]. Moreover, around 25% of patients who pass the 5-year survival mark end up succumbing to recurrent disease in the coming 15 years [9]. The late recurrence rate is even higher in patients with ER-positive disease. For these reasons, an improvement in personalized medicine is needed, whereby refinements in patient stratification would increase the identification of likely true responders, reducing overdiagnosis, overtreatment, and–consequently–recurrence.

Several advances have been achieved in the classification of BC, which is mostly done at the genomic or transcriptomic level. This includes transcriptomic analysis of large cohorts, including the TCGA [10] and the SCAN-B cohort [9] with more than 7700 transcriptomes in the latest published dataset [11,12], and the development of commercial multigene assays such as Prosigna™. However, the need for better tailoring of treatments to patients points to the direction of including more proteins for prediction. Proteomics more closely reflects the phenotype, as most cellular functions are mediated by proteins [13,14], and post-translational processing may have important effects on protein activities. In mass spectrometry-based proteomics, the most widespread approaches make use of Data Dependent Acquisition (DDA) and is often combined with fractionation to reach sufficient proteome coverage, leading to limited quantitative accuracy and reproducibility, as well as increased instrument usage [15,16,17]. Still, deep proteome coverage with high-quality quantification was achieved by Johansson, H.J., et al. (2019) [14] using fractionated samples in DDA mode. Data Independent Acquisition (DIA) has the potential to overcome the limitations of DDA as the stochastic nature of data collection is reduced. A potential drawback of this method is that it generates highly complex spectra, resulting in a computationally intensive method. Consequently, different software solutions were developed to tackle this complexity. In essence, two broad strategies have been proposed in DIA, one requiring the construction of a spectral library for peptide identification, and a library-free method [17]. The usage of DIA on unfractionated samples from cancer tissue was also demonstrated by De Marchi, T., et al. (2021) [15], with improved proteome coverage in comparison to DDA. 

Aiming to evaluate the performance of different proteomic methods and assess their potential use in biomarker discovery, we developed a protocol for parallel RNA and proteome analysis of BC tissue samples, and analyzed proteome samples belonging to a BC cohort using three label-free methods, namely DDA, library-based DIA via EncyclopeDIA [18], and library-free DIA via DIA-NN [19]. Following the comparative analysis of these methods, we then select the best performing one in terms of number of identifications, reproducibility, and correlation to already existing next generation high-throughput RNA sequencing (RNA-seq) data from the same samples. Finally, we then use it for further functional analysis, as well as classification based on proteotypes. Our results indicate that DIA-NN performed the best in terms of correlation to RNA-seq data and reproducibility. Moreover, initial functional analysis suggests that proteomics data can provide complementary information to already existing RNA-seq data, indicating that patient classification could potentially be improved, translating into more tailored treatment options, and reducing overdiagnosis, overtreatment, and associated costs.

## 2. Materials and Methods

### 2.1. Clinical Samples

First, 116 breast cancer samples from SCAN-B [9,20] were obtained in collaboration with the SCAN-B Steering Committee and the Division of Oncology and Pathology, Department of Clinical Sciences Lund, Lund University. The samples correspond to flowthrough from parallel extraction of DNA and RNA from freshly preserved breast tumor tissues. Then, an aliquot of 100 μL was prepared for each of the samples and stored at −80 °C until analysis.

RNA-seq data was acquired through the Gene Expression Omnibus (series GSE96058), corresponding to gene expression data from 30,800 genes across 3273 samples.

In the different analytical steps which follow, i.e., proteomics method comparison, protein/transcript correlation analysis, Gene Set Enrichment Analysis, and decision tree classifier, subsets of these original samples were used to match samples across experiments. All samples were accompanied by clinical data registered prospectively at time of diagnosis from the Swedish National Quality Register for Breast Cancer (NKBC), which included, for instance, information on hormone receptor and HER2 statuses, lymph node status, subtyping, and tumor grade. More detailed information can be found in Supplementary Table S1.

### 2.2. Estimation of Protein Concentration

RNA concentrations were available for all samples. Therefore, a subset of 31 samples were used for quantification using a Total Protein Kit, Micro Lowry (Sigma, St. Louis, MO, USA). The concentrations were then correlated with RNA concentrations. The protein concentration in all samples was then estimated based on the regression analysis of this subset.

### 2.3. Preparation of Samples for Tryptic Digestion

The samples were allowed to thaw at +4 °C for 20 min prior to aliquoting. Then, 20 μg of each sample was transferred to KingFisher 96 deep-well microtiter plates (Thermo Fisher Scientific, Waltham, MA, USA). The proteins were reduced and alkylated. First, 1M solutions of DTT and IAA were prepared. DTT was added to the samples to a final concentration of 10 mM. The proteins were reduced at room temperature for 45 min. IAA was added to the samples to a final concentration of 40 mM. The samples were then alkylated for 45 min at room temperature in the dark. Finally, DTT was added to a final concentration of 20 mM to quench the reaction.

### 2.4. Automated HILIC Digestion

Protein digestion was automated on a KingFisher Flex robot (ThermoFisher Scientific) in a 96-well format. The tip comb was stored in plate #1. HILIC beads (ReSyn Biosciences, Edenvale, Gauteng, South Africa), were placed in plate #2 together with equilibration buffer (15% acetonitrile, 100 mM ammonium acetate pH 4.5) in a bead/protein ratio of 10:1, giving a total volume of 300 µL. Plate #3 contained equilibration buffer (500 µL), without the addition of new beads. The samples were placed in plate #4 in binding buffer (30% acetonitrile, 200 mM ammonium acetate pH 4.5) to a final volume of 300 µL. Next, 300 µL of washing solution (95% acetonitrile) was placed on plates #5–6. Plate #7 contained 290 µL of digestion buffer (50 mM ammonium bicarbonate) with sequencing-grade modified trypsin (Promega, Madison, WI, USA) in a trypsin/protein ratio of 1:20. The protocol was carried out in one step equilibration of 1 min, followed by a 30-min protein binding step. The washes were then performed for 1 min each, and digestion took place for 4 h at 37 °C with intermittent mixing every 2 min. Protease activity was then quenched with formic acid to a final concentration of 5% (*v*/*v*), and the resulting mixture was dried on a SpeedVac Vacuum Concentrator (ThermoFisher Scientific). The samples were then resuspended in 50 µL 5% formic acid and desalted on BioPureSPN Mini C18 columns (The Nest Group, Inc., Ipswich, MA, USA). Peptides were eluted in 50 μL 50% acetonitrile, 5% formic acid, and dried. The samples were then resuspended in 14 μL 0.1% formic acid. The final concentration was estimated by performing A280 measures on a NanoDrop 2000 (ThermoFisher Scientific).

### 2.5. Liquid Chromatography-Mass Spectrometry

Peptide separation and analysis was performed on an EASY-nLC 1200 liquid chromatography system (ThermoFisher Scientific) coupled online to an electrospray ionization Q-Exactive HF-X Hybrid Quadrupole-Orbitrap mass spectrometer (ThermoFisher Scientific) for all samples. Approximately 500 ng peptide solution was injected per sample and separated using a 15 cm long fused silica capillary (75 μm* 16 cm Pico Tip Emitter, New Objective, Littleton, MA, USA) packed in house with ReproSil-Pur 1.9 μm C18 material (Dr. Maisch GmbH, Ammerbuch, Germany). The peptides were separated in 0.1% FA using a 3-min gradient from 5–10% solvent B (80% ACN, 0.1% FA), followed by a 60-min linear gradient from 10% to 25% B from 3 to 63 min, followed by a 5 min gradient to 40% B, and finally a 5-min gradient to 90% B followed by 7 min isocratic washing at 90% B at a constant flow rate of 250 nL/min.

For DDA, a top 20 method was used with MS1 target 3E6 ions, maximum injection time 50 ms, and resolution 120,000. For MS2, an isolation window of 1.2 *m*/*z* and normalized collision energy 27 was employed with target 1E5 ions, maximum IT 20 ms, and resolution 15,000.

For DIA, methods were essentially as described by Searle et al [18]. The MS1 target was 3E6 ions with a maximum injection time of 55 ms and a resolution of 30,000. DIA MS2 spectra were collected using staggered windows with a loop count of 75, 8 *m*/*z* isolation windows and normalized collision energy 27 with 20 ms maximum injection time at 15000 resolution. For acquisition of a spectral library, a sample pool derived from 30 samples belonging to different subtypes was used (Supplementary Table S1). Then, 4 *m*/*z* isolation windows were used in separate acquisitions of precursor *m*/*z* mass ranges *m*/*z* 400–500, 500–600, 600–700, 700–800, 800–900, 900–1000 as described by Searle et al.

### 2.6. DDA Data Processing

All DDA RAW files were processed using MaxQuant v1.6.14.0 and searched against level 2 of the PeptideAtlas Tiered Human Integrated Search Proteome database, version June 1, 2020, [21]. First search peptide tolerance was set to 20 ppm, main search peptide tolerance was set to 4.5 ppm. Trypsin/P was specified as enzyme, and two missed cleavages were allowed, with variable N-terminal protein acetylation and methionine oxidation considered and the carbamidomethylation of cysteines as fixed modification. The false discovery rate (FDR) was set to 1% for both peptide and protein levels. Searches were performed with a match between runs disabled or enabled as indicated in the results.

For correlation purposes, a new MaxQuant run was initialized with the DDA RAW files. The parameters were kept the same, except for the database, which corresponded to the Uniprot human FASTA file as of 25 April 2019 and the corresponding predicted Prosit spectral library. The files were downloaded at https://www.proteomicsdb.org/prosit (accessed on 20 July 2020).

### 2.7. DIA Data Processing

The DIA raw data files were converted to mzML with vendor peak-picking and demultiplexing using msconvert in Proteowizard version 3.0.21098 [22]. For generation of a chromatogram library, EncyclopeDIA (version 0.9.5) was used with the data acquired from the pool in windows. Uniprot human FASTA file as of 25 April 2019 and the corresponding predicted Prosit spectral library (dated 28 January 2020), downloaded at https://www.proteomicsdb.org/prosit (accessed on 7 July 2020) was used as input for the library generation, as described by Searle et al [23]. 

For quantitative data processing in EncyclopeDIA (version 0.9.5) or DIA-NN (version 1.8), the false discovery rate (FDR) was set to 1% for both peptide and protein levels. For EncyclopeDIA, the chromatography library from above was used and settings were: expectedPeakWidth 25.0, numberOfQuantitativePeaks 5, minNumOfQuantitativePeaks 3.

For DIA-NN, library free mode was used with the same Uniprot FASTA database as input. Precursors of charge state 1–4, peptide lengths 7–30 and peptide *m*/*z* 300–1800 were considered with maximum one missed cleavage. A maximum of one variable modification per peptide was considered. For quantification, ‘robust LC (high accuracy)’ was selected, and mass accuracy was automatically set.

### 2.8. Data Analysis

Quantitative peptide data were cyclic Loess normalized using NormalyzerDE [24]. All protein group identifications were based on peptide identifications which underwent protein rollup using the RRollup approach [25] with the R implementation deposited on GitHub (https://github.com/ComputationalProteomics/ProteinRollup, accessed on 4 June 2020), where peptides passing a 1% FDR identification threshold were grouped in protein groups with a minimum of two peptides being required for such group. Samples were matched against available clinical data to add data belonging to different gene expression phenotype and risk predictor signatures. Group-wise differential abundance comparisons were performed using LIMMA statistics in NormalyzerDE. Unsupervised hierarchical clustering was performed on normalized proteome and transcriptome data from 66 samples. Clustering was performed using the ComplexHeatmap package, version 2.10.0. The data were filtered to remove ‘NA’ values prior to being filtered to remove features with low variance and converted to z-scores. Annotation corresponding to PAM50 subtyping, ER status, HER2 status, PGR status, and lymph node status were then added to the generated figures. 

All statistical analyses were performed in RStudio, using R version 4.1.3 and the R notebook is available as Supplementary File S1.

### 2.9. Gene Set Enrichment Analysis

Gene Set Enrichment Analysis (GSEA) was performed using the GSEA() function in the R package clusterProfiler using a ranked list of differentially abundant proteins or transcripts in each of the intrinsic subtypes. The list was ranked according to log2 fold change values. The aim was to find pathways, as defined by the MsigDB Hallmark set, enriched separately across the subtypes [26]. Parameters were kept default except for the *p* value adjustment method (pAdjustMethod = “fdr”) and adjusted *p* value cutoff (pvalueCutoff = 0.25). The process was independently applied to each subtype for both datasets (proteomics and transcriptomics) and the results were then compiled into a single table.

The same list of differentially abundant proteins or transcripts was also used as input for unsupervised hierarchical clustering via the ComplexHeatmap package. The data were first filtered (adjusted *p*-value < 0.1; absolute log2FoldChange ≥ 1.5) prior to being processed according to the procedure mentioned in Section 2.8.

### 2.10. Decision Tree

A conditional reference tree algorithm was used first as a feature selection tool to select the most discriminant proteins between the intrinsic subtypes, and as a decision tree model for the classification of samples into these same subtypes. The caret R package was used for such purpose [27], with the train() function used for training of the model (model = ctree). The training control parameters were kept in default, except for the resampling method (method = “boot”) in order to use bootstrapping in the training stage.

The analysis was based on a set of 223 unique protein groups differentially abundant in different subtypes as well as in different clinicopathological characteristics. The list was generated by filtering the protein groups across the different comparisons (adjusted *p*-value < 0.01; absolute log2FoldChange ≥ 2). The samples were split into a training and a test set. The training set corresponded to 70% of the data in each subtype (60 samples), i.e., data from 70% of samples in each subtype, used to train and generate the model. The test set, on the other hand, contained the remaining 30% of samples (24 samples) and was used to assess the performance of the model on samples which had never been used before. Normalized intensity values from these 223 protein groups and their corresponding transcripts were further used to generate a dataset used for unsupervised hierarchical clustering via the ComplexHeatmap R package.

## 3. Results

### 3.1. Protocol Development and Generation of Protein Datasets

In the present study, we aimed at generating a high-throughput method which allows for parallel RNA and protein measurement. As a robust RNA analysis protocol had been developed in the SCAN-B project, we developed a semi-automated protocol starting from the protein flowthroughs and evaluated the protocol in a total of 116 BC tissue samples from the SCAN-B cohort. The protocol developed provides automated sample clean-up and protein digestion, and C18 clean-up can be further automated.

As LC-MS/MS data acquisition strategies and data processing have evolved rapidly over last few years, we acquired parallel data using several methods and evaluated the results together with the available transcriptomics data for the same samples. The setup provides a unique possibility to correlate using the same bulk samples at the RNA and protein levels. 

When using DDA, a total of 6826 and 6445 protein groups were identified, with and without Matching Between Runs, respectively; for EncyclopeDIA, 5066; and for DIA-NN, 9902.

### 3.2. Performance Evaluation of Different LC-MS/MS Methods

To evaluate the performance of different label-free LC-MS/MS methods, we utilized three parameters. First, we compared the total number of identifications in each experiment (Table 1), as this number represents an indirect measure of proteome coverage and depth. By comparing these numbers across different acquisition methods, DIA-NN clearly stood out, reporting a total of 9902 protein groups. This represented an increase between 30% and 50% compared to DDA and EncyclopeDIA, respectively. This result points to increased proteome coverage and depth for DIA-NN, which is in accordance with previous reports [15].

Secondly, we looked at the number of proteins identified across all samples in the dataset, as an indication of reproducibility, with a higher number of common features being related to increased reproducibility. Table 1 shows that, again, the best performing method is DIA-NN, with a total of 3205 protein groups identified across all samples in the dataset. These numbers are followed by EncyclopeDIA (1920), DDA with match between runs (1820), and DDA without match between runs (817). Even though matching between runs seems to be a beneficial feature for DDA–more than doubling the number of shared features–it was still outperformed by DIA-NN (which also used match-between runs).

Thirdly, as high correlations for most transcript-protein pairs between different samples can be expected, we calculated global correlations between proteins and their corresponding transcripts using the different proteomics datasets. Both Pearson and Spearman correlations were calculated. A summary of these results can be seen in Table 2. For DDA data, matching between runs appears to have contributed to a 6% increase in terms of protein/transcripts pairs, and around 21% in terms of pairs with significant correlation passing the FDR threshold of 0.05. The median correlation value, however, remained generally unchanged. The same trend can be observed for EncyclopeDIA, although it performed marginally worse compared to DDA. On the other hand, an overall improvement was achieved in DIA-NN, where the median Spearman correlation score was 0.503. Moreover, after variance filtering at the median, i.e., removing the 50% of the protein/transcript entries with the lowest variance from each of the datasets prior to matching proteins and transcripts, the median Spearman correlation increased to 0.622 for DIA-NN. The high level of concordance between these protein data and RNA could also be visualized using unsupervised clustering (Supplementary Figure S1). These correlation results are similar to what was achieved in DDA with no matching between runs. However, given DIA-NN has roughly 80% more protein/transcript pairs passing the low variance filter, DIA-NN was chosen as the best performing method at this stage (Cyclic Loess normalized protein intensity data pertaining to DIA-NN processing can be found in Supplementary Table S2).

In summary, given that DIA-NN performed better in all three parameters, all posterior functional analyses were performed using data acquired by this method.

### 3.3. Gene Set Enrichment Analysis of DIA-NN and RNA-Seq Data

**Figure 1:**
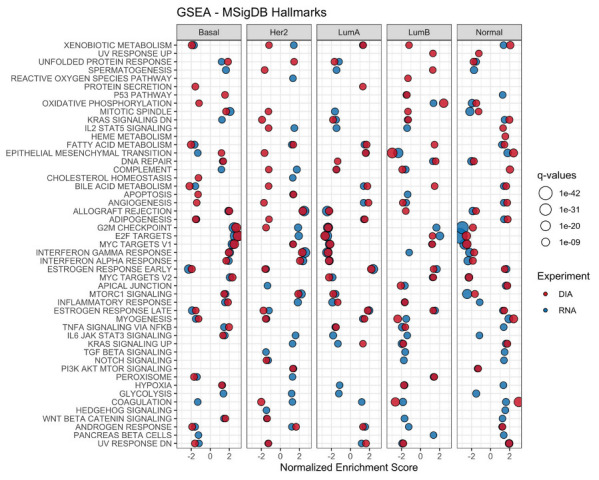
Gene Set Enrichment Analysis of transcriptomics and proteomics data in the context of the intrinsic subtypes. The analyses were performed on the different data types (RNA-seq and DIA) for each individual subtype using the Hallmark gene set. Differentially abundant proteins and transcripts for each subtype were used. Significant pathways in each experiment are shown in full color. Positive values indicate upregulated pathways, while negative values define downregulated pathways.

Since we had clinically annotated proteomics and transcriptomics data from the same samples, we investigated the possibility of these different data contributing complementary functional information. To achieve this goal, we first identified proteins and transcripts which were differentially expressed in each of the different subtypes (Luminal A, Luminal B, Basal-like, HER2-enriched, and Normal-like). We then applied Gene Set Enrichment Analysis (GSEA) to identify pathways that would be enriched (both positively and negatively) in each subtype and dataset (Figure 1).

For the most part, the pathways showed similar trends in both datasets. For instance, in luminal tumors, characterized by being ER-positive, early and late estrogen response were significantly enriched in the positive side, which is expected. Additionally, pathways associated with proliferation (e.g., E2F targets, G2M checkpoint, MYC targets, and P53)–a well-established hallmark of cancer–are among the highest absolute enrichments [28]. The results also suggest that the different data sources may contribute complementary information, with certain pathways only being significantly enriched in the proteomics data (e.g., protein secretion, epithelial mesenchymal transition, and apical junction), mainly in Basal-like and HER2-enriched tumors. These differences between the protein and RNA data are further supported by differences seen in cluster distributions using unsupervised clustering of the RNA and protein data (Supplementary Figure S2).

### 3.4. Selection of Discriminant Proteins through a Decision Tree Classifier

To assess the potential use of proteomics data in a biomarker discovery platform, we constructed a decision tree to stratify patients in the five intrinsic subtypes based on normalized protein intensities. In the context of machine learning strategies, decision trees are part of the toolbox of supervised methods, i.e., methods that are trained based on combined input and output data in order to predict the output from the input data belonging to new samples [29]. The main objective here was to use decision trees as a classifier and feature selection tool to identify protein groups which would be the most discriminant of such subtypes, therefore being indicative of potential biomarkers.

To construct the tree, a set of differentially abundant proteins was identified. This list was created based on different group comparisons: ER+ versus ER-; grade 3 versus grade 1; lymph node positive versus lymph node negative; HER2+ versus HER2-; Basal-like versus Luminal A; Luminal A versus Luminal B; Luminal B versus HER2-enriched; Basal versus HER2-enriched; and Luminal A versus HER2-enriched. A combination of features originating from the intrinsic subtypes as well as clinical subtypes and other clinical parameters was selected in an attempt to capture variability in BC. Additionally, using only intrinsic subtypes to predict intrinsic subtypes could potentially lead to overfitting of the model, thereby reducing its generalizability and possibly the relevance of the discriminating proteins as biomarkers. From such comparisons, the resulting proteins were further filtered based on log2 Fold Change > 2 and adjusted *p*-value < 0.01. A list containing 223 proteins was generated after filtering. The original DIA-NN dataset was then filtered to only contain these proteins, and samples were split into a training and a test set. The data were split by intrinsic subtype to have representation of all the subtypes.

**Figure 2:**
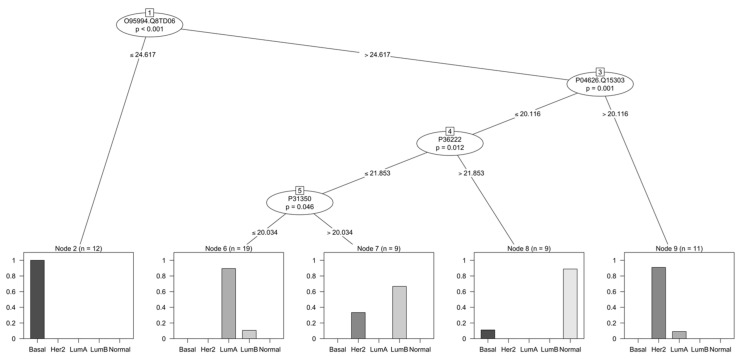
Classification of breast cancer tumors based on differentially abundant proteins. A decision tree was used for the classification of patients into five distinct subtypes. The tree was generated using a total of 223 differentially abundant proteins found across 84 breast cancer tissue samples. The bar plots indicate the number of patients belonging to each of the subtypes that were classified by the decision tree in the training dataset.

After these two sets were generated, the training set was used to train a recursive partitioning algorithm for continuous data in a conditional inference framework [27]. Bootstrapping was used as a resampling strategy during training to increase the generalizability of the model. A decision tree containing four protein groups and their thresholds was generated (Figure 2). The nodes represent the following protein groups: Anterior gradient protein 2 homolog (O95994, AGR2) and Anterior gradient protein 3 (Q8TD06, AGR3); Receptor tyrosine-protein kinase erbB-2 (P04626, HER2, ERBB2) and Receptor tyrosine-protein kinase erbB-4 (Q15303, HER4, ERBB4); Chitinase-3-like protein 1 (P36222, CHI3L1, YKL-40); and Ribonucleoside-diphosphate reductase subunit M2 (P31350, RRM2). The bar charts in Figure 2 demonstrate the sample distribution in the training as a result of the decision tree classifier. Although the separation is not perfect, the classifier was successful in selecting features of potential clinical interest in each of the subgroups. Moreover, in order to test the performance of the model in classifying new cases, a testing dataset was used. The confusion matrix resulting from such predictions can be seen in Table 3.

**Figure 3:**
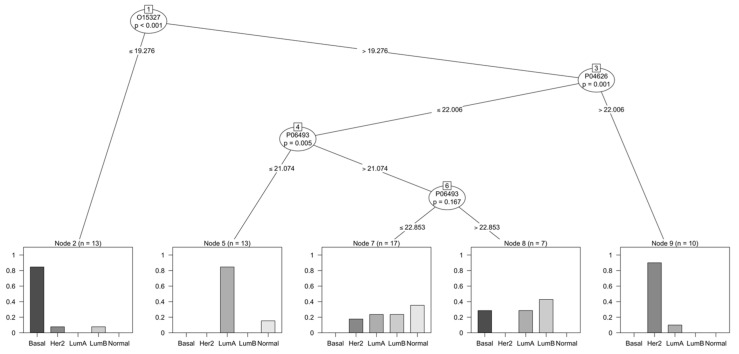
Classification of breast cancer tumors based on proteins selected by decision tree model in Bouchal, P. et al. (2019) [16]. The model was generated by selecting the three proteins described (ERBB2, CDK1 and INPP4B) and training a new model with the same datasets used previously.

As a way of comparing the performance of different models, the proteins selected by the model generated in Bouchal, P. et al. (2019) were used to create a new model to predict the intrinsic subtypes with the same dataset used to generate the model previously described. This model with the respective group distribution during training can be seen in Figure 3. The model appears to be able to classify certain subtypes with more ease, namely Basal, Luminal A, and HER-2 enriched. However, the mitotic kinase CDK1 (P06493) was not considered significant on a second node for this model, which lead to poor group separation following it.

**Figure 4:**
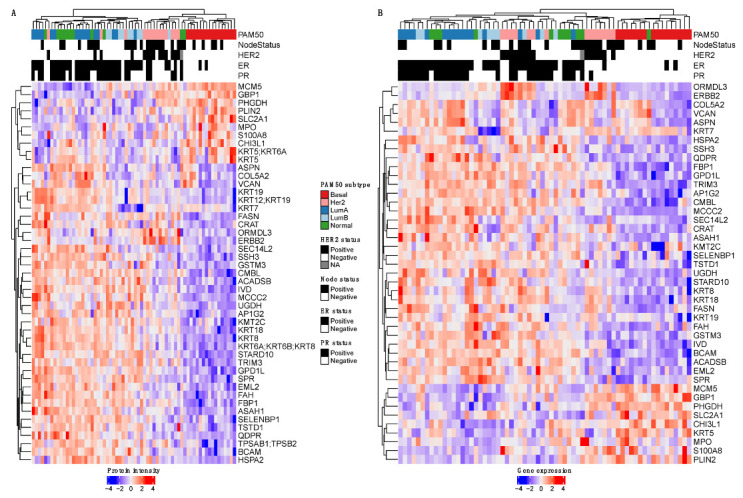
Hierarchical clustering of tumor samples (*n* = 66) in relation to PAM50 subtype and clinical features. A list of features used as input for the decision tree was used. The selected proteins were them matched to their respective genes to generate the data at the transcriptome level. (**A**) Proteome-based clustering of samples across 47 protein groups differentially expressed in a combination of subtypes and clinical features. (**B**) Transcriptome-based clustering of samples across 43 genes differentially expressed in a combination of subtypes and clinical features.

Finally, the 223 differentially abundant protein groups were used to filter the transcriptomics data, and new hierarchical clustering was performed for each omics data type across the same samples (Figure 4). A total of 47 and 43 features were used for the proteome and transcriptome data, respectively, highlighting both similarities and differences between these data.

## 4. Discussion

### 4.1. Label-Free LC-MS/MS for Cancer Classification

Several advances have been achieved in the molecular classification of BC, with the intrinsic subtypes providing relevant information about prognosis and treatment response, and even contributing to a reduction in the mortality rate. However, in the context of personalized medicine, challenges such as overtreatment, overdiagnosis, and late recurrence still point towards the need for improved patient stratification. Given the direct relationship between proteins and functions, proteomics is expected to provide useful complementary information.

In the present study, we used both DDA and DIA LC-MS/MS to acquire data from 116 BC tissue samples and compared the results with the objective to assess the impact of the choice of acquisition method on the potential use of such data in biomarker discovery. In the context of personalized medicine, the field of proteomics is moving in the direction of peptide-centric data querying strategies. Targeted approaches and DIA mitigate the issues surrounding the reproducibility and stochasticity found in DDA, but the much more complex and computationally intense DIA methods are subjected to greater variation from data processing strategies. On the other hand, biomarker discovery efforts have mostly been performed via DDA, and the often-used spectrum-centric data querying strategy makes FDR determination more robust and straightforward [17,30]. For these reasons, the selection of acquisition method and strategy is relevant.

When evaluating the different acquisition methods, our results indicate that DIA-NN outperformed EncyclopeDIA and DDA in terms of the total number of identified proteins, number of proteins found across all samples, and correlation to RNA-seq data, suggesting that DIA-NN as used here in the library-free mode offers increased proteome coverage and reproducibility. This is in agreement with what was reported by Gotti, C. et al. (2021). In their setup, different DIA methods and software tools were benchmarked. They report that DIA-NN achieves the highest number of identifications, reproducibility, and sensitivity, especially when in the FASTA mode with narrow acquisition windows [17]. It should be mentioned, however, that because EncyclopeDIA depends on the generation of a spectral library via DDA, identifications are dependent on the quality of such a library. Therefore, techniques such as fractionation would improve proteome coverage in the library, making it possible to increase the number of identifications in such setup.

Our suggested method, on the other hand, performed well in this setup. It is backed up by the higher correlation values between transcripts and their respective proteins, as well as the overall increased number of proteins identified, both overall and in terms of proteins found across all samples [14,15,16].

The good correlation between transcriptome and proteome data is further highlighted in the unsupervised hierarchical clustering of proteome and transcriptome features (Supplementary Figure S1). The same samples across different data types were used to generate the clustering, and it includes protein groups or transcripts with the highest variance in each corresponding dataset.

Despite the different number of features, the unsupervised hierarchical clustering shows good agreement, particularly in relation to PAM50 subtypes, with most Basal and HER2-enriched samples clustering separately from the Luminal and Normal-like samples. Interestingly, Normal-like samples clustered almost entirely separated from other subtypes in the proteome heatmap but not in the transcriptome one. Additionally, Luminal B samples clustered more closely to Basal and HER2-enriched in the proteome data as opposed to the transcriptome data, where they clustered more closely to Luminal A. These differences may serve as evidence for functional differences between transcriptome and proteome data.

### 4.2. Functional Differences between Proteomics and Transcriptomics

A GSEA was performed to assess the possibility of getting additional information from different data sources. Our results (Figure 1) indicate that, for the most part, proteomics and transcriptomics data showed highly similar enrichment patterns. 

Patterns associated with proliferation such as G2M checkpoint, E2F targets, and MYC targets, were among the most highly enriched in basal tumors, both based on transcriptomics and proteomics data. For basal-like tumors especially, pathways associated with MYC targets, the cell cycle checkpoint, and DNA repair seem to be enriched, which is in agreement with previous reports [31]. In luminal B samples, on the other hand, only the MYC targets pathways are positively enriched in the proteomics data, while in transcriptomics, all three pathways appear. MYC is a transcription factor able to regulate cell growth, proliferation, apoptosis, and angiogenesis. Schulze, A. et al. (2020) analyzed the correlation between MYC targets v1 and v2 pathways with proliferation. They reported that increased expression levels in these pathways were significantly correlated with high MKI67 expression, suggesting that tumors with such enrichment are linked to higher proliferation, which is a characteristic of the luminal B molecular subtype [32]. A similar trend was identified with respect to aggression, with triple-negative breast cancer (TNBC) and HER2-positive tumors, i.e., clinically more aggressive tumors, having higher scores compared to ER-positive HER2-negative subtypes. In fact, in our analysis, basal-like samples as well as HER2-positive subtypes (HER2-enriched and luminal B) showcase higher enrichment scores of MYC targets pathways compared to known HER2-negative subtypes such as luminal A and normal-like.

A similar study was conducted by Oshi, M. et al. (2020) for the G2M checkpoint pathway [33]. The regulation of this checkpoint by means of cyclin-dependent processes is a critical factor in tumorigenesis. Analogous to the findings about MYC, increased G2M pathway activity was linked to higher aggressiveness, in particular for TNBC- and HER2-positive tumors. They also found that metastasis-free survival was significantly shorter for tumors with high G2M pathway activity. As previously reported by Johansson, H.J. et al. (2019), the G2M checkpoint pathway in HER2 samples is not significantly enriched [14]. The same trend is observed for E2F targets and MYC targets. However, when comparing the transcript-protein correlation in these pathways, they appear to have relatively good Spearman correlation, suggesting that changes at the transcriptome level and the protein level would follow the same trend. Interestingly, although both significant, the direction of enrichment is different in HER2 samples for this pathway; it is positively enriched in the transcriptomics data, but negatively enriched in the proteomics data. 

According to Mertins, P. et al. (2016), agreement of the intrinsic subtypes in transcriptomics and proteomics seems to be high, even when separate tissue sections are used for each analysis. However, consensus clustering of proteome and phosphoproteome data identified three main clusters. The basal-enriched and luminal-enriched clusters showed strong overlap with basal-like and luminal samples, respectively, while a third stromal-enriched cluster had representation from all subtypes. Interestingly, HER2-enriched samples were distributed across all three clusters [31].

Enrichment in pathways associated with immune response and inflammation, e.g., T-cell, B-cell, and neutrophil signatures, was also identified at the proteome level for basal-like tumors [31]. Murthy, V. et al. (2021) investigated the association between apoptosis and immune infiltration in BC [34]. They found that tumors with high apoptosis score (based on MsigDB Hallmark gene set) were significantly enriched for the angiogenesis pathway, as well as inflammatory response and pathways associated with immune response (e.g., allograft rejection, interferon (IFN)-α response, IFN-γ response). They also found a link between high apoptosis and tumor-infiltrating lymphocytes (TILs). Our results show that basal-like and HER2-enriched tumors have the most significant positive enrichment in these pathways, while luminal A and normal-like tumors have a significant negative enrichment. In luminal B, only the inflammatory response was significantly enriched at the proteome level, suggesting that they could follow a similar pattern to luminal A tumors. However, due to their potential HER2-positive status, a mixed response could also be the cause behind the lack of significant enrichment in this subtype.

The presence of TILs in breast tumors has been associated with improved prognosis [35]. These cells are more commonly present in TNBC- and HER2-positive tumors, which is in accordance with our findings, given the significant positive enrichment in pathways associated with inflammation mainly in basal-like and HER2-enriched samples. The use of TILs as a biomarker could improve prognosis, especially for early-stage BC, and this is something that can be incorporated into routine care [35]. In TNBC- and HER2-positive tumors, TILs were shown to be associated with increased recurrence-free survival. As a predictive biomarker, increased rates of pathological complete response after neoadjuvant therapy were achieved in tumors high in TILs.

In summary, the intrinsic subtypes seem to be represented both at the transcriptome and proteome level. Our GSEA further supports this, as similar enrichments are for the most part observed in both data layers. However, as it has been observed that unsupervised clustering of proteome and phosphoproteome data results in clusters that do not fully overlap with the intrinsic subtypes, differences in enrichment can also be expected, which is observed particularly for HER2-enriched samples [31]. Our results indicate a similar trend (Supplementary Figure S2). While normal-like samples tend to cluster separately on both data types, HER2-enriched samples were distributed in multiple clusters, especially in the proteome data. This distribution appears more uniform in the transcriptome data.

### 4.3. Relevance of Proteins Found by Decision Tree

A decision tree was created to classify samples based on their LC-MS/MS data, ultimately assessing the use of such data in biomarker discovery (Figure 2). Overall, a total of four key protein groups were selected by the model as discriminant between the different subtypes, namely Anterior gradient protein 2 homolog (O95994, AGR2) and Anterior gradient protein 3 (Q8TD06, AGR3); Receptor tyrosine-protein kinase erbB-2 (P04626, HER2, ERBB2) and Receptor tyrosine-protein kinase erbB-4 (Q15303, HER4, ERBB4); Chitinase-3-like protein 1 (P36222, CHI3L1, YKL-40); and Ribonucleoside-diphosphate reductase subunit M2 (P31350, RRM2). These protein groups were all shown to be significant to the proteomics-based classifier, with *p*-value <0.05. The reason behind some of the nodes containing multiple proteins is technically from the overlap of constituent peptides analyzed using LC-MS/MS and suggests that these sum up as more discriminant than peptides unique to these specific proteins.

Anterior gradient proteins (AGR1, 2 and 3) are members of the protein disulfide isomerase family, present in the endoplasmic reticulum. Their function is associated with protein folding and protein degradation in this cell compartment, through a process known as endoplasmic reticulum-based degradation. AGR2 appears to be overexpressed in ER-positive BC, where it is often associated with poor prognosis [36,37]. It is thought to be associated with increased metastasis, cell survival, proliferation, and resistance to anti-hormone therapy. Another member of the family, AGR3, also seems to be overexpressed in BC in comparison to healthy tissue [38]. Analysis of the correlation between AGR2 and AGR3 showed that their expression is similar but not identical [38]. For instance, AGR3 seems to have better correlation to ER and grade in comparison to AGR2. Differently from AGR2, AGR3 expression appears to be linked to less aggressive tumors and a more favorable outcome [38]. Our results point in the same direction, with a lower expression of AGR2 and AGR3 separating basal-like tumors from those luminal and HER2-enriched. AGR2 is also one of the genes present in the late estrogen response pathway, and our results indicate a significant positive enrichment of this pathway in luminal and normal-like BC, while basal-like and HER2-enriched tumors have a significant negative enrichment in this pathway.

The epidermal growth factor receptor (EGFR) family of receptor tyrosine kinases is composed of four different members, EGFR (ErbB1/HER1), ErbB2 (HER2), ErbB3 (HER3), and ErbB4 (HER4). Members of this family have been extensively associated with the progression of different conditions, often related to a poor outcome [39,40]. HER2 is a well-established biomarker in BC, and its measurement is mandatory in all new cases. The overexpression of this protein leads to tumor growth by means of MAPK and PI3K/AKT signaling pathways, enhancing proliferation [41]. HER2 is classified as a predictive biomarker in the context of predicting response to anti-HER2 therapy, and in predicting overall survival and recurrence time in BC patients [41,42]. ErbB4, however, appears to be the only member of the family that is linked to the stimulation of cellular differentiation and growth inhibition. Consequently, its expression appears to be downregulated in more aggressive tumors [39]. ErbB4 seems to impair proliferation in BC by promotion of the G2/M checkpoint; it has also been demonstrated to be able to induce apoptosis [43]. Our results follow the same trend, with higher expression levels of these two proteins being associated with a HER2-enriched subtype as per the decision tree. In the pathways used for GSEA, HER2 is present as a member of the apoptosis pathway, which is significantly enriched in HER2-enriched tumors. Another pathway significantly enriched in this subtype is PI3K/AKT/MTOR signaling, which also appears to be overexpressed in response to HER2.

The next protein identified in the decision tree is CHI3L1. It has also been implicated in the promotion of inflammation (via Th2-like immune reaction), macrophage activation, tumor growth, and pro-angiogenesis [44]. Evaluation of the role of this protein in tumor promotion via cancer associated fibroblasts (CAF) suggests that CHI3L1 is upregulated in the stroma of breast tumors, as well as in fibroblasts associated with metastases, particularly in the lung [44]. A study using recombinant CHI3L1 in mice suggests that it might also be involved in promoting angiogenesis and macrophage recruitment, inducing a M2-like phenotype [44]. Macrophages are key components in the tumor microenvironment and can facilitate migration, invasion, matrix degeneration, and angiogenesis [45]. Macrophages are usually divided into M1 and M2, the latter often linked to the promotion of angiogenesis, tissue remodeling, and immunosuppression [45]. The secretion of CHI3L1 by M2 macrophages was previously reported [45]. Increased migration, adhesion, and invasiveness were associated with the presence of this protein in a study using recombinant CHI3L1 in BC cell cultures [45]. It was also evidenced that this protein could potentially be used as a diagnostic marker in BC, given that its levels in the sera of patients were significantly increased in comparison to that of healthy donors [45].

Finally, Ribonucleotide reductase is an enzyme essential to DNA replication. It is composed of four different subunits, RRM2 being one of them. It was previously shown that high expressions of this protein could serve as a prognostic indicator of poor survival in different types of cancer, including lung and colorectal cancers [46]. It has also been linked to an angiogenesis-promoting function via VEGF [46]. Immunohistochemical staining on BC tissue samples was used to demonstrate that increased RRM2 was associated with increased tumor size, positive lymph-node status, and increased relapse/metastasis [46,47]. Kaplan-Meier analysis also showed that an increase in this protein was negatively correlated with patient survival [46]. In a different study, protein expression data pointed to the significant cooccurrence of high Ki67 and high RRM2 [47]. It was also suggested that high RRM2 was more prevalent in TNBC- and HER2-enriched subtypes, though relation to the PR and ER status was not significant. Overall, this protein seems to be significantly related to increased aggressiveness and could be a potential therapeutic target in highly proliferating tumors [47]. In our GSEA, RRM2 is present in the E2F targets and MTORC1 signaling pathways. Indeed, based on these results, a highly significant positive enrichment of such pathways can be observed for basal-like and HER2-enriched samples. The decision tree results, however, suggest the use of this protein in discriminating between luminal A and luminal B/HER2-enriched subtypes.

Besides analyzing the tree in terms of the functionality of the selected protein groups, the predictability of the model can be assessed by means of the confusion matrix (Table 3). This matrix indicates that new samples belonging to Luminal A and Basal subtypes were the most correctly classified by the model; HER2-enriched and Luminal B samples had a somewhat mixed performance, while Normal-like samples were not correctly classified. The model’s accuracy was reported at 0.667 (95% CI 0.4468, 0.8437) for the test set, i.e., roughly 67% of predictions were correct.

Another method of measuring the model performance is by means of the kappa statistic. The concept was first introduced by Jacob Cohen in the context of psychological behavior as a way of measuring the degree of agreement of at least two people observing the same phenomenon [48,49]. It is a statistically robust method which takes into account classifications that may have occurred by chance [48]. The calculated Kappa for the present model was 0.5656, which indicates in general a moderate fit [50]. However, it is worth noting that the number of samples used in the present study and the class distribution of such samples are limited, both of which can contribute negatively to model construction and robustness. These results do indicate, however, that the selected protein groups have a biological role in BC and that a classification model based on proteomics data can be built and applied in biomarker discovery.

Finally, unsupervised hierarchical clustering using the decision tree data (Supplementary Figure S2) suggest an inverse trend to those using the GSEA data, i.e., the clusters based on the PAM50 subtype on the proteome data appear more uniformly distributed in comparison to those based on the transcriptome data. This further supports the biological relevance that data from proteins that are differentially abundant across different clinical parameters provides.

## 5. Conclusions

In the present study, we successfully explored and compared the use of different label-free LC-MS/MS proteomics methods in a BC cohort, establishing a new workflow for the analysis of BC tissue flowthroughs via DIA. A larger number of samples is still required to further evaluate and validate the performance of the decision tree model. However, our results suggest that proteomic technologies can provide additional information to already existing clinical subtyping. Moreover, proteins highlighted by the model show a clear link to BC, elucidating biological processes related to the disease. These findings can potentially enable a better understanding of BC biology and assist in the improvement of personalized medicine, with the development of novel biomarker signatures.

## Figures and Tables

**Table 1:** Feature Summary.

Experiment	Proteins ^1^	Common Proteins ^2^
DDA Match Between Runs	6826	1820
DDA no Match Between Runs	6445	817
DIA-NN	9902	3205
EncyclopeDIA	5066	1920

^1^ represents the total number of protein groups identified in each of the different experiments. ^2^ represents the protein groups identified across all samples in the individual runs.

**Table 2:** Correlation Summary. Across different experiments, the overall correlation is reported, as well as the correlation after removing 50% of the values with the lowest calculated variance.

Experiment	Correlation RNA-Seq	Pairs	Proteins 5% FDR * Spearman	Median Spearman Score	Proteins 5% FDR * Pearson	Median Pearson Score
DIA-NN *	Overall	8537	6113	0.503	6235	0.516
	Low variance removed	3109	2330	0.622	2355	0.644
MQ DDA MBR *	Overall	5520	3403	0.444	3400	0.452
	Low variance removed	1965	1258	0.536	1242	0.546
MQ DDA no MBR *	Overall	5211	2808	0.457	2804	0.470
	Low variance removed	1733	1072	0.600	1066	0.640
EncyclopeDIA *	Overall	4128	2878	0.436	2882	0.441
	Low variance removed	1359	1091	0.574	1118	0.591

* Acronyms: MQ: MaxQuant; MBR: Match Between Runs; FDR: False Discovery Rate; DDA: Data Dependent Acquisition; DIA: Data Independent Acquisition.

**Table 3:** Confusion Matrix. The confusion matrix was generated by applying the generated tree model on a test dataset containing samples not previously used.

	Predicted	Basal	Her2	LumA	LumB	Normal
Reference						
Basal		4	0	0	0	1
Her2		0	3	0	2	0
LumA		0	1	7	0	0
LumB		0	1	0	2	0
Normal		0	2	1	0	0

## Data Availability

The processed data presented in this study are partially available in Supplementary Tables S1 and S2. Additional data available upon request from the corresponding author.

## Supplementary Materials

The following supporting information can be downloaded at: https://www.mdpi.com/article/10.3390/cancers14235761/s1, Figure S1: Unsupervised hierarchical clustering of tumor samples (*n* = 66) in relation to PAM50 subtype and clinical features after removal of low variance features. A Proteome-based clustering of samples across 1600 protein groups with highest variance. B Transcriptome-based clustering of samples across 15,432 genes with highest variance. Figure S2: Hierarchical clustering of tumor samples (*n* = 66) in relation to PAM50 subtype and clinical features. A list of features used in GSEA was used as input after filtering (adjusted *p*-value < 0.1; absolute log2FoldChange ≥ 1.5). A Proteome-based clustering of samples across 266 protein groups differentially expressed in each subtype. B Transcriptome-based clustering of samples across 1812 genes differentially expressed in each subtype. Table S1: Summary of clinicopathological features from the samples used in the present study [11]. The file is used as reference for differential expression analyses throughout the data analysis process. Table S2: Protein abundance data from DIA-NN analysis after cyclic Loess normalization and protein rollup. File S1: R notebook of data analyses conducted for the present study.
