# Supplementary material for: Choice of High-Throughput Proteomics Method Affects Data Integration with Transcriptomics and the Potential Use in Biomarker Discovery
Source: Cancers (Basel). 2022 Nov 23;14(23):5761. doi: 10.3390/cancers14235761 (PMC9736226; doi:10.3390/cancers14235761)
Supplement: Supplementary file 1 [file cancers-14-05761-s001.zip › supplementary_files/supplementary_fileS1.html]

SCANB Data Analysis


Code 

- Show All Code
- Hide All Code
- Download Rmd

# SCANB Data Analysis

#### Sergio Mosquim Junior

#### 2022-09-26


# 1 Required Packages


```
# Pre-processing
library(tidyverse)
library(NormalyzerDE)
library(biomaRt)

# Correlations
library(gdata)

# Gene Set Enrichment Analysis
library(clusterProfiler)
library(msigdbr)
library(org.Hs.eg.db)
library(ggplot2)

# Decision Trees
library(caret)

# Unsupervised Hierarchical Clustering
library(ComplexHeatmap)
library(grid)
library(ggplotify)
library(cowplot)
```

# 2 Generate Design Files used in data analysis

## 2.1 DDA and DIA pre-processing

The overall\_design file is the Supplementary Table S1 from
“Vallon-Christersson, J.; Hakkinen, J.; Hegardt, C.; Saal, L.H.;
Larsson, C.; Ehinger, A.; Lindman, H.; Olofsson, H.; Sjoblom, T.;
Warnberg, F.; et al. Cross comparison and prognostic assessment of
breast cancer multigene signatures in a large population-based
contemporary clinical series. Scientific reports 2019, 9, 12184, doi:10.1038/s41598-019-48570-x.” The file was further
filtered to only include the samples in the present study.


```
baseDesign <- read_tsv('overall_design.tsv')
# DIA
designDIA <- clinicalAll %>% 
  dplyr::select(-DDA) %>% 
  filter(!is.na(DIA))
write_tsv(x = designDIA,file = 'DIA/DesignFilesDIA/design_aim2_clinical.tsv')
# DDA
designDDA <- clinicalAll %>% 
  dplyr::select(-DIA) %>% 
  filter(!is.na(DDA))
write_tsv(x = designDDA,file = 'DDA/DesignFilesDDA/design_aim2_clinical.tsv')
```

## 2.2 RNA-seq pre-processing

The “scanb\_gene\_design.txt” file was created by downloading the
series matrix file available on Gene Expression Omnibus ID GSE96058 and
further removing the header and transposing it, so that the variables
would align with their titles


```
baseDesign <- read_tsv('overall_design.tsv')
designRNA <- read_tsv(file = 'RNA-seq/scanb_gene_design.txt')
designRNA <- map_dfr(.x = designRNA[,10],.f = ~str_sub(string = .x,37,43)) %>% 
  unname(.) %>% 
  unlist(.) %>%
  mutate(designRNA,Specimen=.,.after = '!Sample_title')
RNAColnames <- str_remove(string = colnames(designRNA),pattern = '!')
colnames(designRNA) <- RNAColnames
designRNA <- designRNA %>% 
  filter(str_detect(string = Sample_title,pattern = 'repl',negate = TRUE)) %>% 
  dplyr::select(Sample_title,Specimen)

designRNAAll <- left_join(baseDesign,designRNA,"Specimen") %>% 
  filter(!is.na(DDA)) %>% 
  filter(!is.na(DIA)) %>% 
  filter(!is.na(Sample_title))
write_tsv(x = designRNAAll,file = 'RNA-seq/designRNA.tsv')
```

## 2.3 Differential Expression of Single AIMS PAM50 subtypes

This design file is used for differential expression analysis when
comparing 1-vs-rest. Since only DIA data was used for this purpose, the
clinical DIA design file is used as input.


```
designClinical <- read_tsv('DIA/DesignFilesDIA/design_aim2_clinical.tsv')
designRNA <- read_tsv('RNA-seq/designRNA.tsv') %>% 
  dplyr::select(Position,Sample_title)

mergedDesign <- left_join(x = designRNA,y = designClinical,"Position")
NewValues <- mergedDesign$AIMS_PAM50 %>% paste0('AIMS_PAM50.',.)
mergedDesign$AIMS_PAM50 <- NewValues
mergedDesign$AIMS_PAM50 <- as_factor(mergedDesign$AIMS_PAM50)
mergedDesign <- mergedDesign %>% mutate(one=1)
mergedDesign <- mergedDesign %>% pivot_wider(names_from = AIMS_PAM50,values_from = one,values_fill = 0) %>% mutate(.,PAM50=mergedDesign$AIMS_PAM50)

write_tsv(x = mergedDesign,file = 'DIA/DesignFilesDIA/design_aim2_clinical_matched_individualsubtypes.tsv')
```

## 2.4 Differential Expression of subtypes and clinicopathological features for Decision Tree model

This design file is used for differential expression analysis of
features used in the decision tree model. Only DIA data is used here, so
the clinical DIA file is used as input


```
design <- read_tsv('DIA/DesignFilesDIA/design_aim2_clinical.tsv') %>% 
  filter(!is.na(`LN status`)) %>% 
  filter(!is.na(Grade)) %>% 
  filter(!is.na(`HER2 status`)) %>% 
  filter(!is.na(AIMS_PAM50))

fixColNames <- colnames(design) %>% 
  str_replace_all(string = .,pattern = '\\ ',replacement = '\\_')
colnames(design) <- fixColNames

write_tsv(x = design,file = 'DIA/DesignFilesDIA/design_aim2_clinical_DecisionTree.tsv')
```

# 3 Data Pre-processing

## 3.1 DIA

### 3.1.1 DIA-NN

#### 3.1.1.1 Data Clean-up


```
dataP <- 'DIA/DataDE/DIANN/DIAscanb.pr_matrix.tsv'
dataClean <- read_tsv(dataP) %>% 
  na_if(.,0.0) %>% 
  na_if(.,0)
dataReduced <- dataClean %>% 
  dplyr::select(-c(1:10))
newNames <- colnames(dataReduced) %>% 
  str_extract(string = .,pattern = 'B[1-2][ABCDEFGHI][1-9]|Pool.*') %>% 
  str_replace(string = .,pattern = 'Pool\\_DIA\\_FullWindow\\_0',replacement = 'P') %>% 
  str_remove(string = .,pattern = '\\.mzML\\.dia')
colnames(dataReduced) <- newNames
dataClean <- dataClean %>% 
  dplyr::select(c(1:10)) %>% 
  bind_cols(.,dataReduced)

write_tsv(x = dataClean,file = 'DIA/DataDE/DIANN/DIAscanb.pr_matrix_clean.tsv')
```

#### 3.1.1.2 Peptide-level Normalization


```
jobName <- 'DIA_DIANN_peptide_normalisation'
dataP <- 'DIA/DataDE/DIANN/DIAscanb.pr_matrix_clean.tsv'
designP <- 'DIA/DesignFilesDIA/design_aim2_clinical.tsv'
outputP <- 'DIA/DataDE/DIANN'

normalyzer(jobName = jobName,designPath = designP,dataPath = dataP,outputDir = outputP,normalizeRetentionTime = FALSE,zeroToNA = TRUE,sampleColName = 'Position',groupColName = 'group')
```

#### 3.1.1.3 Sort Peptides Alphabetically


```
sortIds <- function(data,proteinColumn = 'Protein.Ids',...){
  data <- pull(data,proteinColumn) %>% 
    str_split(string = .,pattern = ';') %>% 
    map(.x = .,.f = str_sort) %>% 
    map(.x = .,.f = function(x) paste0(x,collapse = ';')) %>% 
    unlist %>% 
    mutate(data,Protein.ID=.,.before=colnames(data[2])) %>% 
    dplyr::select(-proteinColumn)
  names(data)[names(data)=='Protein.ID'] <- 'Protein'
  return(data)
}
```


```
data <- read_tsv(file = 'DIA/DataDE/DIANN/DIA_DIANN_peptide_normalisation/CycLoess-normalized.txt') %>%
  sortIds(data = .,proteinColumn = 'Protein.Ids')

write_tsv(x = data,file = 'DIA/DataDE/DIANN/DIA_DIANN_peptide_normalisation/CycLoess_norm_sorted.tsv')
```

#### 3.1.1.4 Protein Rollup


```
Rscript ~/SCANB_PUBLICATION/Algorithms/ProteinRollup-master/R/protein_rollup.R \
    --rdf_fp ~/SCANB_PUBLICATION/DIA/DataDE/DIANN/DIA_DIANN_peptide_normalisation/CycLoess_norm_sorted.tsv \
    --ddf_fp ~/SCANB_PUBLICATION/DIA/DesignFilesDIA/design_aim2_clinical.tsv \
    --sample_col Position \
    --protein_col Protein \
    --out_fp ~/SCANB_PUBLICATION/DIA/DataDE/DIANN/DIA_DIANN_Protein_RRollup/DIA_DIANN_RRollup.tsv
```

#### 3.1.1.5 Adding Gene Information


```
diaP <- 'DIA/DataDE/DIANN/DIA_DIANN_Protein_RRollup/DIA_DIANN_RRollup.tsv'
diannP <- 'DIA/DataDE/DIANN/DIA_DIANN_peptide_normalisation/CycLoess_norm_sorted.tsv'
diaData <- read_tsv(diaP)
diann <- read_tsv(diannP)
diannReduced <- diann %>% 
  dplyr::select(Protein,Protein.Names,First.Protein.Description,Genes)
diaDataGenes <- left_join(x = diaData,y = diannReduced,by = "Protein") %>% 
  filter(.,!duplicated(Protein))
diaDataGenes <- relocate(diaDataGenes,where(~is.character(.x)),.after = 'Protein')
write_tsv(x = diaDataGenes,file = 'DIA/DataDE/DIANN/DIA_DIANN_Protein_RRollup/DIA_DIANN_RRollup_Genes.tsv')
```

### 3.1.2 EncyclopeDIA

#### 3.1.2.1 Data Clean-up


```
dataP <- 'DIA/DataDE/EncyclopeDIA/SCANB_twoplates.peptides.txt'
read_tsv(dataP) %>% 
  na_if(.,0.0) %>% 
  na_if(.,0) %>% 
  relocate(.,'Protein',.before='Peptide') %>% 
  write_tsv(x = .,file = 'DIA/DataDE/EncyclopeDIA/SCANB_twoplates.peptides_clean.txt')
```

#### 3.1.2.2 Peptide-level Normalization


```
jobName <- 'DIA_EncyclopeDIA_peptide_normalisation'
dataP <- 'DIA/DataDE/EncyclopeDIA/SCANB_twoplates.peptides_clean.txt'
designP <- 'DIA/DesignFilesDIA/design_aim2_clinical.tsv'
outputP <- 'DIA/DataDE/EncyclopeDIA'

normalyzer(jobName = jobName,designPath = designP,dataPath = dataP,outputDir = outputP,normalizeRetentionTime = FALSE,zeroToNA = FALSE,sampleColName = 'DIA',groupColName = 'group')
```

#### 3.1.2.3 Sort Peptides Alphabetically


```
sortIds <- function(data,proteinColumn = 'Protein',...){
  data <- pull(data,proteinColumn) %>% 
    str_split(string = .,pattern = ';') %>% 
    map(.x = .,.f = str_sort) %>% 
    map(.x = .,.f = function(x) paste0(x,collapse = ';')) %>% 
    unlist %>% 
    mutate(data,Protein.ID=.,.before=colnames(data[2])) %>% 
    dplyr::select(-proteinColumn)
  names(data)[names(data)=='Protein.ID'] <- 'Protein'
  return(data)
}
```


```
data <- read_tsv(file = 'DIA/DataDE/EncyclopeDIA/DIA_EncyclopeDIA_peptide_normalisation/CycLoess-normalized.txt') %>% 
  sortIds(data = .,proteinColumn = 'Protein')

write_tsv(x = data,file = 'DIA/DataDE/EncyclopeDIA/DIA_EncyclopeDIA_peptide_normalisation/CycLoess_norm_sorted.tsv')
```

#### 3.1.2.4 Protein Rollup


```
Rscript ~/SCANB_PUBLICATION/Algorithms/ProteinRollup-master/R/protein_rollup.R \
    --rdf_fp ~/SCANB_PUBLICATION/DIA/DataDE/EncyclopeDIA/DIA_EncyclopeDIA_peptide_normalisation/CycLoess_norm_sorted.tsv \
    --ddf_fp ~/SCANB_PUBLICATION/DIA/DesignFilesDIA/design_aim2_clinical.tsv \
    --sample_col DIA \
    --protein_col Protein \
    --out_fp ~/SCANB_PUBLICATION/DIA/DataDE/EncyclopeDIA/DIA_EncyclopeDIA_Protein_RRolup/DIA_EncyclopeDIA_RRollup.tsv
```

#### 3.1.2.5 Adding Gene Information


```
# Setting BiomaRt
HSapiensMart <- useMart(biomart = 'ENSEMBL_MART_ENSEMBL')
HSapiensMart <- useDataset(dataset = 'hsapiens_gene_ensembl',mart = HSapiensMart)
```


```
dataDIA <- read_tsv(file = 'DIA/DataDE/EncyclopeDIA/DIA_EncyclopeDIA_Protein_RRolup/DIA_EncyclopeDIA_RRollup.tsv')
cleanIDs <- dataDIA$Protein %>% 
  str_split(string = .,pattern = ';') %>% 
  str_extract_all(string = .,pattern = '(?<=[:symbol:])[:alnum:]+(?=[:symbol:])') %>% 
  map(.x = .,.f = ~paste0(.x,collapse = ';')) %>%
  unlist
dataDIA$Protein <- cleanIDs

# Add Gene Information
proteinIdsDIA <- dataDIA$Protein %>% 
  str_split(string = .,pattern = ';')
names(proteinIdsDIA) <- dataDIA$Protein
geneNamesDIA <- map(.x = proteinIdsDIA,.f = ~getBM(mart=HSapiensMart,values = .x,attributes = 'uniprot_gn_symbol',filters = 'uniprotswissprot')) %>%
  map(.,unlist)

DIAGeneNames <- geneNamesDIA %>% 
  map(.x = .,.f = ~paste0(.x,collapse = ';')) %>%
  unname %>% 
  unlist
dataDIA <- dataDIA %>% 
  mutate(Gene=DIAGeneNames,.after = Protein)
write_tsv(x = dataDIA,file = 'DIA/DataDE/EncyclopeDIA/DIA_EncyclopeDIA_Protein_RRolup/DIA_EncyclopeDIA_RRollup_Genes.tsv')
```


```
# Short names for the samples
data <- read_tsv(file = 'DIA/DataDE/EncyclopeDIA/DIA_EncyclopeDIA_Protein_RRolup/DIA_EncyclopeDIA_RRollup_Genes.tsv')
design <- read_tsv(file = 'DIA/DesignFilesDIA/design_aim2_clinical.tsv')

colnames(data) <- colnames(data) %>% 
  str_remove(string = .,pattern = '^X(?=[:alnum:])')

dataReduced <- data %>% 
  dplyr::select(design$DIA)
colnames(dataReduced) <- design$Position

newData <- data %>% 
  dplyr::select(c(1:4)) %>% 
  bind_cols(.,dataReduced)

write_tsv(x = newData,file = 'DIA/DataDE/EncyclopeDIA/DIA_EncyclopeDIA_Protein_RRolup/DIA_EncyclopeDIA_RRollup_Genes.tsv')
```

## 3.2 DDA

### 3.2.1 DDA Data for Correlation

#### 3.2.1.1 Match Between Runs

##### 3.2.1.1.1 Prepare Files with shortnames


```
dataPMBR <- 'DDA/DataCorrelation/MBR/scanb_aim2_uniprot_mbr/peptides.txt'
dataMBR <- read_tsv(dataPMBR,col_names = TRUE) %>% 
  dplyr::select(Proteins,`Leading razor protein`,Sequence,contains('LFQ'))
dataMBRReduced <- dataMBR %>%
  dplyr::select(contains('LFQ'))
dataMBRNames <- dataMBRReduced %>% 
  colnames %>% 
  str_extract(string = .,pattern = 'B[:digit:].+|Pool.*') %>% 
  str_replace(string = .,pattern = 'Pool.*',replacement = 'Pool_DDA_01')
colnames(dataMBRReduced) <- dataMBRNames
dataMBR <- dataMBR %>% 
  transmute(Proteins=Proteins,Leading.Razor.Protein=`Leading razor protein`,Sequence=Sequence,dataMBRReduced)
dataMBR <- dataMBR %>% 
  filter(str_detect(string = Proteins,pattern = 'CON\\_',negate = TRUE))

write_tsv(x = dataMBR,file = 'DDA/DataCorrelation/MBR/peptides_mbr_shortnames.tsv')
```

##### 3.2.1.1.2 Peptide-level Normalization


```
jobMBR <- 'DDA_MBR_peptide_normalisation'
dataPMBR <- 'DDA/DataCorrelation/MBR/peptides_mbr_shortnames.tsv'
designP <- 'DDA/DesignFilesDDA/design_aim2_clinical.tsv'
outputPMBR <- 'DDA/DataCorrelation/MBR'

normalyzer(jobName = jobMBR,designPath = designP,dataPath = dataPMBR,outputDir = outputPMBR,requireReplicates = FALSE,normalizeRetentionTime = FALSE,zeroToNA = TRUE,sampleColName = 'Position',groupColName = 'group')
```

##### 3.2.1.1.3 Sort Peptides Alphabetically


```
sortIds <- function(data,proteinColumn = 'Proteins',...){
  data <- pull(data,proteinColumn) %>% 
    str_split(string = .,pattern = ';') %>% 
    map(.x = .,.f = str_sort) %>% 
    map(.x = .,.f = function(x) paste0(x,collapse = ';')) %>% 
    unlist %>% 
    mutate(data,Protein.ID=.,.before=colnames(data[2])) %>% 
    dplyr::select(-proteinColumn)
  names(data)[names(data)=='Protein.ID'] <- 'Protein'
  return(data)
}
```


```
dataMBR <- read_tsv(file = 'DDA/DataCorrelation/MBR/DDA_MBR_peptide_normalisation/CycLoess-normalized.txt') %>% 
  sortIds(data = .,proteinColumn = 'Proteins')

write_tsv(x = dataMBR,file = 'DDA/DataCorrelation/MBR/DDA_MBR_peptide_normalisation/CycLoess_norm_sorted.tsv')
```

##### 3.2.1.1.4 Protein Rollup


```
Rscript ~/SCANB_PUBLICATION/Algorithms/ProteinRollup-master/R/protein_rollup.R \
    --rdf_fp ~/SCANB_PUBLICATION/DDA/DataCorrelation/MBR/DDA_MBR_peptide_normalisation/CycLoess_norm_sorted.tsv \
    --ddf_fp ~/SCANB_PUBLICATION/DDA/DesignFilesDDA/design_aim2_clinical.tsv \
    --sample_col Position \
    --protein_col Protein \
    --out_fp ~/SCANB_PUBLICATION/DDA/DataCorrelation/MBR/DDA_MBR_Protein_Rollup/DDA_MBR_RRollup.tsv
```

##### 3.2.1.1.5 Add Gene Information


```
HSapiensMart <- useMart(biomart = 'ENSEMBL_MART_ENSEMBL')
HSapiensMart <- useDataset(dataset = 'hsapiens_gene_ensembl',mart = HSapiensMart)
```


```
dataMBR <- read_tsv(file = 'DDA/DataCorrelation/MBR/DDA_MBR_Protein_Rollup/DDA_MBR_RRollup.tsv')
proteinIdsMBR <- dataMBR$Protein %>% 
  str_split(string = .,pattern = ';')
names(proteinIdsMBR) <- dataMBR$Protein
geneNamesMBR <- map(.x = proteinIdsMBR,.f = ~getBM(mart=HSapiensMart,values = .x,attributes = 'uniprot_gn_symbol',filters = 'uniprotswissprot')) %>%
  map(.,unlist)

MBRGeneNames <- geneNamesMBR %>% 
  map(.x = .,.f = ~paste0(.x,collapse = ';')) %>%
  unname %>% 
  unlist
dataMBR <- dataMBR %>% 
  mutate(Gene=MBRGeneNames,.after = Protein)
write_tsv(x = dataMBR,file = 'DDA/DataCorrelation/MBR/DDA_MBR_Protein_Rollup/DDA_MBR_RRollup_Genes.tsv')
```

#### 3.2.1.2 No Match Between Runs

##### 3.2.1.2.1 Prepare Files with short names


```
dataPnoMBR <- 'DDA/DataCorrelation/noMBR/scanb_aim2_uniprot_no_mbr/peptides.txt'
dataNoMBR <- read_tsv(dataPnoMBR,col_names = TRUE) %>% 
  dplyr::select(Proteins,`Leading razor protein`,Sequence,contains('LFQ'))
dataNoMBRReduced <- dataNoMBR %>%
  dplyr::select(contains('LFQ'))
dataNoMBRNames <- dataNoMBRReduced %>% 
  colnames %>% 
  str_extract(string = .,pattern = 'B[:digit:].+|Pool.*') %>% 
  str_replace(string = .,pattern = 'Pool.*',replacement = 'Pool_DDA_01')
colnames(dataNoMBRReduced) <- dataNoMBRNames
dataNoMBR <- dataNoMBR %>% 
  transmute(Proteins=Proteins,Leading.Razor.Protein=`Leading razor protein`,Sequence=Sequence,dataNoMBRReduced)
dataNoMBR <- dataNoMBR %>% 
  filter(str_detect(string = Proteins,pattern = 'CON\\_',negate = TRUE))

write_tsv(x = dataNoMBR,file = 'DDA/DataCorrelation/noMBR/peptides_nombr_shortnames.tsv')
```

##### 3.2.1.2.2 Peptide-level Normalization


```
jobNoMBR <- 'DDA_no_MBR_peptide_normalisation'
dataPNoMBR <- 'DDA/DataCorrelation/noMBR/peptides_nombr_shortnames.tsv'
designP <- 'DDA/DesignFilesDDA/design_aim2_clinical.tsv'
outputPNoMBR <- 'DDA/DataCorrelation/noMBR'

normalyzer(jobName = jobNoMBR,designPath = designP,dataPath = dataPNoMBR,outputDir = outputPNoMBR,requireReplicates = FALSE,normalizeRetentionTime = FALSE,zeroToNA = TRUE,sampleColName = 'Position',groupColName = 'group')
```

##### 3.2.1.2.3 Sort Peptides Alphabetically


```
sortIds <- function(data,proteinColumn = 'Proteins',...){
  data <- pull(data,proteinColumn) %>% 
    str_split(string = .,pattern = ';') %>% 
    map(.x = .,.f = str_sort) %>% 
    map(.x = .,.f = function(x) paste0(x,collapse = ';')) %>% 
    unlist %>% 
    mutate(data,Protein.ID=.,.before=colnames(data[2])) %>% 
    dplyr::select(-proteinColumn)
  names(data)[names(data)=='Protein.ID'] <- 'Protein'
  return(data)
}
```


```
dataNoMBR <- read_tsv(file = 'DDA/DataCorrelation/noMBR/DDA_no_MBR_peptide_normalisation/CycLoess-normalized.txt') %>% 
  sortIds(data = .,proteinColumn = 'Proteins')

write_tsv(x = dataNoMBR,file = 'DDA/DataCorrelation/noMBR/DDA_no_MBR_peptide_normalisation/CycLoess_norm_sorted.tsv')
```

##### 3.2.1.2.4 Protein Rollup


```
Rscript ~/SCANB_PUBLICATION/Algorithms/ProteinRollup-master/R/protein_rollup.R \
    --rdf_fp ~/SCANB_PUBLICATION/DDA/DataCorrelation/noMBR/DDA_no_MBR_peptide_normalisation/CycLoess_norm_sorted.tsv \
    --ddf_fp ~/SCANB_PUBLICATION/DDA/DesignFilesDDA/design_aim2_clinical.tsv \
    --sample_col Position \
    --protein_col Protein \
    --out_fp ~/SCANB_PUBLICATION/DDA/DataCorrelation/noMBR/DDA_noMBR_Protein_RRollup/DDA_noMBR_RRolup.tsv
```

##### 3.2.1.2.5 Add Gene Information


```
HSapiensMart <- useMart(biomart = 'ENSEMBL_MART_ENSEMBL')
HSapiensMart <- useDataset(dataset = 'hsapiens_gene_ensembl',mart = HSapiensMart)
```


```
dataNoMBR <- read_tsv(file = 'DDA/DataCorrelation/noMBR/DDA_noMBR_Protein_RRollup/DDA_noMBR_RRolup.tsv')
proteinIdsNoMBR <- dataNoMBR$Protein %>% 
  str_split(string = .,pattern = ';')
names(proteinIdsNoMBR) <- dataNoMBR$Protein
geneNamesNoMBR <- map(.x = proteinIdsNoMBR,.f = ~getBM(mart=HSapiensMart,values = .x,attributes = 'uniprot_gn_symbol',filters = 'uniprotswissprot')) %>%
  map(.,unlist)

NoMBRGeneNames <- geneNamesNoMBR %>% 
  map(.x = .,.f = ~paste0(.x,collapse = ';')) %>% 
  unname %>% 
  unlist
dataNoMBR <- dataNoMBR %>% 
  mutate(Gene=NoMBRGeneNames,.after=Protein)
write_tsv(x = dataNoMBR,file = 'DDA/DataCorrelation/noMBR/DDA_noMBR_Protein_RRollup/DDA_noMBR_RRolup_Genes.tsv')
```

### 3.2.2 Match Between Runs

#### 3.2.2.1 Prepare Files with short names


```
dataPMBR <- 'DDA/DataDE/MaxQuant/MBR/scanb_aim2_thisp2_mbr/peptides.txt'
dataMBR <- read_tsv(dataPMBR,col_names = TRUE,guess_max = 10000) %>% 
  dplyr::select(Proteins,`Leading razor protein`,Sequence,contains('LFQ'))
dataMBRReduced <- dataMBR %>%
  dplyr::select(contains('LFQ'))
dataMBRNames <- dataMBRReduced %>% 
  colnames %>% 
  str_extract(string = .,pattern = 'B[:digit:].+|Pool.*') %>% 
  str_replace(string = .,pattern = 'Pool.*',replacement = 'Pool_DDA_01')
colnames(dataMBRReduced) <- dataMBRNames
dataMBR <- dataMBR %>% 
  transmute(Proteins=Proteins,Leading.Razor.Protein=`Leading razor protein`,Sequence=Sequence,dataMBRReduced)
dataMBR <- dataMBR %>% 
  filter(str_detect(string = Proteins,pattern = 'CON\\_',negate = TRUE)) %>% 
  filter(str_detect(string = Proteins,pattern = 'CONTAM\\_',negate = TRUE))

write_tsv(x = dataMBR,file = 'DDA/DataDE/MaxQuant/MBR/peptides_mbr_shortnames.tsv')
```

#### 3.2.2.2 Peptide-level Normalization


```
jobMBR <- 'DDA_MBR_peptide_normalisation'
dataPMBR <- 'DDA/DataDE/MaxQuant/MBR/peptides_mbr_shortnames.tsv'
designP <- 'DDA/DesignFilesDDA/design_aim2_clinical.tsv'
outputPMBR <- 'DDA/DataDE/MaxQuant/MBR'

normalyzer(jobName = jobMBR,designPath = designP,dataPath = dataPMBR,outputDir = outputPMBR,requireReplicates = FALSE,normalizeRetentionTime = FALSE,zeroToNA = TRUE,sampleColName = 'Position',groupColName = 'group')
```

#### 3.2.2.3 Sort Peptides Alphabetically


```
sortIds <- function(data,proteinColumn = 'Proteins',...){
  data <- pull(data,proteinColumn) %>% 
    str_split(string = .,pattern = ';') %>% 
    map(.x = .,.f = str_sort) %>% 
    map(.x = .,.f = function(x) paste0(x,collapse = ';')) %>% 
    unlist %>% 
    mutate(data,Protein.ID=.,.before=colnames(data[2])) %>% 
    dplyr::select(-proteinColumn)
  names(data)[names(data)=='Protein.ID'] <- 'Protein'
  return(data)
}
```


```
dataMBR <- read_tsv(file = 'DDA/DataDE/MaxQuant/MBR/DDA_MBR_peptide_normalisation/CycLoess-normalized.txt') %>% 
  sortIds(data = .,proteinColumn = 'Proteins')

write_tsv(x = dataMBR,file = 'DDA/DataDE/MaxQuant/MBR/DDA_MBR_peptide_normalisation/CycLoess_norm_sorted.tsv')
```

#### 3.2.2.4 Protein Rollup


```
Rscript ~/SCANB_PUBLICATION/Algorithms/ProteinRollup-master/R/protein_rollup.R \
    --rdf_fp ~/SCANB_PUBLICATION/DDA/DataDE/MaxQuant/MBR/DDA_MBR_peptide_normalisation/CycLoess_norm_sorted.tsv \
    --ddf_fp ~/SCANB_PUBLICATION/DDA/DesignFilesDDA/design_aim2_clinical.tsv \
    --sample_col Position \
    --protein_col Protein \
    --out_fp ~/SCANB_PUBLICATION/DDA/DataDE/MaxQuant/MBR/DDA_MBR_Protein_RRollup/DDA_MBR_RRollup.tsv
```

### 3.2.3 No Match Between Runs

#### 3.2.3.1 Prepare Files with short names


```
dataPnoMBR <- 'DDA/DataDE/MaxQuant/NoMBR/scanb_aim2_thisp2_no_mbr/peptides.txt'
dataNoMBR <- read_tsv(dataPnoMBR,col_names = TRUE,guess_max = 10000) %>% 
  dplyr::select(Proteins,`Leading razor protein`,Sequence,contains('LFQ'))
dataNoMBRReduced <- dataNoMBR %>%
  dplyr::select(contains('LFQ'))
dataNoMBRNames <- dataNoMBRReduced %>% 
  colnames %>% 
  str_extract(string = .,pattern = 'B[:digit:].+|Pool.*') %>% 
  str_replace(string = .,pattern = 'Pool.*',replacement = 'Pool_DDA_01')
colnames(dataNoMBRReduced) <- dataNoMBRNames
dataNoMBR <- dataNoMBR %>% 
  transmute(Proteins=Proteins,Leading.Razor.Protein=`Leading razor protein`,Sequence=Sequence,dataNoMBRReduced)
dataNoMBR <- dataNoMBR %>% 
  filter(str_detect(string = Proteins,pattern = 'CON\\_',negate = TRUE)) %>% 
  filter(str_detect(string = Proteins,pattern = 'CONTAM\\_',negate = TRUE))

write_tsv(x = dataNoMBR,file = 'DDA/DataDE/MaxQuant/NoMBR/peptides_nombr_shortnames.tsv')
```

#### 3.2.3.2 Peptide-level Normalization


```
jobNoMBR <- 'DDA_no_MBR_peptide_normalisation'
dataPNoMBR <- 'DDA/DataDE/MaxQuant/NoMBR/peptides_nombr_shortnames.tsv'
designP <- 'DDA/DesignFilesDDA/design_aim2_clinical.tsv'
outputPNoMBR <- 'DDA/DataDE/MaxQuant/NoMBR'

normalyzer(jobName = jobNoMBR,designPath = designP,dataPath = dataPNoMBR,outputDir = outputPNoMBR,requireReplicates = FALSE,normalizeRetentionTime = FALSE,zeroToNA = TRUE,sampleColName = 'Position',groupColName = 'group')
```

#### 3.2.3.3 Sort Peptides Alphabetically


```
sortIds <- function(data,proteinColumn = 'Proteins',...){
  data <- pull(data,proteinColumn) %>% 
    str_split(string = .,pattern = ';') %>% 
    map(.x = .,.f = str_sort) %>% 
    map(.x = .,.f = function(x) paste0(x,collapse = ';')) %>% 
    unlist %>% 
    mutate(data,Protein.ID=.,.before=colnames(data[2])) %>% 
    dplyr::select(-proteinColumn)
  names(data)[names(data)=='Protein.ID'] <- 'Protein'
  return(data)
}
```


```
dataNoMBR <- read_tsv(file = 'DDA/DataDE/MaxQuant/NoMBR/DDA_no_MBR_peptide_normalisation/CycLoess-normalized.txt') %>% 
  sortIds(data = .,proteinColumn = 'Proteins')

write_tsv(x = dataNoMBR,file = 'DDA/DataDE/MaxQuant/NoMBR/DDA_no_MBR_peptide_normalisation/CycLoess_norm_sorted.tsv')
```

#### 3.2.3.4 Protein Rollup


```
Rscript ~/SCANB_PUBLICATION/Algorithms/ProteinRollup-master/R/protein_rollup.R \
    --rdf_fp ~/SCANB_PUBLICATION/DDA/DataDE/MaxQuant/NoMBR/DDA_no_MBR_peptide_normalisation/CycLoess_norm_sorted.tsv \
    --ddf_fp ~/SCANB_PUBLICATION/DDA/DesignFilesDDA/design_aim2_clinical.tsv \
    --sample_col Position \
    --protein_col Protein \
    --out_fp ~/SCANB_PUBLICATION/DDA/DataDE/MaxQuant/NoMBR/DDA_noMBR_Protein_RRollup/DDA_noMBR_RRollup.tsv
```

## 3.3 RNA-Seq

### 3.3.1 Preprocessing

This portion is run in order to generate smaller data files
containing only RNA-seq samples that matches those present in DDA and
DIA analyses.


```
rnaData <- read_csv(file = 'RNA-seq/GSE96058/GSE96058_gene_expression_3273_samples_and_136_replicates_transformed.csv')
RNANames <- colnames(rnaData) %>% 
  str_replace(string = .,pattern = '\\...1',replacement = 'Gene')
colnames(rnaData) <- RNANames

designRNA <- read_tsv('RNA-seq/designRNA.tsv')

rnaDataReduced <- rnaData %>% 
  dplyr::select(Gene,designRNA$Sample_title)
write_tsv(x = rnaDataReduced,file = 'RNA-seq/GSE96058_gene_expression_Reduced.tsv')
```

# 4 Correlations

## 4.1 Function


```
correlation_datasets <- function(data1,data2,...){
  require(psych)
  data1 <- t(data1)
  data2 <- t(data2)
  spearman_correlation <- map(.x = 1:ncol(data1),.f = function(i) corr.test(data1[,i],data2[,i],method='spearman',ci=FALSE,adjust = 'none'))
  pearson_correlation <- map(.x = 1:ncol(data1),.f = function(i) corr.test(data1[,i],data2[,i],method='pearson',ci=FALSE,adjust = 'none'))
  spearman_r <- map(spearman_correlation,~pluck(.x,'r')) %>% unlist(.)
  spearman_p <- map(spearman_correlation,~pluck(.x,'p')) %>% unlist(.)
  pearson_r <- map(pearson_correlation,~pluck(.x,'r')) %>% unlist(.)
  pearson_p <- map(pearson_correlation,~pluck(.x,'p')) %>% unlist(.)
  results <- tibble(Spearman_Score = spearman_r,p.value.spearman = spearman_p,Pearson_Score = pearson_r,p.value.pearson = pearson_p)
  return(results)
}
```

## 4.2 Overall

### 4.2.1 EncyclopeDIA vs RNA-seq


```
diaP <- 'DIA/DataDE/EncyclopeDIA/DIA_EncyclopeDIA_Protein_RRolup/DIA_EncyclopeDIA_RRollup_Genes.tsv'
designP <- 'RNA-seq/designRNA.tsv'
rnaDIA <- 'RNA-seq/GSE96058_gene_expression_Reduced.tsv' %>% 
  read_tsv

design <- read_tsv(designP) %>% 
  filter(!is.na(Sample_title))
dataDIA <- read_tsv(diaP) %>% 
  dplyr::select(1:3,Gene,design$Position)

mergedData <- inner_join(dataDIA,rnaDIA,"Gene")

diaDataReduced <- mergedData %>% 
  dplyr::select(design$Position)
rnaDataReduced <- mergedData %>% 
  dplyr::select(design$Sample_title)

correlationRnaEncyclopedia <- correlation_datasets(diaDataReduced,rnaDataReduced) %>% 
  mutate(.,Protein = mergedData$Protein, Gene=mergedData$Gene, P.Perason.Adj=p.adjust(p.value.pearson,method = 'fdr'),P.Spearman.Adj=p.adjust(p.value.spearman,method = 'fdr'))
write_tsv(x = correlationRnaEncyclopedia,file = 'Correlations/correlation_RNA_EncyclopeDIA.tsv')
```

### 4.2.2 DIA-NN vs RNA-seq


```
diaP <- 'DIA/DataDE/DIANN/DIA_DIANN_Protein_RRollup/DIA_DIANN_RRollup_Genes.tsv'
designP <- 'RNA-seq/designRNA.tsv'
rnaDIA <- 'RNA-seq/GSE96058_gene_expression_Reduced.tsv' %>% 
  read_tsv

design <- read_tsv(designP) %>% 
  filter(!is.na(Sample_title))
dataDIA <- read_tsv(diaP) %>% 
  dplyr::select(1:3,Gene=Genes,design$Position)

mergedData <- inner_join(dataDIA,rnaDIA,"Gene")

diaDataReduced <- mergedData %>% 
  dplyr::select(design$Position)
rnaDataReduced <- mergedData %>% 
  dplyr::select(design$Sample_title)

correlationRnaDIANN <- correlation_datasets(diaDataReduced,rnaDataReduced) %>% 
  mutate(.,Protein = mergedData$Protein, Gene=mergedData$Gene, P.Perason.Adj=p.adjust(p.value.pearson,method = 'fdr'),P.Spearman.Adj=p.adjust(p.value.spearman,method = 'fdr'))
write_tsv(x = correlationRnaDIANN,file = 'Correlations/correlation_RNA_DIANN.tsv')
```

### 4.2.3 DDA Match Between Runs vs RNA-seq


```
ddaP <- 'DDA/DataCorrelation/MBR/DDA_MBR_Protein_Rollup/DDA_MBR_RRollup_Genes.tsv'
designP <- 'RNA-seq/designRNA.tsv'
rnaDDA <- 'RNA-seq/GSE96058_gene_expression_Reduced.tsv' %>% 
  read_tsv

design <- read_tsv(designP) %>% 
  filter(!is.na(Sample_title))
dataDDA <- read_tsv(ddaP) %>% 
  dplyr::select(1:3,Gene,design$Position)

mergedData <- inner_join(dataDDA,rnaDDA,"Gene")

ddaDataReduced <- mergedData %>% 
  dplyr::select(design$Position)
rnaDataReduced <- mergedData %>% 
  dplyr::select(design$Sample_title)

correlationRnaDDAMBR <- correlation_datasets(ddaDataReduced,rnaDataReduced) %>% 
  mutate(.,Protein = mergedData$Protein, Gene=mergedData$Gene, P.Perason.Adj=p.adjust(p.value.pearson,method = 'fdr'),P.Spearman.Adj=p.adjust(p.value.spearman,method = 'fdr'))
write_tsv(x = correlationRnaDDAMBR,file = 'Correlations/correlation_RNA_DDA_MBR.tsv')
```

### 4.2.4 DDA no Match Between Runs vs RNA-seq


```
ddaP <- 'DDA/DataCorrelation/noMBR/DDA_noMBR_Protein_RRollup/DDA_noMBR_RRolup_Genes.tsv'
designP <- 'RNA-seq/designRNA.tsv'
rnaDDA <- 'RNA-seq/GSE96058_gene_expression_Reduced.tsv' %>% 
  read_tsv

design <- read_tsv(designP) %>% 
  filter(!is.na(Sample_title))
dataDDA <- read_tsv(ddaP) %>% 
  dplyr::select(1:3,Gene,design$Position)

mergedData <- inner_join(dataDDA,rnaDDA,"Gene")

ddaDataReduced <- mergedData %>% 
  dplyr::select(design$Position)
rnaDataReduced <- mergedData %>% 
  dplyr::select(design$Sample_title)

correlationRnaDDANoMBR <- correlation_datasets(ddaDataReduced,rnaDataReduced) %>%
  mutate(.,Protein = mergedData$Protein, Gene=mergedData$Gene, P.Perason.Adj=p.adjust(p.value.pearson,method = 'fdr'),P.Spearman.Adj=p.adjust(p.value.spearman,method = 'fdr'))
write_tsv(x = correlationRnaDDANoMBR,file = 'Correlations/correlation_RNA_DDA_NoMBR.tsv')
```

## 4.3 After low variance removal

### 4.3.1 Function


```
medianFilteringProtRNA <- function(protData,rnaData,design,...){
  varianceNA <- function(data) var(data,na.rm=TRUE)

  mergedData <- inner_join(protData,rnaData,'Gene')
  
  varianceProt <- mergedData %>% 
    dplyr::select(design$Position) %>% 
    t %>% 
    as_tibble %>% 
    map_dfc(.,varianceNA) %>% 
    t
    
  varianceRNA <- mergedData %>% 
    dplyr::select(design$Sample_title) %>% 
    t %>% 
    as_tibble %>% 
    map_dfc(.,varianceNA) %>% 
    t

  mergedData <- mergedData %>% 
    mutate(.,VarProt=varianceProt,VarRNA=varianceRNA)
  
  lowVarData <- mergedData %>% 
    filter(VarProt > median(VarProt,na.rm = T),VarRNA > median(VarRNA,na.rm = T))
  
  lowVarProt <- lowVarData %>% 
    dplyr::select(design$Position)
  lowVarRNA <- lowVarData %>% 
    dplyr::select(design$Sample_title)
  
  correlationLowVarFiltering <- correlation_datasets(data1 = lowVarProt,data2 = lowVarRNA) %>%
    mutate(.,Gene = lowVarData$Gene, P.Perason.Adj=p.adjust(p.value.pearson,method = 'fdr'),P.Spearman.Adj=p.adjust(p.value.spearman,method = 'fdr'))
  
  results <- list(lowVar=correlationLowVarFiltering)
  return(results)
}
```

### 4.3.2 EncyclopeDIA vs RNA-seq


```
diaP <- 'DIA/DataDE/EncyclopeDIA/DIA_EncyclopeDIA_Protein_RRolup/DIA_EncyclopeDIA_RRollup_Genes.tsv'
designP <- 'RNA-seq/designRNA.tsv'
rnaDIA <- 'RNA-seq/GSE96058_gene_expression_Reduced.tsv' %>% 
  read_tsv

design <- read_tsv(designP) %>% 
  filter(!is.na(Sample_title))
dataDIA <- read_tsv(diaP) %>% 
  dplyr::select(1:3,Gene,design$Position)

correlationFilterEncyclopediaRNA <- medianFilteringProtRNA(protData = dataDIA,rnaData = rnaDIA,design = design)
write_tsv(x = correlationFilterEncyclopediaRNA$lowVar,file = 'Correlations/correlation_RNA_EncyclopeDIA_LowVar.tsv')
```

### 4.3.3 DIA-NN vs RNA-seq


```
diaP <- 'DIA/DataDE/DIANN/DIA_DIANN_Protein_RRollup/DIA_DIANN_RRollup_Genes.tsv'
designP <- 'RNA-seq/designRNA.tsv'
rnaDIA <- 'RNA-seq/GSE96058_gene_expression_Reduced.tsv' %>% 
  read_tsv

design <- read_tsv(designP) %>% 
  filter(!is.na(Sample_title))
dataDIA <- read_tsv(diaP) %>% 
  dplyr::select(1:3,Gene=Genes,design$Position)

correlationFilterDIANNRNA <- medianFilteringProtRNA(protData = dataDIA,rnaData = rnaDIA,design = design)
write_tsv(x = correlationFilterDIANNRNA$lowVar,file = 'Correlations/correlation_DIANN_RNA_LowVar.tsv')
```

### 4.3.4 DDA Match Between Runs vs RNA-seq


```
ddaP <- 'DDA/DataCorrelation/MBR/DDA_MBR_Protein_Rollup/DDA_MBR_RRollup_Genes.tsv'
designP <-'RNA-seq/designRNA.tsv'
rnaDDA <- 'RNA-seq/GSE96058_gene_expression_Reduced.tsv' %>% 
  read_tsv

design <- read_tsv(designP) %>% 
  filter(!is.na(Sample_title))
dataDDA <- read_tsv(ddaP) %>% 
  dplyr::select(1:3,Gene,design$Position)

correlationRnaDDAMBR <- medianFilteringProtRNA(protData = dataDDA,rnaData = rnaDDA,design = design)
write_tsv(x = correlationRnaDDAMBR$lowVar,file = 'Correlations/correlation_RNA_DDA_MBR_LowVar.tsv')
```

### 4.3.5 DDA no Match Between Runs vs RNA-seq


```
ddaP <- 'DDA/DataCorrelation/noMBR/DDA_noMBR_Protein_RRollup/DDA_noMBR_RRolup_Genes.tsv'
designP <- 'RNA-seq/designRNA.tsv'
rnaDDA <- 'RNA-seq/GSE96058_gene_expression_Reduced.tsv' %>% 
  read_tsv

design <- read_tsv(designP) %>% 
  filter(!is.na(Sample_title))
dataDDA <- read_tsv(ddaP) %>% 
  dplyr::select(1:3,Gene,design$Position)

correlationRnaDDANoMBR <- medianFilteringProtRNA(protData = dataDDA,rnaData = rnaDDA,design = design)
write_tsv(x = correlationRnaDDANoMBR$lowVar,file = 'Correlations/correlation_RNA_DDA_NoMBR_LowVar.tsv')
```

## 4.4 Correlation Summary

### 4.4.1 Function


```
corrSummary <- function(data, ...){
  correlationSummary <- data %>% 
    summarise(.,Pairs=nrow(x = .[,5]),Proteins.FDR.Spearman = length(which(P.Spearman.Adj<0.05)),Median.Spearman.Score=median(Spearman_Score,na.rm = T),Proteins.FDR.Pearson = length(which(P.Perason.Adj<0.05)),Median.Pearson.Score=median(Pearson_Score,na.rm = T))
}
```

### 4.4.2 Correlation Summary of all available files


```
corrFiles <- list.files(pattern = '*.tsv',path = 'Correlations',full.names = T)
corrData <- map(.x = corrFiles,.f = read_tsv)
names(corrData) <- corrFiles
```


```
corrNames <- names(corrData) %>% 
  str_remove_all(pattern = 'Correlations\\/correlation\\_') %>% 
  str_replace_all(pattern = '\\_',replacement = ' ') %>% 
  str_remove_all(pattern = '\\.tsv')
  
summaryResults <- map_df(.x = corrData,.f = corrSummary) %>% 
  mutate(.,Correlation = corrNames,.before='Pairs')

write_tsv(x = summaryResults,file = 'Correlations/Correlation_summary.tsv')
```

# 5 Gene Set Enrichment Analysis (Figure 1)

## 5.1 Differential Expression Analysis

The differential expression analysis performed herein pertains the
different intrinsic subtypes (AIMS-PAM50), i.e. features are determined
based on 1-vs-others comparisons. The samples used were exactly the same
across different data sources.

### 5.1.1 DIA-NN Proteomics


```
# For Luminal A
dataP <- 'DIA/DataDE/DIANN/DIA_DIANN_Protein_RRollup/DIA_DIANN_RRollup_Genes.tsv'
designP <- 'DIA/DesignFilesDIA/design_aim2_clinical_matched_individualsubtypes.tsv'
outDir <- 'DE_analysis/DE_for_GSEA/'
jDEName <- "LumA vs rest Proteomics AIMS PAM50"

normalyzerDE(jobName = jDEName, designPath = designP, dataPath = dataP, leastRepCount = 1, sampleCol="Position", condCol = "AIMS_PAM50.LumA" , comparisons = c('1-0'), sigThres = 0.05, outputDir = outDir,batchCol = 'group')
```


```
# For Luminal B
dataP <- 'DIA/DataDE/DIANN/DIA_DIANN_Protein_RRollup/DIA_DIANN_RRollup_Genes.tsv'
designP <- 'DIA/DesignFilesDIA/design_aim2_clinical_matched_individualsubtypes.tsv'
outDir <- 'DE_analysis/DE_for_GSEA/'
jDEName <- "LumB vs rest Proteomics AIMS PAM50"

normalyzerDE(jobName = jDEName, designPath = designP, dataPath = dataP, leastRepCount = 2, sampleCol="Position", condCol = "AIMS_PAM50.LumB" , comparisons = c('1-0'), sigThres = 0.05, outputDir = outDir,batchCol = 'group')
```


```
# For Basal
dataP <- 'DIA/DataDE/DIANN/DIA_DIANN_Protein_RRollup/DIA_DIANN_RRollup_Genes.tsv'
designP <- 'DIA/DesignFilesDIA/design_aim2_clinical_matched_individualsubtypes.tsv'
outDir <- 'DE_analysis/DE_for_GSEA/'
jDEName <- "Basal vs rest Proteomics AIMS PAM50"

normalyzerDE(jobName = jDEName, designPath = designP, dataPath = dataP, leastRepCount = 2, sampleCol="Position", condCol = "AIMS_PAM50.Basal" , comparisons = c('1-0'), sigThres = 0.05, outputDir = outDir,batchCol = 'group')
```


```
# For Normal-like
dataP <- 'DIA/DataDE/DIANN/DIA_DIANN_Protein_RRollup/DIA_DIANN_RRollup_Genes.tsv'
designP <- 'DIA/DesignFilesDIA/design_aim2_clinical_matched_individualsubtypes.tsv'
outDir <- 'DE_analysis/DE_for_GSEA/'
jDEName <- "Normal vs rest Proteomics AIMS PAM50"

normalyzerDE(jobName = jDEName, designPath = designP, dataPath = dataP, leastRepCount = 1, sampleCol="Position", condCol = "AIMS_PAM50.Normal" , comparisons = c('1-0'), sigThres = 0.05, outputDir = outDir,batchCol = 'group')
```


```
# For HER2-enriched
dataP <- 'DIA/DataDE/DIANN/DIA_DIANN_Protein_RRollup/DIA_DIANN_RRollup_Genes.tsv'
designP <- 'DIA/DesignFilesDIA/design_aim2_clinical_matched_individualsubtypes.tsv'
outDir <- 'DE_analysis/DE_for_GSEA/'
jDEName <- "Her2 vs rest Proteomics AIMS PAM50"

normalyzerDE(jobName = jDEName, designPath = designP, dataPath = dataP, leastRepCount = 2, sampleCol="Position", condCol = "AIMS_PAM50.Her2" , comparisons = c('1-0'), sigThres = 0.05, outputDir = outDir,batchCol = 'group')
```

### 5.1.2 RNA-seq


```
# For Luminal A
dataP <- 'RNA-seq/GSE96058_gene_expression_Reduced.tsv'
designP <- 'DIA/DesignFilesDIA/design_aim2_clinical_matched_individualsubtypes.tsv'
outDir <- 'DE_analysis/DE_for_GSEA/'
jDEName <- "LumA vs rest RNAseq AIMS PAM50"

normalyzerDE(jobName = jDEName, designPath = designP, dataPath = dataP, leastRepCount = 1, sampleCol="Sample_title", condCol = "AIMS_PAM50.LumA" , comparisons = c('1-0'), sigThres = 0.05, outputDir = outDir,batchCol = 'group')
```


```
# For Luminal B
dataP <- 'RNA-seq/GSE96058_gene_expression_Reduced.tsv'
designP <- 'DIA/DesignFilesDIA/design_aim2_clinical_matched_individualsubtypes.tsv'
outDir <- 'DE_analysis/DE_for_GSEA/'
jDEName <- "LumB vs rest RNAseq AIMS PAM50"

normalyzerDE(jobName = jDEName, designPath = designP, dataPath = dataP, leastRepCount = 2, sampleCol="Sample_title", condCol = "AIMS_PAM50.LumB" , comparisons = c('1-0'), sigThres = 0.05, outputDir = outDir,batchCol = 'group')
```


```
# For Basal
dataP <- 'RNA-seq/GSE96058_gene_expression_Reduced.tsv'
designP <- 'DIA/DesignFilesDIA/design_aim2_clinical_matched_individualsubtypes.tsv'
outDir <- 'DE_analysis/DE_for_GSEA/'
jDEName <- "Basal vs rest RNAseq AIMS PAM50"

normalyzerDE(jobName = jDEName, designPath = designP, dataPath = dataP, leastRepCount = 2, sampleCol="Sample_title", condCol = "AIMS_PAM50.Basal" , comparisons = c('1-0'), sigThres = 0.05, outputDir = outDir,batchCol = 'group')
```


```
# For Normal-like
dataP <- 'RNA-seq/GSE96058_gene_expression_Reduced.tsv'
designP <- 'DIA/DesignFilesDIA/design_aim2_clinical_matched_individualsubtypes.tsv'
outDir <- 'DE_analysis/DE_for_GSEA/'
jDEName <- "Normal vs rest RNAseq AIMS PAM50"

normalyzerDE(jobName = jDEName, designPath = designP, dataPath = dataP, leastRepCount = 1, sampleCol="Sample_title", condCol = "AIMS_PAM50.Normal" , comparisons = c('1-0'), sigThres = 0.05, outputDir = outDir,batchCol = 'group')
```


```
# For HER2-enriched
dataP <- 'RNA-seq/GSE96058_gene_expression_Reduced.tsv'
designP <- 'DIA/DesignFilesDIA/design_aim2_clinical_matched_individualsubtypes.tsv'
outDir <- 'DE_analysis/DE_for_GSEA/'
jDEName <- "Her2 vs rest RNAseq AIMS PAM50"

normalyzerDE(jobName = jDEName, designPath = designP, dataPath = dataP, leastRepCount = 2, sampleCol="Sample_title", condCol = "AIMS_PAM50.Her2" , comparisons = c('1-0'), sigThres = 0.05, outputDir = outDir,batchCol = 'group')
```

## 5.2 Function


```
gseaMsigDB <- function(data,genecol='Genes',qvaluecutoff,padjustmethod='fdr',species='Homo sapiens',category='H',FCcolname='1-0_log2FoldChange',...){
  data <- read_tsv(data)
  FCcol <- data %>% dplyr::pull(FCcolname)
  data <- data %>% filter(!is.na(FCcol))
  genelist <- data %>% pull(FCcolname)
  names(genelist) <- data %>% dplyr::pull(genecol)
  genelist <- na.omit(genelist)
  genelist <- sort(x = genelist,decreasing = TRUE)
  
  term2gene <- msigdbr(species = species,category = category) %>% dplyr::select(gs_name,human_gene_symbol)
  
  gseResults <- clusterProfiler::GSEA(geneList = genelist,
                               pAdjustMethod = 'fdr',
                               eps = 0,
                               pvalueCutoff = qvaluecutoff,
                               TERM2GENE = term2gene,
                               by = 'fgsea')
  
  return(gseResults)
}
```

## 5.3 GSEA DIA-NN


```
#Luminal A
data <- 'DE_analysis/DE_for_GSEA/LumA vs rest Proteomics AIMS PAM50/LumA vs rest Proteomics AIMS PAM50_stats.tsv'
gseaMsigLumA <- gseaMsigDB(data = data,qvaluecutoff = 0.25,padjustmethod = 'fdr',species = 'Homo sapiens',category = 'H') %>% dplyr::mutate(PAM50='LumA')

#Luminal B
data <- 'DE_analysis/DE_for_GSEA/LumB vs rest Proteomics AIMS PAM50/LumB vs rest Proteomics AIMS PAM50_stats.tsv'
gseaMsigLumB <- gseaMsigDB(data = data,qvaluecutoff = 0.25,padjustmethod = 'fdr',species = 'Homo sapiens',category = 'H') %>% dplyr::mutate(PAM50='LumB')

#Basal
data <- 'DE_analysis/DE_for_GSEA/Basal vs rest Proteomics AIMS PAM50/Basal vs rest Proteomics AIMS PAM50_stats.tsv'
gseaMsigBasal <- gseaMsigDB(data = data,qvaluecutoff = 0.25,padjustmethod = 'fdr',species = 'Homo sapiens',category = 'H') %>% dplyr::mutate(PAM50='Basal')

#HER2-enriched
data <- 'DE_analysis/DE_for_GSEA/Her2 vs rest Proteomics AIMS PAM50/Her2 vs rest Proteomics AIMS PAM50_stats.tsv'
gseaMsigHer2 <- gseaMsigDB(data = data,qvaluecutoff = 0.25,padjustmethod = 'fdr',species = 'Homo sapiens',category = 'H') %>% dplyr::mutate(PAM50='Her2')

#Normal-like
data <- 'DE_analysis/DE_for_GSEA/Normal vs rest Proteomics AIMS PAM50/Normal vs rest Proteomics AIMS PAM50_stats.tsv'
gseaMsigNormal <- gseaMsigDB(data = data,qvaluecutoff = 0.25,padjustmethod = 'fdr',species = 'Homo sapiens',category = 'H') %>% dplyr::mutate(PAM50='Normal')
```

## 5.4 GSEA RNA-seq


```
#Luminal A 
data <- 'DE_analysis/DE_for_GSEA/LumA vs rest RNAseq AIMS PAM50/LumA vs rest RNAseq AIMS PAM50_stats.tsv'
gseaMsigLumARNA <- gseaMsigDB(data = data,genecol = 'Gene',qvaluecutoff = 0.25,padjustmethod = 'fdr',species = 'Homo sapiens',category = 'H') %>% dplyr::mutate(PAM50='LumA')

#Luminal B
data <- 'DE_analysis/DE_for_GSEA/LumB vs rest RNAseq AIMS PAM50/LumB vs rest RNAseq AIMS PAM50_stats.tsv'
gseaMsigLumBRNA <- gseaMsigDB(data = data,genecol = 'Gene',qvaluecutoff = 0.25,padjustmethod = 'fdr',species = 'Homo sapiens',category = 'H') %>% dplyr::mutate(PAM50='LumB')

#Basal 
data <- 'DE_analysis/DE_for_GSEA/Basal vs rest RNAseq AIMS PAM50/Basal vs rest RNAseq AIMS PAM50_stats.tsv'
gseaMsigBasalRNA <- gseaMsigDB(data = data,genecol = 'Gene',qvaluecutoff = 0.25,padjustmethod = 'fdr',species = 'Homo sapiens',category = 'H') %>% dplyr::mutate(PAM50='Basal')

#HER2-enriched
data <- 'DE_analysis/DE_for_GSEA/Her2 vs rest RNAseq AIMS PAM50/Her2 vs rest RNAseq AIMS PAM50_stats.tsv'
gseaMsigHer2RNA <- gseaMsigDB(data = data,genecol = 'Gene',qvaluecutoff = 0.25,padjustmethod = 'fdr',species = 'Homo sapiens',category = 'H') %>% dplyr::mutate(PAM50='Her2')

#Normal-like
data <- 'DE_analysis/DE_for_GSEA/Normal vs rest RNAseq AIMS PAM50/Normal vs rest RNAseq AIMS PAM50_stats.tsv'
gseaMsigNormalRNA <- gseaMsigDB(data = data,genecol = 'Gene',qvaluecutoff = 0.25,padjustmethod = 'fdr',species = 'Homo sapiens',category = 'H') %>% dplyr::mutate(PAM50='Normal')
```

## 5.5 Plotting


```
allResultsGSEAMsig <- list(Basal=gseaMsigBasal,Her2=gseaMsigHer2,LumA=gseaMsigLumA,LumB=gseaMsigLumB,Normal=gseaMsigNormal)
allResultsGSEAMsig <- map(allResultsGSEAMsig,~pluck(.x,'result')) %>% 
  map(.,as_tibble) %>% 
  bind_rows(.)

allResultsGSEAMsigRNA <- list(Basal=gseaMsigBasalRNA,Her2=gseaMsigHer2RNA,LumA=gseaMsigLumARNA,LumB=gseaMsigLumBRNA,Normal=gseaMsigNormalRNA)
allResultsGSEAMsigRNA <- map(allResultsGSEAMsigRNA,~pluck(.x,'result')) %>% 
  map(.,as_tibble) %>% 
  bind_rows(.)

resultsAll <- full_join(allResultsGSEAMsig,allResultsGSEAMsigRNA,by=c('ID','PAM50'),suffix=c('.DIA','.RNA'))
resultsAll$ID <- resultsAll$ID %>% 
  str_remove(string = .,pattern = 'HALLMARK\\_') %>% 
  str_replace_all(.,'\\_',' ')

plotSeparateSubtypes <- ggplot(data = resultsAll) + 
  geom_point(mapping = aes(x = NES.RNA,y = fct_reorder(.f = ID,.x = 1-(qvalues.RNA)),size = qvalues.RNA,fill = 'RNA'),shape = 21,alpha=0.85)

plotSeparateSubtypes <- plotSeparateSubtypes + 
  geom_point(mapping = aes(x = NES.DIA,y = ID,size = qvalues.DIA,fill = 'DIA'),shape = 21,alpha=0.85) + 
  theme_bw() + 
  labs( x = 'Normalized Enrichment Score',y='',title = 'GSEA - MSigDB Hallmarks') + 
  scale_radius("q-values", trans="log10", range=c(7, 3))+
  scale_fill_discrete('Experiment',type = c('#ca0020','#0571b0'))+
  facet_wrap(vars(PAM50),ncol = 5)

ggsave(filename = 'Figure1.png',plot = plotSeparateSubtypes,device = 'png',path = 'GSEA/',units = 'in',width = 9,height = 7,dpi = 600)
```

# 6 Decision Tree (Figures 2 and 3)

## 6.1 Differential Expression Analysis

In order to generate the decision tree model, a mixture of features
differentially expressed in different subtypes as well as in
clinicopathological parameters was used. Only proteomics data were used
to generate the model.


```
# Estrogen receptor positive vs Estrogen receptor negative
dataP <- 'DIA/DataDE/DIANN/DIA_DIANN_Protein_RRollup/DIA_DIANN_RRollup_Genes.tsv'
designP <- 'DIA/DesignFilesDIA/design_aim2_clinical_DecisionTree.tsv'
outDir <- 'DE_analysis/DE_for_decisiontree/'
jDEName <- "ERp vs ERn"

normalyzerDE(jobName = jDEName,designPath = designP,dataPath = dataP,outputDir = outDir,sampleCol = 'Position',condCol = 'ER_status',comparisons = c('Pos-Neg'),leastRepCount = 3)
```


```
# Lymphnode positive vs Lymphnode negative
dataP <- 'DIA/DataDE/DIANN/DIA_DIANN_Protein_RRollup/DIA_DIANN_RRollup_Genes.tsv'
designP <- 'DIA/DesignFilesDIA/design_aim2_clinical_DecisionTree.tsv'
outDir <- 'DE_analysis/DE_for_decisiontree/'
jDEName <- "NodePositive vs NodeNegative"

normalyzerDE(jobName = jDEName,designPath = designP,dataPath = dataP,outputDir = outDir,sampleCol = 'Position',condCol = 'LN_status',comparisons = c('NodePositive-NodeNegative'),leastRepCount = 3)
```


```
# HER2 receptor positive vs HER2 receptor negative
dataP <- 'DIA/DataDE/DIANN/DIA_DIANN_Protein_RRollup/DIA_DIANN_RRollup_Genes.tsv'
designP <- 'DIA/DesignFilesDIA/design_aim2_clinical_DecisionTree.tsv'
outDir <- 'DE_analysis/DE_for_decisiontree/'
jDEName <- "Her2p vs Her2n"

normalyzerDE(jobName = jDEName,designPath = designP,dataPath = dataP,outputDir = outDir,sampleCol = 'Position',condCol = 'HER2_status',comparisons = c('Pos-Neg'),leastRepCount = 3)
```


```
# Tumor Grade 3 vs Tumor Grade 1
dataP <- 'DIA/DataDE/DIANN/DIA_DIANN_Protein_RRollup/DIA_DIANN_RRollup_Genes.tsv'
designP <- 'DIA/DesignFilesDIA/design_aim2_clinical_DecisionTree.tsv'
outDir <- 'DE_analysis/DE_for_decisiontree/'
jDEName <- "Grade3 vs Grade1"

normalyzerDE(jobName = jDEName,designPath = designP,dataPath = dataP,outputDir = outDir,sampleCol = 'Position',condCol = 'Grade',comparisons = c('1-3'),leastRepCount = 3)
```


```
# Basal vs Luminal A
dataP <- 'DIA/DataDE/DIANN/DIA_DIANN_Protein_RRollup/DIA_DIANN_RRollup_Genes.tsv'
designP <- 'DIA/DesignFilesDIA/design_aim2_clinical_DecisionTree.tsv'
outDir <- 'DE_analysis/DE_for_decisiontree/'
jDEName <- "Basal vs LumA"

normalyzerDE(jobName = jDEName,designPath = designP,dataPath = dataP,outputDir = outDir,sampleCol = 'Position',condCol = 'AIMS_PAM50',comparisons = c('Basal-LumA'),leastRepCount = 3)
```


```
# Luminal A vs Luminal B
dataP <- 'DIA/DataDE/DIANN/DIA_DIANN_Protein_RRollup/DIA_DIANN_RRollup_Genes.tsv'
designP <- 'DIA/DesignFilesDIA/design_aim2_clinical_DecisionTree.tsv'
outDir <- 'DE_analysis/DE_for_decisiontree/'
jDEName <- "LumA vs LumB"

normalyzerDE(jobName = jDEName,designPath = designP,dataPath = dataP,outputDir = outDir,sampleCol = 'Position',condCol = 'AIMS_PAM50',comparisons = c('LumA-LumB'),leastRepCount = 3)
```


```
# Luminal B vs HER2-enriched
dataP <- 'DIA/DataDE/DIANN/DIA_DIANN_Protein_RRollup/DIA_DIANN_RRollup_Genes.tsv'
designP <- 'DIA/DesignFilesDIA/design_aim2_clinical_DecisionTree.tsv'
outDir <- 'DE_analysis/DE_for_decisiontree/'
jDEName <- "LumB vs Her2"

normalyzerDE(jobName = jDEName,designPath = designP,dataPath = dataP,outputDir = outDir,sampleCol = 'Position',condCol = 'AIMS_PAM50',comparisons = c('LumB-Her2'),leastRepCount = 3)
```


```
# Basal vs HER2-enriched
dataP <- 'DIA/DataDE/DIANN/DIA_DIANN_Protein_RRollup/DIA_DIANN_RRollup_Genes.tsv'
designP <- 'DIA/DesignFilesDIA/design_aim2_clinical_DecisionTree.tsv'
outDir <- 'DE_analysis/DE_for_decisiontree/'
jDEName <- "Basal vs Her2"

normalyzerDE(jobName = jDEName,designPath = designP,dataPath = dataP,outputDir = outDir,sampleCol = 'Position',condCol = 'AIMS_PAM50',comparisons = c('Basal-Her2'),leastRepCount = 3)
```


```
# HER2-enriched vs Luminal A
dataP <- 'DIA/DataDE/DIANN/DIA_DIANN_Protein_RRollup/DIA_DIANN_RRollup_Genes.tsv'
designP <- 'DIA/DesignFilesDIA/design_aim2_clinical_DecisionTree.tsv'
outDir <- 'DE_analysis/DE_for_decisiontree/'
jDEName <- "Her2 vs LumA"

normalyzerDE(jobName = jDEName,designPath = designP,dataPath = dataP,outputDir = outDir,sampleCol = 'Position',condCol = 'AIMS_PAM50',comparisons = c('Her2-LumA'),leastRepCount = 3)
```

## 6.2 Data Import and preparing training and test sets

The input data for the decision tree model is a list of
differentially expressed proteins across different conditions after
filtering based on adjusted p-value of 0.01 and absolute log2FoldChange
of 2 and collapsing the data into unique Protein Group IDs. The DIA-NN
expression data is then filtered based on these IDs and the normalized
abundance values are used for the model.


```
# Move all .tsv files to a common directory
cp -Rv ~/SCANB_PUBLICATION/DE_analysis/DE_for_decisiontree/*/*.tsv ~/SCANB_PUBLICATION/DE_analysis/DE_for_decisiontree
```


```
# List of differentially abundant proteins
DEFiles <- list.files(pattern = '*.tsv',path = 'DE_analysis/DE_for_decisiontree/')
DEData <- map(.x = DEFiles,.f = ~read_tsv(file = paste0('DE_analysis/DE_for_decisiontree/',.x)))
names(DEData) <- DEFiles

DEDataFilter <- map(.x = DEData,.f = ~filter(.data = .x,.x[str_detect(string = colnames(.x),pattern = 'AdjPVal')]<0.01)) %>% 
  map(.x = .,.f = ~filter(.x,abs(.x[str_detect(string = colnames(.x),pattern = 'log2FoldChange')])>=2)) %>% 
  map(.x = .,.f = ~pull(.x,Protein)) %>% 
  unlist %>% 
  unique
```


```
# Data import
data <- 'DIA/DataDE/DIANN/DIA_DIANN_Protein_RRollup/DIA_DIANN_RRollup_Genes.tsv' %>% 
  read_tsv
design <-  'DIA/DesignFilesDIA/design_aim2_clinical_DecisionTree.tsv' %>% 
  read_tsv
```


```
# Setting up the input data frame
dataComplete <- data %>% 
  dplyr::select(Protein,design$Position) %>%
  filter(Protein %in% DEDataFilter)
protNames <- dataComplete$Protein

dataComplete <- dataComplete %>% 
  dplyr::select(design$Position) %>% 
  t

colnames(dataComplete) <- protNames

outVar <- design$AIMS_PAM50 %>% as.factor
dataComplete <- data.frame(dataComplete,OutVar=outVar,Position=rownames(dataComplete)) %>% 
  filter(!is.na(OutVar))
```


```
# Splitting the data in 70% for training and 30% for testing based on the intrinsic subtypes
trainSet <- dataComplete %>% group_by(OutVar) %>% sample_frac(0.7)

diffRows <- setdiff(design$Position,trainSet$Position)
testSet <- dataComplete[diffRows,]

dataCompleteClean <- dataComplete %>% 
  dplyr::select(-Position)
trainSetClean <- trainSet %>% 
  dplyr::select(-Position)
testSetClean <- testSet %>% 
  dplyr::select(-Position)
```

## 6.3 Model Training and Plotting

### 6.3.1 Training


```
set.seed(4321)
protTreeModel <- train(OutVar ~ ., data = trainSetClean, method = "ctree",
    trControl = trainControl(method = "boot"),na.action = na.pass)
plot(protTreeModel$finalModel) #Figure 2
```

### 6.3.2 Plotting


```
png(filename = 'decision_trees/Figure2.png',width = 19,height = 9,units = 'in',res = 600)
plot(protTreeModel$finalModel)
dev.off()
```

## 6.4 Model Testing


```
predScanb <- predict(protTreeModel,testSetClean,na.action = na.pass)
confusionMatrix(table(testSet$OutVar,predScanb))
save.image(file = 'decision_trees/modelData.RData',safe = TRUE)
```

## 6.5 Decision Tree from proteins found in Bouchal P., et. al., (2019)


```
load(file = 'decision_trees/modelData.RData')
ERBB2 <- 'P04626'
INPP4B <- 'O15327'
CDK1 <- 'P06493'
trainSwath <- trainSetClean %>% 
  dplyr::select(ERBB2,INPP4B,CDK1,OutVar)
testSwath <- testSetClean %>% 
  dplyr::select(ERBB2,INPP4B,CDK1,OutVar)

set.seed(4321)
fitSwath <- train(OutVar ~ ., data = trainSwath, method = "ctree",
              trControl = trainControl(method = "boot"),na.action = na.pass)
plot(fitSwath$finalModel) #Figure 3
```

# 7 Unsupervised Hierarchical Clustering (FIGURE 4 AND SUPPLEMENTARY FIGURES S1-2)

## 7.1 Loading Data


```
# Proteomics Data
proteinData <- 'DIA/DataDE/DIANN/DIA_DIANN_Protein_RRollup/DIA_DIANN_RRollup_Genes.tsv' %>% 
  read_tsv %>% 
  drop_na
design <- 'DIA/DesignFilesDIA/design_aim2_clinical_matched_individualsubtypes.tsv' %>% 
  read_tsv
```


```
# Transcriptomics Data
rnaData <- 'RNA-seq/GSE96058_gene_expression_Reduced.tsv' %>% 
  read_tsv %>% 
  drop_na
```

## 7.2 Clustering after low variance filtering

### 7.2.1 Matching samples


```
proteinDataMatched <- proteinData %>% 
  dplyr::select(Genes,design$Position)
rnaDataMatched <- rnaData %>% 
  dplyr::select(Gene,design$Sample_title)
```

### 7.2.2 Function to remove low variance features


```
medianFiltering <- function(data,design,sampleCol,annotation,...){
  varianceNA <- function(data) var(data,na.rm=TRUE)
  
  rowName <- pull(data,annotation)
  varianceData <- data %>% 
    dplyr::select(.,pull(design,sampleCol)) %>% 
    t %>% 
    as_tibble %>% 
    map_dfc(.,varianceNA) %>% 
    t
  
  dataVar <- data %>% 
    mutate(.,variance=varianceData,Protein=rowName)
  
  lowVarData <- dataVar %>% 
    filter(variance > median(variance,na.rm = T))
  
  return(lowVarData)
}
```

### 7.2.3 Clustering with annotations

#### 7.2.3.1 DIA-NN


```
proteinDataFiltered <- medianFiltering(data = proteinDataMatched,design = design,sampleCol = 'Position',annotation = 'Genes')

proteinDataReduced <- proteinDataFiltered %>% 
  dplyr::select(design$Position)

# Calculation of Z-Scores
calZScore <- function(x) (x-mean(x))/sd(x)

proteindataNorm <- t(apply(proteinDataReduced,1,calZScore))
colnames(proteindataNorm) <- NULL

annotationColumn <- HeatmapAnnotation(PAM50=anno_simple(x = design$PAM50,
                                                        col = c('AIMS_PAM50.Basal'='#e31a1c','AIMS_PAM50.Her2'='#fb9a99','AIMS_PAM50.LumA'='#1f78b4','AIMS_PAM50.LumB'='#a6cee3','AIMS_PAM50.Normal'='#33a02c'),
                                                        na_col = '#808080',
                                                        gp = gpar(title='PAM50')),
                                      NodeStatus=anno_simple(x = design$`LN status`,
                                                             col = c('NodePositive'='#000000','NodeNegative'='#ffffff'),
                                                             na_col = '#808080',
                                                             gp = gpar(title='NodeStatus')),
                                      HER2=anno_simple(x = design$`HER2 status`,
                                                       col = c('Pos'='#000000','Neg'='#ffffff'),
                                                       na_col = '#808080',
                                                       gp = gpar(title='HER2')),
                                      ER=anno_simple(x = design$`ER status`,
                                                     col = c('Pos'='#000000','Neg'='#ffffff'),
                                                     na_col = '#808080',
                                                     gp = gpar(title='ER')),
                                      PR=anno_simple(x = design$`PR status`,
                                                      col = c('Pos'='#000000','Neg'='#ffffff'),
                                                      na_col = '#808080',
                                                      gp = gpar(title='PR')))

legendPAM50 <- Legend(labels = c('Basal','Her2','LumA','LumB','Normal'),
                      title = 'PAM50 subtype',
                      border = 'black',
                      legend_gp = gpar(fill=c('Basal'='#e31a1c','Her2'='#fb9a99','LumA'='#1f78b4','LumB'='#a6cee3','Normal'='#33a02c')))
legendHer2 <- Legend(labels = c('Positive','Negative','NA'),
                     title = 'HER2 status',
                     border = 'black',
                     legend_gp = gpar(fill=c('Positive'='#000000','Negative'='#ffffff','NA'='#808080')))
legendNodeStatus <- Legend(labels = c('Positive','Negative'),
                           title = 'Node status',
                           border = 'black',
                           legend_gp = gpar(fill=c('Positive'='#000000','Negative'='#ffffff')))
legendER <- Legend(labels = c('Positive','Negative'),
                   title = 'ER status',
                   border = 'black',
                   legend_gp = gpar(fill=c('Positive'='#000000','Negative'='#ffffff')))
legendPR <- Legend(labels = c('Positive','Negative'),
                    title = 'PR status',
                    border = 'black',
                    legend_gp = gpar(fill=c('Positive'='#000000','Negative'='#ffffff')))

allLegends <- packLegend(legendPAM50,legendHer2,legendNodeStatus,legendER,legendPR)
proteinHeatmap <- Heatmap(matrix = proteindataNorm,top_annotation = annotationColumn, name = 'Protein intensity',heatmap_legend_param = list(legend_direction = 'horizontal'))

proteinHeatmapDraw <- grid.grabExpr(draw(proteinHeatmap,annotation_legend_list = allLegends, heatmap_legend_side = 'bottom', annotation_legend_side = 'right', legend_grouping = 'original')) %>% as.ggplot

ggsave2(filename = 'supplementary_figureS1A.pdf',plot = proteinHeatmapDraw,device = 'pdf',path = 'clustering/',dpi = 600,width = 10,height = 10,units = 'in')
```

#### 7.2.3.2 RNA-seq


```
rnaDataFiltered <- medianFiltering(data = rnaDataMatched,design = design,sampleCol = 'Sample_title',annotation = 'Gene')

rnaDataReduced <- rnaDataFiltered %>% 
  dplyr::select(design$Sample_title)

rnaDataNorm <- t(apply(rnaDataReduced,1,calZScore))
colnames(rnaDataNorm) <- NULL

rnaHeatmap <- Heatmap(matrix = rnaDataNorm,top_annotation = annotationColumn, name = 'Gene expression',heatmap_legend_param = list(legend_direction = 'horizontal'))
rnaHeatmapDraw <- grid.grabExpr(draw(rnaHeatmap,annotation_legend_list = allLegends, heatmap_legend_side = 'bottom', annotation_legend_side = 'right', legend_grouping = 'original')) %>% as.ggplot

ggsave2(filename = 'supplementary_figureS1B.pdf',plot = rnaHeatmapDraw,device = 'pdf',path = 'clustering/',dpi = 600, width = 10,height = 10,units = 'in')
```

#### 7.2.3.3 Joint Plots


```
htProt <- proteinHeatmapDraw
htRNA <- grid.grabExpr(draw(rnaHeatmap,heatmap_legend_side = 'bottom')) %>% as.ggplot
jointPlotVar <- plot_grid(htProt,htRNA,labels = LETTERS[1:2])
ggsave2(filename = 'supplementary_figureS1.pdf',plot = jointPlotVar,device = 'pdf',path = 'clustering/',dpi = 600,width = 15,height = 10,units = 'in')
```

## 7.3 Clustering with features from Decision Tree

### 7.3.1 List of differentially abundant features


```
DEFiles <- list.files(pattern = '*.tsv',path = 'DE_analysis/DE_for_decisiontree/')
DEData <- map(.x = DEFiles,.f = ~read_tsv(file = paste0('DE_analysis/DE_for_decisiontree/',.x)))
names(DEData) <- DEFiles

DEDataFilter <- map(.x = DEData,.f = ~filter(.data = .x,.x[str_detect(string = colnames(.x),pattern = 'AdjPVal')]<0.01)) %>% 
  map(.x = .,.f = ~filter(.x,abs(.x[str_detect(string = colnames(.x),pattern = 'log2FoldChange')])>=2)) %>% 
  map(.x = .,.f = ~pull(.x,Protein)) %>% 
  unlist %>% 
  unique
```

### 7.3.2 Filter Data


```
protDataTree <- proteinData %>%
  filter(Protein %in% DEDataFilter) %>% 
  drop_na

geneNames <- protDataTree$Genes

rnaDataTree <- rnaData %>% 
  filter(Gene %in% geneNames) %>% 
  drop_na
```

### 7.3.3 Matching Data


```
protTreeMatch <- protDataTree %>% 
  dplyr::select(Genes,design$Position)
rnaTreeMatch <- rnaDataTree %>% 
  dplyr::select(Gene,design$Sample_title)
```

### 7.3.4 Clustering

#### 7.3.4.1 DIA-NN


```
protTreeReduced <- protTreeMatch %>% 
  dplyr::select(design$Position)

# Calculation of Z-Scores
calZScore <- function(x) (x-mean(x))/sd(x)

protTreeNorm <- t(apply(protTreeReduced,1,calZScore))
colnames(protTreeNorm) <- NULL
rownames(protTreeNorm) <- protTreeMatch$Genes

annotationColumn <- HeatmapAnnotation(PAM50=anno_simple(x = design$PAM50,
                                                        col = c('AIMS_PAM50.Basal'='#e31a1c','AIMS_PAM50.Her2'='#fb9a99','AIMS_PAM50.LumA'='#1f78b4','AIMS_PAM50.LumB'='#a6cee3','AIMS_PAM50.Normal'='#33a02c'),
                                                        na_col = '#808080',
                                                        gp = gpar(title='PAM50')),
                                      NodeStatus=anno_simple(x = design$`LN status`,
                                                             col = c('NodePositive'='#000000','NodeNegative'='#ffffff'),
                                                             na_col = '#808080',
                                                             gp = gpar(title='NodeStatus')),
                                      HER2=anno_simple(x = design$`HER2 status`,
                                                       col = c('Pos'='#000000','Neg'='#ffffff'),
                                                       na_col = '#808080',
                                                       gp = gpar(title='HER2')),
                                      ER=anno_simple(x = design$`ER status`,
                                                     col = c('Pos'='#000000','Neg'='#ffffff'),
                                                     na_col = '#808080',
                                                     gp = gpar(title='ER')),
                                      PR=anno_simple(x = design$`PR status`,
                                                      col = c('Pos'='#000000','Neg'='#ffffff'),
                                                      na_col = '#808080',
                                                      gp = gpar(title='PR')))

legendPAM50 <- Legend(labels = c('Basal','Her2','LumA','LumB','Normal'),
                      title = 'PAM50 subtype',
                      border = 'black',
                      legend_gp = gpar(fill=c('Basal'='#e31a1c','Her2'='#fb9a99','LumA'='#1f78b4','LumB'='#a6cee3','Normal'='#33a02c')))
legendHer2 <- Legend(labels = c('Positive','Negative','NA'),
                     title = 'HER2 status',
                     border = 'black',
                     legend_gp = gpar(fill=c('Positive'='#000000','Negative'='#ffffff','NA'='#808080')))
legendNodeStatus <- Legend(labels = c('Positive','Negative'),
                           title = 'Node status',
                           border = 'black',
                           legend_gp = gpar(fill=c('Positive'='#000000','Negative'='#ffffff')))
legendER <- Legend(labels = c('Positive','Negative'),
                   title = 'ER status',
                   border = 'black',
                   legend_gp = gpar(fill=c('Positive'='#000000','Negative'='#ffffff')))
legendPR <- Legend(labels = c('Positive','Negative'),
                    title = 'PR status',
                    border = 'black',
                    legend_gp = gpar(fill=c('Positive'='#000000','Negative'='#ffffff')))

allLegends <- packLegend(legendPAM50,legendHer2,legendNodeStatus,legendER,legendPR)

protTreeHeatmap <- Heatmap(matrix = protTreeNorm,top_annotation = annotationColumn,name = 'Protein intensity', heatmap_legend_param = list(legend_direction = 'horizontal'))

protTreeHeatmapDraw <- grid.grabExpr(draw(protTreeHeatmap,annotation_legend_list = allLegends, heatmap_legend_side = 'bottom', annotation_legend_side = 'right', legend_grouping = 'original')) %>% as.ggplot

ggsave2(filename = 'figure4A.pdf',plot = protTreeHeatmapDraw,device = 'pdf',path = 'clustering/',dpi = 600,width = 10,height = 10,units = 'in')
```

#### 7.3.4.2 RNA-seq


```
rnaTreeReduced <- rnaTreeMatch %>% 
  dplyr::select(design$Sample_title)

rnaTreeNorm <- t(apply(rnaTreeReduced,1,calZScore))
colnames(rnaTreeNorm) <- NULL
rownames(rnaTreeNorm) <- rnaTreeMatch$Gene

rnaTreeHeatmap <- Heatmap(matrix = rnaTreeNorm,top_annotation = annotationColumn, name = 'Gene expression', heatmap_legend_param = list(legend_direction = 'horizontal'))

rnaTreeHeatmapDraw <- grid.grabExpr(draw(rnaTreeHeatmap,annotation_legend_list = allLegends, heatmap_legend_side = 'bottom', annotation_legend_side = 'right', legend_grouping = 'original')) %>% as.ggplot

ggsave2(filename = 'figure4B.pdf',plot = rnaTreeHeatmapDraw,device = 'pdf',path = 'clustering/',dpi = 600,width = 10,height = 10,units = 'in')
```

#### 7.3.4.3 Joint Plots


```
htProtTree <- protTreeHeatmapDraw
htRNATree <- grid.grabExpr(draw(rnaTreeHeatmap,heatmap_legend_side = 'bottom')) %>% as.ggplot
jointPlotTree <- plot_grid(htProtTree,htRNATree,labels = LETTERS[1:2])
ggsave2(filename = 'figure4.pdf',plot = jointPlotTree,device = 'pdf',path = 'clustering/',dpi = 600,width = 15,height = 10,units = 'in')
```

## 7.4 Clustering with features from GSEA

### 7.4.1 List of features differentially expressed

#### 7.4.1.1 Proteomics


```
mkdir ~/SCANB_PUBLICATION/clustering/proteomics_gsea_clustering
mkdir ~/SCANB_PUBLICATION/clustering/transcriptomics_gsea_clustering

cp -Rv ~/SCANB_PUBLICATION/DE_analysis/DE_for_GSEA/*Proteomics*/*.tsv ~/SCANB_PUBLICATION/clustering/proteomics_gsea_clustering
cp -Rv ~/SCANB_PUBLICATION/DE_analysis/DE_for_GSEA/*RNA*/*.tsv ~/SCANB_PUBLICATION/clustering/transcriptomics_gsea_clustering
```


```
DEFilesProt <- list.files(pattern = '*.tsv',path = 'clustering/proteomics_gsea_clustering/')
DEDataProt <- map(.x = DEFilesProt,.f = ~read_tsv(file = paste0('clustering/proteomics_gsea_clustering/',.x)))
names(DEDataProt) <- DEFilesProt

DEDataFilterProt <- map(.x = DEDataProt,.f = ~filter(.x,.x[str_detect(string = colnames(.x),pattern = 'AdjPVal')]<0.1)) %>% 
  map(.x = .,.f = ~filter(.x,abs(.x[str_detect(string = colnames(.x),pattern = 'log2FoldChange')])>=1.5)) %>% 
  map(.x = .,.f = ~pull(.x,Protein)) %>% 
  unlist %>% 
  unique
```

#### 7.4.1.2 Transcriptomics


```
DEFilesRNA <- list.files(pattern = '*.tsv',path = 'clustering/transcriptomics_gsea_clustering/')
DEDataRNA <- map(.x = DEFilesRNA,.f = ~read_tsv(file = paste0('clustering/transcriptomics_gsea_clustering/',.x)))
names(DEDataRNA) <- DEFilesRNA

DEDataFilterRNA <- map(.x = DEDataRNA,.f = ~filter(.x,.x[str_detect(string = colnames(.x),pattern = 'AdjPVal')]<0.1)) %>% 
  map(.x = .,.f = ~filter(.x,abs(.x[str_detect(string = colnames(.x),pattern = 'log2FoldChange')])>=1.5)) %>% 
  map(.x = .,.f = ~pull(.x,Gene)) %>% 
  unlist %>% 
  unique
```

### 7.4.2 Filter Data

#### 7.4.2.1 Proteomics


```
proteinData <- 'DIA/DataDE/DIANN/DIA_DIANN_Protein_RRollup/DIA_DIANN_RRollup_Genes.tsv' %>% 
  read_tsv
proteinDataFiltered <- proteinData %>% 
  filter(Protein %in% DEDataFilterProt) %>% 
  drop_na
```

#### 7.4.2.2 Transcriptomics


```
rnaData <- 'RNA-seq/GSE96058_gene_expression_Reduced.tsv' %>% 
  read_tsv
rnaDataFiltered <- rnaData %>% 
  filter(Gene %in% DEDataFilterRNA) %>% 
  drop_na
```

### 7.4.3 Matching Data

#### 7.4.3.1 Proteomics


```
protCommonSamples <- proteinDataFiltered %>% 
  dplyr::select(Genes,design$Position)
```

#### 7.4.3.2 Transcriptomics


```
rnaCommonSamples <- rnaDataFiltered %>% 
  dplyr::select(Gene,design$Sample_title)
```

### 7.4.4 Clustering

#### 7.4.4.1 DIA-NN


```
proteinDataReduced <- protCommonSamples %>% 
  dplyr::select(design$Position)

# Calculation of Z-Scores
calZScore <- function(x) (x-mean(x))/sd(x)

proteindataNorm <- t(apply(proteinDataReduced,1,calZScore))
colnames(proteindataNorm) <- NULL

annotationColumn <- HeatmapAnnotation(PAM50=anno_simple(x = design$PAM50,
                                                        col = c('AIMS_PAM50.Basal'='#e31a1c','AIMS_PAM50.Her2'='#fb9a99','AIMS_PAM50.LumA'='#1f78b4','AIMS_PAM50.LumB'='#a6cee3','AIMS_PAM50.Normal'='#33a02c'),
                                                        na_col = '#808080',
                                                        gp = gpar(title='PAM50')),
                                      NodeStatus=anno_simple(x = design$`LN status`,
                                                             col = c('NodePositive'='#000000','NodeNegative'='#ffffff'),
                                                             na_col = '#808080',
                                                             gp = gpar(title='NodeStatus')),
                                      HER2=anno_simple(x = design$`HER2 status`,
                                                       col = c('Pos'='#000000','Neg'='#ffffff'),
                                                       na_col = '#808080',
                                                       gp = gpar(title='HER2')),
                                      ER=anno_simple(x = design$`ER status`,
                                                     col = c('Pos'='#000000','Neg'='#ffffff'),
                                                     na_col = '#808080',
                                                     gp = gpar(title='ER')),
                                      PR=anno_simple(x = design$`PR status`,
                                                      col = c('Pos'='#000000','Neg'='#ffffff'),
                                                      na_col = '#808080',
                                                      gp = gpar(title='PR')))

legendPAM50 <- Legend(labels = c('Basal','Her2','LumA','LumB','Normal'),
                      title = 'PAM50 subtype',
                      border = 'black',
                      legend_gp = gpar(fill=c('Basal'='#e31a1c','Her2'='#fb9a99','LumA'='#1f78b4','LumB'='#a6cee3','Normal'='#33a02c')))
legendHer2 <- Legend(labels = c('Positive','Negative','NA'),
                     title = 'HER2 status',
                     border = 'black',
                     legend_gp = gpar(fill=c('Positive'='#000000','Negative'='#ffffff','NA'='#808080')))
legendNodeStatus <- Legend(labels = c('Positive','Negative'),
                           title = 'Node status',
                           border = 'black',
                           legend_gp = gpar(fill=c('Positive'='#000000','Negative'='#ffffff')))
legendER <- Legend(labels = c('Positive','Negative'),
                   title = 'ER status',
                   border = 'black',
                   legend_gp = gpar(fill=c('Positive'='#000000','Negative'='#ffffff')))
legendPR <- Legend(labels = c('Positive','Negative'),
                    title = 'PR status',
                    border = 'black',
                    legend_gp = gpar(fill=c('Positive'='#000000','Negative'='#ffffff')))

allLegends <- packLegend(legendPAM50,legendHer2,legendNodeStatus,legendER,legendPR)

proteinHeatmapGSEA <- Heatmap(matrix = proteindataNorm,top_annotation = annotationColumn, name = 'Protein intensity', heatmap_legend_param = list(legend_direction = 'horizontal'))

proteinHeatmapGSEADraw <- grid.grabExpr(draw(proteinHeatmapGSEA,annotation_legend_list = allLegends, heatmap_legend_side = 'bottom', annotation_legend_side = 'right', legend_grouping = 'original')) %>% as.ggplot

ggsave2(filename = 'supplementary_figureS2A.pdf',plot = proteinHeatmapGSEADraw,device = 'pdf',path = 'clustering/',dpi = 600,width = 10,height = 10,units = 'in')
```

#### 7.4.4.2 RNA-seq


```
rnaReduced <- rnaCommonSamples %>% 
  dplyr::select(design$Sample_title)

rnaNorm <- t(apply(rnaReduced,1,calZScore))
colnames(rnaNorm) <- NULL

rnaHeatmapGSEA <- Heatmap(matrix = rnaNorm,top_annotation = annotationColumn,name = 'Gene expression', heatmap_legend_param = list(legend_direction = 'horizontal'))

rnaHeatmapGSEADraw <- grid.grabExpr(draw(rnaHeatmapGSEA,annotation_legend_list = allLegends, heatmap_legend_side = 'bottom', annotation_legend_side = 'right', legend_grouping = 'original')) %>% as.ggplot

ggsave2(filename = 'supplementary_figureS2B.pdf',plot = rnaHeatmapGSEADraw,device = 'pdf',path = 'clustering/',dpi = 600,width = 10,height = 10,units = 'in')
```

#### 7.4.4.3 Joint Plots


```
htProtGSEA <- proteinHeatmapGSEADraw
htRNAGSEA <- grid.grabExpr(draw(rnaHeatmapGSEA,heatmap_legend_side = 'bottom')) %>% as.ggplot
jointPlotGSEA <- plot_grid(htProtGSEA,htRNAGSEA,labels = LETTERS[1:2])
ggsave2(filename = 'supplementary_figureS2.pdf',plot = jointPlotGSEA,device = 'pdf',path = 'clustering/',dpi = 600,width = 15,height = 10,units = 'in')
```

LS0tCnRpdGxlOiAiU0NBTkIgRGF0YSBBbmFseXNpcyIKYXV0aG9yOiAiU2VyZ2lvIE1vc3F1aW0gSnVuaW9yIgpkYXRlOiAnMjAyMi0wOS0yNicKb3V0cHV0OgogIGh0bWxfbm90ZWJvb2s6CiAgICB0b2M6IFRSVUUKICAgIHRvY19mbG9hdDogVFJVRQogICAgbnVtYmVyX3NlY3Rpb25zOiBUUlVFCi0tLQoKYGBge3Igc2V0dXAsIGluY2x1ZGU9RkFMU0V9CmtuaXRyOjpvcHRzX2NodW5rJHNldChlY2hvID0gVFJVRSkKYGBgCgojIFJlcXVpcmVkIFBhY2thZ2VzCmBgYHtyfQojIFByZS1wcm9jZXNzaW5nCmxpYnJhcnkodGlkeXZlcnNlKQpsaWJyYXJ5KE5vcm1hbHl6ZXJERSkKbGlicmFyeShiaW9tYVJ0KQoKIyBDb3JyZWxhdGlvbnMKbGlicmFyeShnZGF0YSkKCiMgR2VuZSBTZXQgRW5yaWNobWVudCBBbmFseXNpcwpsaWJyYXJ5KGNsdXN0ZXJQcm9maWxlcikKbGlicmFyeShtc2lnZGJyKQpsaWJyYXJ5KG9yZy5Icy5lZy5kYikKbGlicmFyeShnZ3Bsb3QyKQoKIyBEZWNpc2lvbiBUcmVlcwpsaWJyYXJ5KGNhcmV0KQoKIyBVbnN1cGVydmlzZWQgSGllcmFyY2hpY2FsIENsdXN0ZXJpbmcKbGlicmFyeShDb21wbGV4SGVhdG1hcCkKbGlicmFyeShncmlkKQpsaWJyYXJ5KGdncGxvdGlmeSkKbGlicmFyeShjb3dwbG90KQpgYGAKIyBHZW5lcmF0ZSBEZXNpZ24gRmlsZXMgdXNlZCBpbiBkYXRhIGFuYWx5c2lzCiAgCiMjIEREQSBhbmQgRElBIHByZS1wcm9jZXNzaW5nCiAgVGhlIG92ZXJhbGxfZGVzaWduIGZpbGUgaXMgdGhlIFN1cHBsZW1lbnRhcnkgVGFibGUgUzEgZnJvbQkiVmFsbG9uLUNocmlzdGVyc3NvbiwgSi47IEhha2tpbmVuLCBKLjsgSGVnYXJkdCwgQy47IFNhYWwsIEwuSC47IExhcnNzb24sIEMuOyBFaGluZ2VyLCBBLjsgTGluZG1hbiwgSC47IE9sb2Zzc29uLCBILjsgU2pvYmxvbSwgVC47IFdhcm5iZXJnLCBGLjsgZXQgYWwuIENyb3NzIGNvbXBhcmlzb24gYW5kIHByb2dub3N0aWMgYXNzZXNzbWVudCBvZiBicmVhc3QgY2FuY2VyIG11bHRpZ2VuZSBzaWduYXR1cmVzIGluIGEgbGFyZ2UgcG9wdWxhdGlvbi1iYXNlZCBjb250ZW1wb3JhcnkgY2xpbmljYWwgc2VyaWVzLiBTY2llbnRpZmljIHJlcG9ydHMgMjAxOSwgOSwgMTIxODQsIGRvaToxMC4xMDM4L3M0MTU5OC0wMTktNDg1NzAteC4iIFRoZSBmaWxlIHdhcyBmdXJ0aGVyIGZpbHRlcmVkIHRvIG9ubHkgaW5jbHVkZSB0aGUgc2FtcGxlcyBpbiB0aGUgcHJlc2VudCBzdHVkeS4KYGBge3J9CmJhc2VEZXNpZ24gPC0gcmVhZF90c3YoJ292ZXJhbGxfZGVzaWduLnRzdicpCiMgRElBCmRlc2lnbkRJQSA8LSBjbGluaWNhbEFsbCAlPiUgCiAgZHBseXI6OnNlbGVjdCgtRERBKSAlPiUgCiAgZmlsdGVyKCFpcy5uYShESUEpKQp3cml0ZV90c3YoeCA9IGRlc2lnbkRJQSxmaWxlID0gJ0RJQS9EZXNpZ25GaWxlc0RJQS9kZXNpZ25fYWltMl9jbGluaWNhbC50c3YnKQojIEREQQpkZXNpZ25EREEgPC0gY2xpbmljYWxBbGwgJT4lIAogIGRwbHlyOjpzZWxlY3QoLURJQSkgJT4lIAogIGZpbHRlcighaXMubmEoRERBKSkKd3JpdGVfdHN2KHggPSBkZXNpZ25EREEsZmlsZSA9ICdEREEvRGVzaWduRmlsZXNEREEvZGVzaWduX2FpbTJfY2xpbmljYWwudHN2JykKYGBgCgojIyBSTkEtc2VxIHByZS1wcm9jZXNzaW5nCgogIFRoZSAic2NhbmJfZ2VuZV9kZXNpZ24udHh0IiBmaWxlIHdhcyBjcmVhdGVkIGJ5IGRvd25sb2FkaW5nIHRoZSBzZXJpZXMgbWF0cml4IGZpbGUgYXZhaWxhYmxlIG9uIEdlbmUgRXhwcmVzc2lvbiBPbW5pYnVzIElEIEdTRTk2MDU4IGFuZCBmdXJ0aGVyIHJlbW92aW5nIHRoZSBoZWFkZXIgYW5kIHRyYW5zcG9zaW5nIGl0LCBzbyB0aGF0IHRoZSB2YXJpYWJsZXMgd291bGQgYWxpZ24gd2l0aCB0aGVpciB0aXRsZXMKYGBge3J9CmJhc2VEZXNpZ24gPC0gcmVhZF90c3YoJ292ZXJhbGxfZGVzaWduLnRzdicpCmRlc2lnblJOQSA8LSByZWFkX3RzdihmaWxlID0gJ1JOQS1zZXEvc2NhbmJfZ2VuZV9kZXNpZ24udHh0JykKZGVzaWduUk5BIDwtIG1hcF9kZnIoLnggPSBkZXNpZ25STkFbLDEwXSwuZiA9IH5zdHJfc3ViKHN0cmluZyA9IC54LDM3LDQzKSkgJT4lIAogIHVubmFtZSguKSAlPiUgCiAgdW5saXN0KC4pICU+JQogIG11dGF0ZShkZXNpZ25STkEsU3BlY2ltZW49LiwuYWZ0ZXIgPSAnIVNhbXBsZV90aXRsZScpClJOQUNvbG5hbWVzIDwtIHN0cl9yZW1vdmUoc3RyaW5nID0gY29sbmFtZXMoZGVzaWduUk5BKSxwYXR0ZXJuID0gJyEnKQpjb2xuYW1lcyhkZXNpZ25STkEpIDwtIFJOQUNvbG5hbWVzCmRlc2lnblJOQSA8LSBkZXNpZ25STkEgJT4lIAogIGZpbHRlcihzdHJfZGV0ZWN0KHN0cmluZyA9IFNhbXBsZV90aXRsZSxwYXR0ZXJuID0gJ3JlcGwnLG5lZ2F0ZSA9IFRSVUUpKSAlPiUgCiAgZHBseXI6OnNlbGVjdChTYW1wbGVfdGl0bGUsU3BlY2ltZW4pCgpkZXNpZ25STkFBbGwgPC0gbGVmdF9qb2luKGJhc2VEZXNpZ24sZGVzaWduUk5BLCJTcGVjaW1lbiIpICU+JSAKICBmaWx0ZXIoIWlzLm5hKEREQSkpICU+JSAKICBmaWx0ZXIoIWlzLm5hKERJQSkpICU+JSAKICBmaWx0ZXIoIWlzLm5hKFNhbXBsZV90aXRsZSkpCndyaXRlX3Rzdih4ID0gZGVzaWduUk5BQWxsLGZpbGUgPSAnUk5BLXNlcS9kZXNpZ25STkEudHN2JykKYGBgCgojIyBEaWZmZXJlbnRpYWwgRXhwcmVzc2lvbiBvZiBTaW5nbGUgQUlNUyBQQU01MCBzdWJ0eXBlcwogIAogIFRoaXMgZGVzaWduIGZpbGUgaXMgdXNlZCBmb3IgZGlmZmVyZW50aWFsIGV4cHJlc3Npb24gYW5hbHlzaXMgd2hlbiBjb21wYXJpbmcgMS12cy1yZXN0LiBTaW5jZSBvbmx5IERJQSBkYXRhIHdhcyB1c2VkIGZvciB0aGlzIHB1cnBvc2UsIHRoZSBjbGluaWNhbCBESUEgZGVzaWduIGZpbGUgaXMgdXNlZCBhcyBpbnB1dC4KYGBge3J9CmRlc2lnbkNsaW5pY2FsIDwtIHJlYWRfdHN2KCdESUEvRGVzaWduRmlsZXNESUEvZGVzaWduX2FpbTJfY2xpbmljYWwudHN2JykKZGVzaWduUk5BIDwtIHJlYWRfdHN2KCdSTkEtc2VxL2Rlc2lnblJOQS50c3YnKSAlPiUgCiAgZHBseXI6OnNlbGVjdChQb3NpdGlvbixTYW1wbGVfdGl0bGUpCgptZXJnZWREZXNpZ24gPC0gbGVmdF9qb2luKHggPSBkZXNpZ25STkEseSA9IGRlc2lnbkNsaW5pY2FsLCJQb3NpdGlvbiIpCk5ld1ZhbHVlcyA8LSBtZXJnZWREZXNpZ24kQUlNU19QQU01MCAlPiUgcGFzdGUwKCdBSU1TX1BBTTUwLicsLikKbWVyZ2VkRGVzaWduJEFJTVNfUEFNNTAgPC0gTmV3VmFsdWVzCm1lcmdlZERlc2lnbiRBSU1TX1BBTTUwIDwtIGFzX2ZhY3RvcihtZXJnZWREZXNpZ24kQUlNU19QQU01MCkKbWVyZ2VkRGVzaWduIDwtIG1lcmdlZERlc2lnbiAlPiUgbXV0YXRlKG9uZT0xKQptZXJnZWREZXNpZ24gPC0gbWVyZ2VkRGVzaWduICU+JSBwaXZvdF93aWRlcihuYW1lc19mcm9tID0gQUlNU19QQU01MCx2YWx1ZXNfZnJvbSA9IG9uZSx2YWx1ZXNfZmlsbCA9IDApICU+JSBtdXRhdGUoLixQQU01MD1tZXJnZWREZXNpZ24kQUlNU19QQU01MCkKCndyaXRlX3Rzdih4ID0gbWVyZ2VkRGVzaWduLGZpbGUgPSAnRElBL0Rlc2lnbkZpbGVzRElBL2Rlc2lnbl9haW0yX2NsaW5pY2FsX21hdGNoZWRfaW5kaXZpZHVhbHN1YnR5cGVzLnRzdicpCmBgYAoKIyMgRGlmZmVyZW50aWFsIEV4cHJlc3Npb24gb2Ygc3VidHlwZXMgYW5kIGNsaW5pY29wYXRob2xvZ2ljYWwgZmVhdHVyZXMgZm9yIERlY2lzaW9uIFRyZWUgbW9kZWwKICAKICBUaGlzIGRlc2lnbiBmaWxlIGlzIHVzZWQgZm9yIGRpZmZlcmVudGlhbCBleHByZXNzaW9uIGFuYWx5c2lzIG9mIGZlYXR1cmVzIHVzZWQgaW4gdGhlIGRlY2lzaW9uIHRyZWUgbW9kZWwuIE9ubHkgRElBIGRhdGEgaXMgdXNlZCBoZXJlLCBzbyB0aGUgY2xpbmljYWwgRElBIGZpbGUgaXMgdXNlZCBhcyBpbnB1dApgYGB7cn0KZGVzaWduIDwtIHJlYWRfdHN2KCdESUEvRGVzaWduRmlsZXNESUEvZGVzaWduX2FpbTJfY2xpbmljYWwudHN2JykgJT4lIAogIGZpbHRlcighaXMubmEoYExOIHN0YXR1c2ApKSAlPiUgCiAgZmlsdGVyKCFpcy5uYShHcmFkZSkpICU+JSAKICBmaWx0ZXIoIWlzLm5hKGBIRVIyIHN0YXR1c2ApKSAlPiUgCiAgZmlsdGVyKCFpcy5uYShBSU1TX1BBTTUwKSkKCmZpeENvbE5hbWVzIDwtIGNvbG5hbWVzKGRlc2lnbikgJT4lIAogIHN0cl9yZXBsYWNlX2FsbChzdHJpbmcgPSAuLHBhdHRlcm4gPSAnXFwgJyxyZXBsYWNlbWVudCA9ICdcXF8nKQpjb2xuYW1lcyhkZXNpZ24pIDwtIGZpeENvbE5hbWVzCgp3cml0ZV90c3YoeCA9IGRlc2lnbixmaWxlID0gJ0RJQS9EZXNpZ25GaWxlc0RJQS9kZXNpZ25fYWltMl9jbGluaWNhbF9EZWNpc2lvblRyZWUudHN2JykKYGBgCgoKIyBEYXRhIFByZS1wcm9jZXNzaW5nCgojIyBESUEKCiMjIyBESUEtTk4KCiMjIyMgRGF0YSBDbGVhbi11cApgYGB7cn0KZGF0YVAgPC0gJ0RJQS9EYXRhREUvRElBTk4vRElBc2NhbmIucHJfbWF0cml4LnRzdicKZGF0YUNsZWFuIDwtIHJlYWRfdHN2KGRhdGFQKSAlPiUgCiAgbmFfaWYoLiwwLjApICU+JSAKICBuYV9pZiguLDApCmRhdGFSZWR1Y2VkIDwtIGRhdGFDbGVhbiAlPiUgCiAgZHBseXI6OnNlbGVjdCgtYygxOjEwKSkKbmV3TmFtZXMgPC0gY29sbmFtZXMoZGF0YVJlZHVjZWQpICU+JSAKICBzdHJfZXh0cmFjdChzdHJpbmcgPSAuLHBhdHRlcm4gPSAnQlsxLTJdW0FCQ0RFRkdISV1bMS05XXxQb29sLionKSAlPiUgCiAgc3RyX3JlcGxhY2Uoc3RyaW5nID0gLixwYXR0ZXJuID0gJ1Bvb2xcXF9ESUFcXF9GdWxsV2luZG93XFxfMCcscmVwbGFjZW1lbnQgPSAnUCcpICU+JSAKICBzdHJfcmVtb3ZlKHN0cmluZyA9IC4scGF0dGVybiA9ICdcXC5tek1MXFwuZGlhJykKY29sbmFtZXMoZGF0YVJlZHVjZWQpIDwtIG5ld05hbWVzCmRhdGFDbGVhbiA8LSBkYXRhQ2xlYW4gJT4lIAogIGRwbHlyOjpzZWxlY3QoYygxOjEwKSkgJT4lIAogIGJpbmRfY29scyguLGRhdGFSZWR1Y2VkKQoKd3JpdGVfdHN2KHggPSBkYXRhQ2xlYW4sZmlsZSA9ICdESUEvRGF0YURFL0RJQU5OL0RJQXNjYW5iLnByX21hdHJpeF9jbGVhbi50c3YnKQpgYGAKCiMjIyMgUGVwdGlkZS1sZXZlbCBOb3JtYWxpemF0aW9uCmBgYHtyfQpqb2JOYW1lIDwtICdESUFfRElBTk5fcGVwdGlkZV9ub3JtYWxpc2F0aW9uJwpkYXRhUCA8LSAnRElBL0RhdGFERS9ESUFOTi9ESUFzY2FuYi5wcl9tYXRyaXhfY2xlYW4udHN2JwpkZXNpZ25QIDwtICdESUEvRGVzaWduRmlsZXNESUEvZGVzaWduX2FpbTJfY2xpbmljYWwudHN2JwpvdXRwdXRQIDwtICdESUEvRGF0YURFL0RJQU5OJwoKbm9ybWFseXplcihqb2JOYW1lID0gam9iTmFtZSxkZXNpZ25QYXRoID0gZGVzaWduUCxkYXRhUGF0aCA9IGRhdGFQLG91dHB1dERpciA9IG91dHB1dFAsbm9ybWFsaXplUmV0ZW50aW9uVGltZSA9IEZBTFNFLHplcm9Ub05BID0gVFJVRSxzYW1wbGVDb2xOYW1lID0gJ1Bvc2l0aW9uJyxncm91cENvbE5hbWUgPSAnZ3JvdXAnKQpgYGAKCiMjIyMgU29ydCBQZXB0aWRlcyBBbHBoYWJldGljYWxseQpgYGB7cn0Kc29ydElkcyA8LSBmdW5jdGlvbihkYXRhLHByb3RlaW5Db2x1bW4gPSAnUHJvdGVpbi5JZHMnLC4uLil7CiAgZGF0YSA8LSBwdWxsKGRhdGEscHJvdGVpbkNvbHVtbikgJT4lIAogICAgc3RyX3NwbGl0KHN0cmluZyA9IC4scGF0dGVybiA9ICc7JykgJT4lIAogICAgbWFwKC54ID0gLiwuZiA9IHN0cl9zb3J0KSAlPiUgCiAgICBtYXAoLnggPSAuLC5mID0gZnVuY3Rpb24oeCkgcGFzdGUwKHgsY29sbGFwc2UgPSAnOycpKSAlPiUgCiAgICB1bmxpc3QgJT4lIAogICAgbXV0YXRlKGRhdGEsUHJvdGVpbi5JRD0uLC5iZWZvcmU9Y29sbmFtZXMoZGF0YVsyXSkpICU+JSAKICAgIGRwbHlyOjpzZWxlY3QoLXByb3RlaW5Db2x1bW4pCiAgbmFtZXMoZGF0YSlbbmFtZXMoZGF0YSk9PSdQcm90ZWluLklEJ10gPC0gJ1Byb3RlaW4nCiAgcmV0dXJuKGRhdGEpCn0KYGBgCmBgYHtyfQpkYXRhIDwtIHJlYWRfdHN2KGZpbGUgPSAnRElBL0RhdGFERS9ESUFOTi9ESUFfRElBTk5fcGVwdGlkZV9ub3JtYWxpc2F0aW9uL0N5Y0xvZXNzLW5vcm1hbGl6ZWQudHh0JykgJT4lCiAgc29ydElkcyhkYXRhID0gLixwcm90ZWluQ29sdW1uID0gJ1Byb3RlaW4uSWRzJykKCndyaXRlX3Rzdih4ID0gZGF0YSxmaWxlID0gJ0RJQS9EYXRhREUvRElBTk4vRElBX0RJQU5OX3BlcHRpZGVfbm9ybWFsaXNhdGlvbi9DeWNMb2Vzc19ub3JtX3NvcnRlZC50c3YnKQpgYGAKCiMjIyMgUHJvdGVpbiBSb2xsdXAKYGBge2Jhc2h9ClJzY3JpcHQgfi9TQ0FOQl9QVUJMSUNBVElPTi9BbGdvcml0aG1zL1Byb3RlaW5Sb2xsdXAtbWFzdGVyL1IvcHJvdGVpbl9yb2xsdXAuUiBcCiAgICAtLXJkZl9mcCB+L1NDQU5CX1BVQkxJQ0FUSU9OL0RJQS9EYXRhREUvRElBTk4vRElBX0RJQU5OX3BlcHRpZGVfbm9ybWFsaXNhdGlvbi9DeWNMb2Vzc19ub3JtX3NvcnRlZC50c3YgXAogICAgLS1kZGZfZnAgfi9TQ0FOQl9QVUJMSUNBVElPTi9ESUEvRGVzaWduRmlsZXNESUEvZGVzaWduX2FpbTJfY2xpbmljYWwudHN2IFwKICAgIC0tc2FtcGxlX2NvbCBQb3NpdGlvbiBcCiAgICAtLXByb3RlaW5fY29sIFByb3RlaW4gXAogICAgLS1vdXRfZnAgfi9TQ0FOQl9QVUJMSUNBVElPTi9ESUEvRGF0YURFL0RJQU5OL0RJQV9ESUFOTl9Qcm90ZWluX1JSb2xsdXAvRElBX0RJQU5OX1JSb2xsdXAudHN2CmBgYAoKIyMjIyBBZGRpbmcgR2VuZSBJbmZvcm1hdGlvbgpgYGB7cn0KZGlhUCA8LSAnRElBL0RhdGFERS9ESUFOTi9ESUFfRElBTk5fUHJvdGVpbl9SUm9sbHVwL0RJQV9ESUFOTl9SUm9sbHVwLnRzdicKZGlhbm5QIDwtICdESUEvRGF0YURFL0RJQU5OL0RJQV9ESUFOTl9wZXB0aWRlX25vcm1hbGlzYXRpb24vQ3ljTG9lc3Nfbm9ybV9zb3J0ZWQudHN2JwpkaWFEYXRhIDwtIHJlYWRfdHN2KGRpYVApCmRpYW5uIDwtIHJlYWRfdHN2KGRpYW5uUCkKZGlhbm5SZWR1Y2VkIDwtIGRpYW5uICU+JSAKICBkcGx5cjo6c2VsZWN0KFByb3RlaW4sUHJvdGVpbi5OYW1lcyxGaXJzdC5Qcm90ZWluLkRlc2NyaXB0aW9uLEdlbmVzKQpkaWFEYXRhR2VuZXMgPC0gbGVmdF9qb2luKHggPSBkaWFEYXRhLHkgPSBkaWFublJlZHVjZWQsYnkgPSAiUHJvdGVpbiIpICU+JSAKICBmaWx0ZXIoLiwhZHVwbGljYXRlZChQcm90ZWluKSkKZGlhRGF0YUdlbmVzIDwtIHJlbG9jYXRlKGRpYURhdGFHZW5lcyx3aGVyZSh+aXMuY2hhcmFjdGVyKC54KSksLmFmdGVyID0gJ1Byb3RlaW4nKQp3cml0ZV90c3YoeCA9IGRpYURhdGFHZW5lcyxmaWxlID0gJ0RJQS9EYXRhREUvRElBTk4vRElBX0RJQU5OX1Byb3RlaW5fUlJvbGx1cC9ESUFfRElBTk5fUlJvbGx1cF9HZW5lcy50c3YnKQpgYGAKCgojIyMgRW5jeWNsb3BlRElBCgojIyMjIERhdGEgQ2xlYW4tdXAKYGBge3J9CmRhdGFQIDwtICdESUEvRGF0YURFL0VuY3ljbG9wZURJQS9TQ0FOQl90d29wbGF0ZXMucGVwdGlkZXMudHh0JwpyZWFkX3RzdihkYXRhUCkgJT4lIAogIG5hX2lmKC4sMC4wKSAlPiUgCiAgbmFfaWYoLiwwKSAlPiUgCiAgcmVsb2NhdGUoLiwnUHJvdGVpbicsLmJlZm9yZT0nUGVwdGlkZScpICU+JSAKICB3cml0ZV90c3YoeCA9IC4sZmlsZSA9ICdESUEvRGF0YURFL0VuY3ljbG9wZURJQS9TQ0FOQl90d29wbGF0ZXMucGVwdGlkZXNfY2xlYW4udHh0JykKYGBgCgojIyMjIFBlcHRpZGUtbGV2ZWwgTm9ybWFsaXphdGlvbgpgYGB7cn0Kam9iTmFtZSA8LSAnRElBX0VuY3ljbG9wZURJQV9wZXB0aWRlX25vcm1hbGlzYXRpb24nCmRhdGFQIDwtICdESUEvRGF0YURFL0VuY3ljbG9wZURJQS9TQ0FOQl90d29wbGF0ZXMucGVwdGlkZXNfY2xlYW4udHh0JwpkZXNpZ25QIDwtICdESUEvRGVzaWduRmlsZXNESUEvZGVzaWduX2FpbTJfY2xpbmljYWwudHN2JwpvdXRwdXRQIDwtICdESUEvRGF0YURFL0VuY3ljbG9wZURJQScKCm5vcm1hbHl6ZXIoam9iTmFtZSA9IGpvYk5hbWUsZGVzaWduUGF0aCA9IGRlc2lnblAsZGF0YVBhdGggPSBkYXRhUCxvdXRwdXREaXIgPSBvdXRwdXRQLG5vcm1hbGl6ZVJldGVudGlvblRpbWUgPSBGQUxTRSx6ZXJvVG9OQSA9IEZBTFNFLHNhbXBsZUNvbE5hbWUgPSAnRElBJyxncm91cENvbE5hbWUgPSAnZ3JvdXAnKQpgYGAKCiMjIyMgU29ydCBQZXB0aWRlcyBBbHBoYWJldGljYWxseQpgYGB7cn0Kc29ydElkcyA8LSBmdW5jdGlvbihkYXRhLHByb3RlaW5Db2x1bW4gPSAnUHJvdGVpbicsLi4uKXsKICBkYXRhIDwtIHB1bGwoZGF0YSxwcm90ZWluQ29sdW1uKSAlPiUgCiAgICBzdHJfc3BsaXQoc3RyaW5nID0gLixwYXR0ZXJuID0gJzsnKSAlPiUgCiAgICBtYXAoLnggPSAuLC5mID0gc3RyX3NvcnQpICU+JSAKICAgIG1hcCgueCA9IC4sLmYgPSBmdW5jdGlvbih4KSBwYXN0ZTAoeCxjb2xsYXBzZSA9ICc7JykpICU+JSAKICAgIHVubGlzdCAlPiUgCiAgICBtdXRhdGUoZGF0YSxQcm90ZWluLklEPS4sLmJlZm9yZT1jb2xuYW1lcyhkYXRhWzJdKSkgJT4lIAogICAgZHBseXI6OnNlbGVjdCgtcHJvdGVpbkNvbHVtbikKICBuYW1lcyhkYXRhKVtuYW1lcyhkYXRhKT09J1Byb3RlaW4uSUQnXSA8LSAnUHJvdGVpbicKICByZXR1cm4oZGF0YSkKfQpgYGAKYGBge3J9CmRhdGEgPC0gcmVhZF90c3YoZmlsZSA9ICdESUEvRGF0YURFL0VuY3ljbG9wZURJQS9ESUFfRW5jeWNsb3BlRElBX3BlcHRpZGVfbm9ybWFsaXNhdGlvbi9DeWNMb2Vzcy1ub3JtYWxpemVkLnR4dCcpICU+JSAKICBzb3J0SWRzKGRhdGEgPSAuLHByb3RlaW5Db2x1bW4gPSAnUHJvdGVpbicpCgp3cml0ZV90c3YoeCA9IGRhdGEsZmlsZSA9ICdESUEvRGF0YURFL0VuY3ljbG9wZURJQS9ESUFfRW5jeWNsb3BlRElBX3BlcHRpZGVfbm9ybWFsaXNhdGlvbi9DeWNMb2Vzc19ub3JtX3NvcnRlZC50c3YnKQpgYGAKCiMjIyMgUHJvdGVpbiBSb2xsdXAKYGBge2Jhc2h9ClJzY3JpcHQgfi9TQ0FOQl9QVUJMSUNBVElPTi9BbGdvcml0aG1zL1Byb3RlaW5Sb2xsdXAtbWFzdGVyL1IvcHJvdGVpbl9yb2xsdXAuUiBcCiAgICAtLXJkZl9mcCB+L1NDQU5CX1BVQkxJQ0FUSU9OL0RJQS9EYXRhREUvRW5jeWNsb3BlRElBL0RJQV9FbmN5Y2xvcGVESUFfcGVwdGlkZV9ub3JtYWxpc2F0aW9uL0N5Y0xvZXNzX25vcm1fc29ydGVkLnRzdiBcCiAgICAtLWRkZl9mcCB+L1NDQU5CX1BVQkxJQ0FUSU9OL0RJQS9EZXNpZ25GaWxlc0RJQS9kZXNpZ25fYWltMl9jbGluaWNhbC50c3YgXAogICAgLS1zYW1wbGVfY29sIERJQSBcCiAgICAtLXByb3RlaW5fY29sIFByb3RlaW4gXAogICAgLS1vdXRfZnAgfi9TQ0FOQl9QVUJMSUNBVElPTi9ESUEvRGF0YURFL0VuY3ljbG9wZURJQS9ESUFfRW5jeWNsb3BlRElBX1Byb3RlaW5fUlJvbHVwL0RJQV9FbmN5Y2xvcGVESUFfUlJvbGx1cC50c3YKYGBgCgojIyMjIEFkZGluZyBHZW5lIEluZm9ybWF0aW9uCmBgYHtyfQojIFNldHRpbmcgQmlvbWFSdApIU2FwaWVuc01hcnQgPC0gdXNlTWFydChiaW9tYXJ0ID0gJ0VOU0VNQkxfTUFSVF9FTlNFTUJMJykKSFNhcGllbnNNYXJ0IDwtIHVzZURhdGFzZXQoZGF0YXNldCA9ICdoc2FwaWVuc19nZW5lX2Vuc2VtYmwnLG1hcnQgPSBIU2FwaWVuc01hcnQpCmBgYApgYGB7cn0KZGF0YURJQSA8LSByZWFkX3RzdihmaWxlID0gJ0RJQS9EYXRhREUvRW5jeWNsb3BlRElBL0RJQV9FbmN5Y2xvcGVESUFfUHJvdGVpbl9SUm9sdXAvRElBX0VuY3ljbG9wZURJQV9SUm9sbHVwLnRzdicpCmNsZWFuSURzIDwtIGRhdGFESUEkUHJvdGVpbiAlPiUgCiAgc3RyX3NwbGl0KHN0cmluZyA9IC4scGF0dGVybiA9ICc7JykgJT4lIAogIHN0cl9leHRyYWN0X2FsbChzdHJpbmcgPSAuLHBhdHRlcm4gPSAnKD88PVs6c3ltYm9sOl0pWzphbG51bTpdKyg/PVs6c3ltYm9sOl0pJykgJT4lIAogIG1hcCgueCA9IC4sLmYgPSB+cGFzdGUwKC54LGNvbGxhcHNlID0gJzsnKSkgJT4lCiAgdW5saXN0CmRhdGFESUEkUHJvdGVpbiA8LSBjbGVhbklEcwoKIyBBZGQgR2VuZSBJbmZvcm1hdGlvbgpwcm90ZWluSWRzRElBIDwtIGRhdGFESUEkUHJvdGVpbiAlPiUgCiAgc3RyX3NwbGl0KHN0cmluZyA9IC4scGF0dGVybiA9ICc7JykKbmFtZXMocHJvdGVpbklkc0RJQSkgPC0gZGF0YURJQSRQcm90ZWluCmdlbmVOYW1lc0RJQSA8LSBtYXAoLnggPSBwcm90ZWluSWRzRElBLC5mID0gfmdldEJNKG1hcnQ9SFNhcGllbnNNYXJ0LHZhbHVlcyA9IC54LGF0dHJpYnV0ZXMgPSAndW5pcHJvdF9nbl9zeW1ib2wnLGZpbHRlcnMgPSAndW5pcHJvdHN3aXNzcHJvdCcpKSAlPiUKICBtYXAoLix1bmxpc3QpCgpESUFHZW5lTmFtZXMgPC0gZ2VuZU5hbWVzRElBICU+JSAKICBtYXAoLnggPSAuLC5mID0gfnBhc3RlMCgueCxjb2xsYXBzZSA9ICc7JykpICU+JQogIHVubmFtZSAlPiUgCiAgdW5saXN0CmRhdGFESUEgPC0gZGF0YURJQSAlPiUgCiAgbXV0YXRlKEdlbmU9RElBR2VuZU5hbWVzLC5hZnRlciA9IFByb3RlaW4pCndyaXRlX3Rzdih4ID0gZGF0YURJQSxmaWxlID0gJ0RJQS9EYXRhREUvRW5jeWNsb3BlRElBL0RJQV9FbmN5Y2xvcGVESUFfUHJvdGVpbl9SUm9sdXAvRElBX0VuY3ljbG9wZURJQV9SUm9sbHVwX0dlbmVzLnRzdicpCmBgYApgYGB7cn0KIyBTaG9ydCBuYW1lcyBmb3IgdGhlIHNhbXBsZXMKZGF0YSA8LSByZWFkX3RzdihmaWxlID0gJ0RJQS9EYXRhREUvRW5jeWNsb3BlRElBL0RJQV9FbmN5Y2xvcGVESUFfUHJvdGVpbl9SUm9sdXAvRElBX0VuY3ljbG9wZURJQV9SUm9sbHVwX0dlbmVzLnRzdicpCmRlc2lnbiA8LSByZWFkX3RzdihmaWxlID0gJ0RJQS9EZXNpZ25GaWxlc0RJQS9kZXNpZ25fYWltMl9jbGluaWNhbC50c3YnKQoKY29sbmFtZXMoZGF0YSkgPC0gY29sbmFtZXMoZGF0YSkgJT4lIAogIHN0cl9yZW1vdmUoc3RyaW5nID0gLixwYXR0ZXJuID0gJ15YKD89WzphbG51bTpdKScpCgpkYXRhUmVkdWNlZCA8LSBkYXRhICU+JSAKICBkcGx5cjo6c2VsZWN0KGRlc2lnbiRESUEpCmNvbG5hbWVzKGRhdGFSZWR1Y2VkKSA8LSBkZXNpZ24kUG9zaXRpb24KCm5ld0RhdGEgPC0gZGF0YSAlPiUgCiAgZHBseXI6OnNlbGVjdChjKDE6NCkpICU+JSAKICBiaW5kX2NvbHMoLixkYXRhUmVkdWNlZCkKCndyaXRlX3Rzdih4ID0gbmV3RGF0YSxmaWxlID0gJ0RJQS9EYXRhREUvRW5jeWNsb3BlRElBL0RJQV9FbmN5Y2xvcGVESUFfUHJvdGVpbl9SUm9sdXAvRElBX0VuY3ljbG9wZURJQV9SUm9sbHVwX0dlbmVzLnRzdicpCmBgYAoKCiMjIEREQQoKIyMjIEREQSBEYXRhIGZvciBDb3JyZWxhdGlvbgoKIyMjIyBNYXRjaCBCZXR3ZWVuIFJ1bnMKCiMjIyMjIFByZXBhcmUgRmlsZXMgd2l0aCBzaG9ydG5hbWVzCmBgYHtyfQpkYXRhUE1CUiA8LSAnRERBL0RhdGFDb3JyZWxhdGlvbi9NQlIvc2NhbmJfYWltMl91bmlwcm90X21ici9wZXB0aWRlcy50eHQnCmRhdGFNQlIgPC0gcmVhZF90c3YoZGF0YVBNQlIsY29sX25hbWVzID0gVFJVRSkgJT4lIAogIGRwbHlyOjpzZWxlY3QoUHJvdGVpbnMsYExlYWRpbmcgcmF6b3IgcHJvdGVpbmAsU2VxdWVuY2UsY29udGFpbnMoJ0xGUScpKQpkYXRhTUJSUmVkdWNlZCA8LSBkYXRhTUJSICU+JQogIGRwbHlyOjpzZWxlY3QoY29udGFpbnMoJ0xGUScpKQpkYXRhTUJSTmFtZXMgPC0gZGF0YU1CUlJlZHVjZWQgJT4lIAogIGNvbG5hbWVzICU+JSAKICBzdHJfZXh0cmFjdChzdHJpbmcgPSAuLHBhdHRlcm4gPSAnQls6ZGlnaXQ6XS4rfFBvb2wuKicpICU+JSAKICBzdHJfcmVwbGFjZShzdHJpbmcgPSAuLHBhdHRlcm4gPSAnUG9vbC4qJyxyZXBsYWNlbWVudCA9ICdQb29sX0REQV8wMScpCmNvbG5hbWVzKGRhdGFNQlJSZWR1Y2VkKSA8LSBkYXRhTUJSTmFtZXMKZGF0YU1CUiA8LSBkYXRhTUJSICU+JSAKICB0cmFuc211dGUoUHJvdGVpbnM9UHJvdGVpbnMsTGVhZGluZy5SYXpvci5Qcm90ZWluPWBMZWFkaW5nIHJhem9yIHByb3RlaW5gLFNlcXVlbmNlPVNlcXVlbmNlLGRhdGFNQlJSZWR1Y2VkKQpkYXRhTUJSIDwtIGRhdGFNQlIgJT4lIAogIGZpbHRlcihzdHJfZGV0ZWN0KHN0cmluZyA9IFByb3RlaW5zLHBhdHRlcm4gPSAnQ09OXFxfJyxuZWdhdGUgPSBUUlVFKSkKCndyaXRlX3Rzdih4ID0gZGF0YU1CUixmaWxlID0gJ0REQS9EYXRhQ29ycmVsYXRpb24vTUJSL3BlcHRpZGVzX21icl9zaG9ydG5hbWVzLnRzdicpCmBgYAoKIyMjIyMgUGVwdGlkZS1sZXZlbCBOb3JtYWxpemF0aW9uCmBgYHtyfQpqb2JNQlIgPC0gJ0REQV9NQlJfcGVwdGlkZV9ub3JtYWxpc2F0aW9uJwpkYXRhUE1CUiA8LSAnRERBL0RhdGFDb3JyZWxhdGlvbi9NQlIvcGVwdGlkZXNfbWJyX3Nob3J0bmFtZXMudHN2JwpkZXNpZ25QIDwtICdEREEvRGVzaWduRmlsZXNEREEvZGVzaWduX2FpbTJfY2xpbmljYWwudHN2JwpvdXRwdXRQTUJSIDwtICdEREEvRGF0YUNvcnJlbGF0aW9uL01CUicKCm5vcm1hbHl6ZXIoam9iTmFtZSA9IGpvYk1CUixkZXNpZ25QYXRoID0gZGVzaWduUCxkYXRhUGF0aCA9IGRhdGFQTUJSLG91dHB1dERpciA9IG91dHB1dFBNQlIscmVxdWlyZVJlcGxpY2F0ZXMgPSBGQUxTRSxub3JtYWxpemVSZXRlbnRpb25UaW1lID0gRkFMU0UsemVyb1RvTkEgPSBUUlVFLHNhbXBsZUNvbE5hbWUgPSAnUG9zaXRpb24nLGdyb3VwQ29sTmFtZSA9ICdncm91cCcpCmBgYAoKIyMjIyMgU29ydCBQZXB0aWRlcyBBbHBoYWJldGljYWxseQpgYGB7cn0Kc29ydElkcyA8LSBmdW5jdGlvbihkYXRhLHByb3RlaW5Db2x1bW4gPSAnUHJvdGVpbnMnLC4uLil7CiAgZGF0YSA8LSBwdWxsKGRhdGEscHJvdGVpbkNvbHVtbikgJT4lIAogICAgc3RyX3NwbGl0KHN0cmluZyA9IC4scGF0dGVybiA9ICc7JykgJT4lIAogICAgbWFwKC54ID0gLiwuZiA9IHN0cl9zb3J0KSAlPiUgCiAgICBtYXAoLnggPSAuLC5mID0gZnVuY3Rpb24oeCkgcGFzdGUwKHgsY29sbGFwc2UgPSAnOycpKSAlPiUgCiAgICB1bmxpc3QgJT4lIAogICAgbXV0YXRlKGRhdGEsUHJvdGVpbi5JRD0uLC5iZWZvcmU9Y29sbmFtZXMoZGF0YVsyXSkpICU+JSAKICAgIGRwbHlyOjpzZWxlY3QoLXByb3RlaW5Db2x1bW4pCiAgbmFtZXMoZGF0YSlbbmFtZXMoZGF0YSk9PSdQcm90ZWluLklEJ10gPC0gJ1Byb3RlaW4nCiAgcmV0dXJuKGRhdGEpCn0KYGBgCmBgYHtyfQpkYXRhTUJSIDwtIHJlYWRfdHN2KGZpbGUgPSAnRERBL0RhdGFDb3JyZWxhdGlvbi9NQlIvRERBX01CUl9wZXB0aWRlX25vcm1hbGlzYXRpb24vQ3ljTG9lc3Mtbm9ybWFsaXplZC50eHQnKSAlPiUgCiAgc29ydElkcyhkYXRhID0gLixwcm90ZWluQ29sdW1uID0gJ1Byb3RlaW5zJykKCndyaXRlX3Rzdih4ID0gZGF0YU1CUixmaWxlID0gJ0REQS9EYXRhQ29ycmVsYXRpb24vTUJSL0REQV9NQlJfcGVwdGlkZV9ub3JtYWxpc2F0aW9uL0N5Y0xvZXNzX25vcm1fc29ydGVkLnRzdicpCmBgYAoKIyMjIyMgUHJvdGVpbiBSb2xsdXAKYGBge2Jhc2h9ClJzY3JpcHQgfi9TQ0FOQl9QVUJMSUNBVElPTi9BbGdvcml0aG1zL1Byb3RlaW5Sb2xsdXAtbWFzdGVyL1IvcHJvdGVpbl9yb2xsdXAuUiBcCiAgICAtLXJkZl9mcCB+L1NDQU5CX1BVQkxJQ0FUSU9OL0REQS9EYXRhQ29ycmVsYXRpb24vTUJSL0REQV9NQlJfcGVwdGlkZV9ub3JtYWxpc2F0aW9uL0N5Y0xvZXNzX25vcm1fc29ydGVkLnRzdiBcCiAgICAtLWRkZl9mcCB+L1NDQU5CX1BVQkxJQ0FUSU9OL0REQS9EZXNpZ25GaWxlc0REQS9kZXNpZ25fYWltMl9jbGluaWNhbC50c3YgXAogICAgLS1zYW1wbGVfY29sIFBvc2l0aW9uIFwKICAgIC0tcHJvdGVpbl9jb2wgUHJvdGVpbiBcCiAgICAtLW91dF9mcCB+L1NDQU5CX1BVQkxJQ0FUSU9OL0REQS9EYXRhQ29ycmVsYXRpb24vTUJSL0REQV9NQlJfUHJvdGVpbl9Sb2xsdXAvRERBX01CUl9SUm9sbHVwLnRzdgpgYGAKCiMjIyMjIEFkZCBHZW5lIEluZm9ybWF0aW9uCmBgYHtyfQpIU2FwaWVuc01hcnQgPC0gdXNlTWFydChiaW9tYXJ0ID0gJ0VOU0VNQkxfTUFSVF9FTlNFTUJMJykKSFNhcGllbnNNYXJ0IDwtIHVzZURhdGFzZXQoZGF0YXNldCA9ICdoc2FwaWVuc19nZW5lX2Vuc2VtYmwnLG1hcnQgPSBIU2FwaWVuc01hcnQpCmBgYApgYGB7cn0KZGF0YU1CUiA8LSByZWFkX3RzdihmaWxlID0gJ0REQS9EYXRhQ29ycmVsYXRpb24vTUJSL0REQV9NQlJfUHJvdGVpbl9Sb2xsdXAvRERBX01CUl9SUm9sbHVwLnRzdicpCnByb3RlaW5JZHNNQlIgPC0gZGF0YU1CUiRQcm90ZWluICU+JSAKICBzdHJfc3BsaXQoc3RyaW5nID0gLixwYXR0ZXJuID0gJzsnKQpuYW1lcyhwcm90ZWluSWRzTUJSKSA8LSBkYXRhTUJSJFByb3RlaW4KZ2VuZU5hbWVzTUJSIDwtIG1hcCgueCA9IHByb3RlaW5JZHNNQlIsLmYgPSB+Z2V0Qk0obWFydD1IU2FwaWVuc01hcnQsdmFsdWVzID0gLngsYXR0cmlidXRlcyA9ICd1bmlwcm90X2duX3N5bWJvbCcsZmlsdGVycyA9ICd1bmlwcm90c3dpc3Nwcm90JykpICU+JQogIG1hcCguLHVubGlzdCkKCk1CUkdlbmVOYW1lcyA8LSBnZW5lTmFtZXNNQlIgJT4lIAogIG1hcCgueCA9IC4sLmYgPSB+cGFzdGUwKC54LGNvbGxhcHNlID0gJzsnKSkgJT4lCiAgdW5uYW1lICU+JSAKICB1bmxpc3QKZGF0YU1CUiA8LSBkYXRhTUJSICU+JSAKICBtdXRhdGUoR2VuZT1NQlJHZW5lTmFtZXMsLmFmdGVyID0gUHJvdGVpbikKd3JpdGVfdHN2KHggPSBkYXRhTUJSLGZpbGUgPSAnRERBL0RhdGFDb3JyZWxhdGlvbi9NQlIvRERBX01CUl9Qcm90ZWluX1JvbGx1cC9EREFfTUJSX1JSb2xsdXBfR2VuZXMudHN2JykKYGBgCgojIyMjIE5vIE1hdGNoIEJldHdlZW4gUnVucwoKIyMjIyMgUHJlcGFyZSBGaWxlcyB3aXRoIHNob3J0IG5hbWVzCmBgYHtyfQpkYXRhUG5vTUJSIDwtICdEREEvRGF0YUNvcnJlbGF0aW9uL25vTUJSL3NjYW5iX2FpbTJfdW5pcHJvdF9ub19tYnIvcGVwdGlkZXMudHh0JwpkYXRhTm9NQlIgPC0gcmVhZF90c3YoZGF0YVBub01CUixjb2xfbmFtZXMgPSBUUlVFKSAlPiUgCiAgZHBseXI6OnNlbGVjdChQcm90ZWlucyxgTGVhZGluZyByYXpvciBwcm90ZWluYCxTZXF1ZW5jZSxjb250YWlucygnTEZRJykpCmRhdGFOb01CUlJlZHVjZWQgPC0gZGF0YU5vTUJSICU+JQogIGRwbHlyOjpzZWxlY3QoY29udGFpbnMoJ0xGUScpKQpkYXRhTm9NQlJOYW1lcyA8LSBkYXRhTm9NQlJSZWR1Y2VkICU+JSAKICBjb2xuYW1lcyAlPiUgCiAgc3RyX2V4dHJhY3Qoc3RyaW5nID0gLixwYXR0ZXJuID0gJ0JbOmRpZ2l0Ol0uK3xQb29sLionKSAlPiUgCiAgc3RyX3JlcGxhY2Uoc3RyaW5nID0gLixwYXR0ZXJuID0gJ1Bvb2wuKicscmVwbGFjZW1lbnQgPSAnUG9vbF9EREFfMDEnKQpjb2xuYW1lcyhkYXRhTm9NQlJSZWR1Y2VkKSA8LSBkYXRhTm9NQlJOYW1lcwpkYXRhTm9NQlIgPC0gZGF0YU5vTUJSICU+JSAKICB0cmFuc211dGUoUHJvdGVpbnM9UHJvdGVpbnMsTGVhZGluZy5SYXpvci5Qcm90ZWluPWBMZWFkaW5nIHJhem9yIHByb3RlaW5gLFNlcXVlbmNlPVNlcXVlbmNlLGRhdGFOb01CUlJlZHVjZWQpCmRhdGFOb01CUiA8LSBkYXRhTm9NQlIgJT4lIAogIGZpbHRlcihzdHJfZGV0ZWN0KHN0cmluZyA9IFByb3RlaW5zLHBhdHRlcm4gPSAnQ09OXFxfJyxuZWdhdGUgPSBUUlVFKSkKCndyaXRlX3Rzdih4ID0gZGF0YU5vTUJSLGZpbGUgPSAnRERBL0RhdGFDb3JyZWxhdGlvbi9ub01CUi9wZXB0aWRlc19ub21icl9zaG9ydG5hbWVzLnRzdicpCmBgYAoKIyMjIyMgUGVwdGlkZS1sZXZlbCBOb3JtYWxpemF0aW9uCmBgYHtyfQpqb2JOb01CUiA8LSAnRERBX25vX01CUl9wZXB0aWRlX25vcm1hbGlzYXRpb24nCmRhdGFQTm9NQlIgPC0gJ0REQS9EYXRhQ29ycmVsYXRpb24vbm9NQlIvcGVwdGlkZXNfbm9tYnJfc2hvcnRuYW1lcy50c3YnCmRlc2lnblAgPC0gJ0REQS9EZXNpZ25GaWxlc0REQS9kZXNpZ25fYWltMl9jbGluaWNhbC50c3YnCm91dHB1dFBOb01CUiA8LSAnRERBL0RhdGFDb3JyZWxhdGlvbi9ub01CUicKCm5vcm1hbHl6ZXIoam9iTmFtZSA9IGpvYk5vTUJSLGRlc2lnblBhdGggPSBkZXNpZ25QLGRhdGFQYXRoID0gZGF0YVBOb01CUixvdXRwdXREaXIgPSBvdXRwdXRQTm9NQlIscmVxdWlyZVJlcGxpY2F0ZXMgPSBGQUxTRSxub3JtYWxpemVSZXRlbnRpb25UaW1lID0gRkFMU0UsemVyb1RvTkEgPSBUUlVFLHNhbXBsZUNvbE5hbWUgPSAnUG9zaXRpb24nLGdyb3VwQ29sTmFtZSA9ICdncm91cCcpCmBgYAoKIyMjIyMgU29ydCBQZXB0aWRlcyBBbHBoYWJldGljYWxseQpgYGB7cn0Kc29ydElkcyA8LSBmdW5jdGlvbihkYXRhLHByb3RlaW5Db2x1bW4gPSAnUHJvdGVpbnMnLC4uLil7CiAgZGF0YSA8LSBwdWxsKGRhdGEscHJvdGVpbkNvbHVtbikgJT4lIAogICAgc3RyX3NwbGl0KHN0cmluZyA9IC4scGF0dGVybiA9ICc7JykgJT4lIAogICAgbWFwKC54ID0gLiwuZiA9IHN0cl9zb3J0KSAlPiUgCiAgICBtYXAoLnggPSAuLC5mID0gZnVuY3Rpb24oeCkgcGFzdGUwKHgsY29sbGFwc2UgPSAnOycpKSAlPiUgCiAgICB1bmxpc3QgJT4lIAogICAgbXV0YXRlKGRhdGEsUHJvdGVpbi5JRD0uLC5iZWZvcmU9Y29sbmFtZXMoZGF0YVsyXSkpICU+JSAKICAgIGRwbHlyOjpzZWxlY3QoLXByb3RlaW5Db2x1bW4pCiAgbmFtZXMoZGF0YSlbbmFtZXMoZGF0YSk9PSdQcm90ZWluLklEJ10gPC0gJ1Byb3RlaW4nCiAgcmV0dXJuKGRhdGEpCn0KYGBgCmBgYHtyfQpkYXRhTm9NQlIgPC0gcmVhZF90c3YoZmlsZSA9ICdEREEvRGF0YUNvcnJlbGF0aW9uL25vTUJSL0REQV9ub19NQlJfcGVwdGlkZV9ub3JtYWxpc2F0aW9uL0N5Y0xvZXNzLW5vcm1hbGl6ZWQudHh0JykgJT4lIAogIHNvcnRJZHMoZGF0YSA9IC4scHJvdGVpbkNvbHVtbiA9ICdQcm90ZWlucycpCgp3cml0ZV90c3YoeCA9IGRhdGFOb01CUixmaWxlID0gJ0REQS9EYXRhQ29ycmVsYXRpb24vbm9NQlIvRERBX25vX01CUl9wZXB0aWRlX25vcm1hbGlzYXRpb24vQ3ljTG9lc3Nfbm9ybV9zb3J0ZWQudHN2JykKYGBgCgojIyMjIyBQcm90ZWluIFJvbGx1cApgYGB7YmFzaH0KUnNjcmlwdCB+L1NDQU5CX1BVQkxJQ0FUSU9OL0FsZ29yaXRobXMvUHJvdGVpblJvbGx1cC1tYXN0ZXIvUi9wcm90ZWluX3JvbGx1cC5SIFwKICAgIC0tcmRmX2ZwIH4vU0NBTkJfUFVCTElDQVRJT04vRERBL0RhdGFDb3JyZWxhdGlvbi9ub01CUi9EREFfbm9fTUJSX3BlcHRpZGVfbm9ybWFsaXNhdGlvbi9DeWNMb2Vzc19ub3JtX3NvcnRlZC50c3YgXAogICAgLS1kZGZfZnAgfi9TQ0FOQl9QVUJMSUNBVElPTi9EREEvRGVzaWduRmlsZXNEREEvZGVzaWduX2FpbTJfY2xpbmljYWwudHN2IFwKICAgIC0tc2FtcGxlX2NvbCBQb3NpdGlvbiBcCiAgICAtLXByb3RlaW5fY29sIFByb3RlaW4gXAogICAgLS1vdXRfZnAgfi9TQ0FOQl9QVUJMSUNBVElPTi9EREEvRGF0YUNvcnJlbGF0aW9uL25vTUJSL0REQV9ub01CUl9Qcm90ZWluX1JSb2xsdXAvRERBX25vTUJSX1JSb2x1cC50c3YKYGBgCgojIyMjIyBBZGQgR2VuZSBJbmZvcm1hdGlvbgpgYGB7cn0KSFNhcGllbnNNYXJ0IDwtIHVzZU1hcnQoYmlvbWFydCA9ICdFTlNFTUJMX01BUlRfRU5TRU1CTCcpCkhTYXBpZW5zTWFydCA8LSB1c2VEYXRhc2V0KGRhdGFzZXQgPSAnaHNhcGllbnNfZ2VuZV9lbnNlbWJsJyxtYXJ0ID0gSFNhcGllbnNNYXJ0KQpgYGAKYGBge3J9CmRhdGFOb01CUiA8LSByZWFkX3RzdihmaWxlID0gJ0REQS9EYXRhQ29ycmVsYXRpb24vbm9NQlIvRERBX25vTUJSX1Byb3RlaW5fUlJvbGx1cC9EREFfbm9NQlJfUlJvbHVwLnRzdicpCnByb3RlaW5JZHNOb01CUiA8LSBkYXRhTm9NQlIkUHJvdGVpbiAlPiUgCiAgc3RyX3NwbGl0KHN0cmluZyA9IC4scGF0dGVybiA9ICc7JykKbmFtZXMocHJvdGVpbklkc05vTUJSKSA8LSBkYXRhTm9NQlIkUHJvdGVpbgpnZW5lTmFtZXNOb01CUiA8LSBtYXAoLnggPSBwcm90ZWluSWRzTm9NQlIsLmYgPSB+Z2V0Qk0obWFydD1IU2FwaWVuc01hcnQsdmFsdWVzID0gLngsYXR0cmlidXRlcyA9ICd1bmlwcm90X2duX3N5bWJvbCcsZmlsdGVycyA9ICd1bmlwcm90c3dpc3Nwcm90JykpICU+JQogIG1hcCguLHVubGlzdCkKCk5vTUJSR2VuZU5hbWVzIDwtIGdlbmVOYW1lc05vTUJSICU+JSAKICBtYXAoLnggPSAuLC5mID0gfnBhc3RlMCgueCxjb2xsYXBzZSA9ICc7JykpICU+JSAKICB1bm5hbWUgJT4lIAogIHVubGlzdApkYXRhTm9NQlIgPC0gZGF0YU5vTUJSICU+JSAKICBtdXRhdGUoR2VuZT1Ob01CUkdlbmVOYW1lcywuYWZ0ZXI9UHJvdGVpbikKd3JpdGVfdHN2KHggPSBkYXRhTm9NQlIsZmlsZSA9ICdEREEvRGF0YUNvcnJlbGF0aW9uL25vTUJSL0REQV9ub01CUl9Qcm90ZWluX1JSb2xsdXAvRERBX25vTUJSX1JSb2x1cF9HZW5lcy50c3YnKQpgYGAKCgojIyMgTWF0Y2ggQmV0d2VlbiBSdW5zCgojIyMjIFByZXBhcmUgRmlsZXMgd2l0aCBzaG9ydCBuYW1lcwpgYGB7cn0KZGF0YVBNQlIgPC0gJ0REQS9EYXRhREUvTWF4UXVhbnQvTUJSL3NjYW5iX2FpbTJfdGhpc3AyX21ici9wZXB0aWRlcy50eHQnCmRhdGFNQlIgPC0gcmVhZF90c3YoZGF0YVBNQlIsY29sX25hbWVzID0gVFJVRSxndWVzc19tYXggPSAxMDAwMCkgJT4lIAogIGRwbHlyOjpzZWxlY3QoUHJvdGVpbnMsYExlYWRpbmcgcmF6b3IgcHJvdGVpbmAsU2VxdWVuY2UsY29udGFpbnMoJ0xGUScpKQpkYXRhTUJSUmVkdWNlZCA8LSBkYXRhTUJSICU+JQogIGRwbHlyOjpzZWxlY3QoY29udGFpbnMoJ0xGUScpKQpkYXRhTUJSTmFtZXMgPC0gZGF0YU1CUlJlZHVjZWQgJT4lIAogIGNvbG5hbWVzICU+JSAKICBzdHJfZXh0cmFjdChzdHJpbmcgPSAuLHBhdHRlcm4gPSAnQls6ZGlnaXQ6XS4rfFBvb2wuKicpICU+JSAKICBzdHJfcmVwbGFjZShzdHJpbmcgPSAuLHBhdHRlcm4gPSAnUG9vbC4qJyxyZXBsYWNlbWVudCA9ICdQb29sX0REQV8wMScpCmNvbG5hbWVzKGRhdGFNQlJSZWR1Y2VkKSA8LSBkYXRhTUJSTmFtZXMKZGF0YU1CUiA8LSBkYXRhTUJSICU+JSAKICB0cmFuc211dGUoUHJvdGVpbnM9UHJvdGVpbnMsTGVhZGluZy5SYXpvci5Qcm90ZWluPWBMZWFkaW5nIHJhem9yIHByb3RlaW5gLFNlcXVlbmNlPVNlcXVlbmNlLGRhdGFNQlJSZWR1Y2VkKQpkYXRhTUJSIDwtIGRhdGFNQlIgJT4lIAogIGZpbHRlcihzdHJfZGV0ZWN0KHN0cmluZyA9IFByb3RlaW5zLHBhdHRlcm4gPSAnQ09OXFxfJyxuZWdhdGUgPSBUUlVFKSkgJT4lIAogIGZpbHRlcihzdHJfZGV0ZWN0KHN0cmluZyA9IFByb3RlaW5zLHBhdHRlcm4gPSAnQ09OVEFNXFxfJyxuZWdhdGUgPSBUUlVFKSkKCndyaXRlX3Rzdih4ID0gZGF0YU1CUixmaWxlID0gJ0REQS9EYXRhREUvTWF4UXVhbnQvTUJSL3BlcHRpZGVzX21icl9zaG9ydG5hbWVzLnRzdicpCmBgYAoKIyMjIyBQZXB0aWRlLWxldmVsIE5vcm1hbGl6YXRpb24KYGBge3J9CmpvYk1CUiA8LSAnRERBX01CUl9wZXB0aWRlX25vcm1hbGlzYXRpb24nCmRhdGFQTUJSIDwtICdEREEvRGF0YURFL01heFF1YW50L01CUi9wZXB0aWRlc19tYnJfc2hvcnRuYW1lcy50c3YnCmRlc2lnblAgPC0gJ0REQS9EZXNpZ25GaWxlc0REQS9kZXNpZ25fYWltMl9jbGluaWNhbC50c3YnCm91dHB1dFBNQlIgPC0gJ0REQS9EYXRhREUvTWF4UXVhbnQvTUJSJwoKbm9ybWFseXplcihqb2JOYW1lID0gam9iTUJSLGRlc2lnblBhdGggPSBkZXNpZ25QLGRhdGFQYXRoID0gZGF0YVBNQlIsb3V0cHV0RGlyID0gb3V0cHV0UE1CUixyZXF1aXJlUmVwbGljYXRlcyA9IEZBTFNFLG5vcm1hbGl6ZVJldGVudGlvblRpbWUgPSBGQUxTRSx6ZXJvVG9OQSA9IFRSVUUsc2FtcGxlQ29sTmFtZSA9ICdQb3NpdGlvbicsZ3JvdXBDb2xOYW1lID0gJ2dyb3VwJykKYGBgCgojIyMjIFNvcnQgUGVwdGlkZXMgQWxwaGFiZXRpY2FsbHkKYGBge3J9CnNvcnRJZHMgPC0gZnVuY3Rpb24oZGF0YSxwcm90ZWluQ29sdW1uID0gJ1Byb3RlaW5zJywuLi4pewogIGRhdGEgPC0gcHVsbChkYXRhLHByb3RlaW5Db2x1bW4pICU+JSAKICAgIHN0cl9zcGxpdChzdHJpbmcgPSAuLHBhdHRlcm4gPSAnOycpICU+JSAKICAgIG1hcCgueCA9IC4sLmYgPSBzdHJfc29ydCkgJT4lIAogICAgbWFwKC54ID0gLiwuZiA9IGZ1bmN0aW9uKHgpIHBhc3RlMCh4LGNvbGxhcHNlID0gJzsnKSkgJT4lIAogICAgdW5saXN0ICU+JSAKICAgIG11dGF0ZShkYXRhLFByb3RlaW4uSUQ9LiwuYmVmb3JlPWNvbG5hbWVzKGRhdGFbMl0pKSAlPiUgCiAgICBkcGx5cjo6c2VsZWN0KC1wcm90ZWluQ29sdW1uKQogIG5hbWVzKGRhdGEpW25hbWVzKGRhdGEpPT0nUHJvdGVpbi5JRCddIDwtICdQcm90ZWluJwogIHJldHVybihkYXRhKQp9CmBgYApgYGB7cn0KZGF0YU1CUiA8LSByZWFkX3RzdihmaWxlID0gJ0REQS9EYXRhREUvTWF4UXVhbnQvTUJSL0REQV9NQlJfcGVwdGlkZV9ub3JtYWxpc2F0aW9uL0N5Y0xvZXNzLW5vcm1hbGl6ZWQudHh0JykgJT4lIAogIHNvcnRJZHMoZGF0YSA9IC4scHJvdGVpbkNvbHVtbiA9ICdQcm90ZWlucycpCgp3cml0ZV90c3YoeCA9IGRhdGFNQlIsZmlsZSA9ICdEREEvRGF0YURFL01heFF1YW50L01CUi9EREFfTUJSX3BlcHRpZGVfbm9ybWFsaXNhdGlvbi9DeWNMb2Vzc19ub3JtX3NvcnRlZC50c3YnKQpgYGAKCiMjIyMgUHJvdGVpbiBSb2xsdXAKYGBge2Jhc2h9ClJzY3JpcHQgfi9TQ0FOQl9QVUJMSUNBVElPTi9BbGdvcml0aG1zL1Byb3RlaW5Sb2xsdXAtbWFzdGVyL1IvcHJvdGVpbl9yb2xsdXAuUiBcCiAgICAtLXJkZl9mcCB+L1NDQU5CX1BVQkxJQ0FUSU9OL0REQS9EYXRhREUvTWF4UXVhbnQvTUJSL0REQV9NQlJfcGVwdGlkZV9ub3JtYWxpc2F0aW9uL0N5Y0xvZXNzX25vcm1fc29ydGVkLnRzdiBcCiAgICAtLWRkZl9mcCB+L1NDQU5CX1BVQkxJQ0FUSU9OL0REQS9EZXNpZ25GaWxlc0REQS9kZXNpZ25fYWltMl9jbGluaWNhbC50c3YgXAogICAgLS1zYW1wbGVfY29sIFBvc2l0aW9uIFwKICAgIC0tcHJvdGVpbl9jb2wgUHJvdGVpbiBcCiAgICAtLW91dF9mcCB+L1NDQU5CX1BVQkxJQ0FUSU9OL0REQS9EYXRhREUvTWF4UXVhbnQvTUJSL0REQV9NQlJfUHJvdGVpbl9SUm9sbHVwL0REQV9NQlJfUlJvbGx1cC50c3YKYGBgCgojIyMgTm8gTWF0Y2ggQmV0d2VlbiBSdW5zCgojIyMjIFByZXBhcmUgRmlsZXMgd2l0aCBzaG9ydCBuYW1lcwpgYGB7cn0KZGF0YVBub01CUiA8LSAnRERBL0RhdGFERS9NYXhRdWFudC9Ob01CUi9zY2FuYl9haW0yX3RoaXNwMl9ub19tYnIvcGVwdGlkZXMudHh0JwpkYXRhTm9NQlIgPC0gcmVhZF90c3YoZGF0YVBub01CUixjb2xfbmFtZXMgPSBUUlVFLGd1ZXNzX21heCA9IDEwMDAwKSAlPiUgCiAgZHBseXI6OnNlbGVjdChQcm90ZWlucyxgTGVhZGluZyByYXpvciBwcm90ZWluYCxTZXF1ZW5jZSxjb250YWlucygnTEZRJykpCmRhdGFOb01CUlJlZHVjZWQgPC0gZGF0YU5vTUJSICU+JQogIGRwbHlyOjpzZWxlY3QoY29udGFpbnMoJ0xGUScpKQpkYXRhTm9NQlJOYW1lcyA8LSBkYXRhTm9NQlJSZWR1Y2VkICU+JSAKICBjb2xuYW1lcyAlPiUgCiAgc3RyX2V4dHJhY3Qoc3RyaW5nID0gLixwYXR0ZXJuID0gJ0JbOmRpZ2l0Ol0uK3xQb29sLionKSAlPiUgCiAgc3RyX3JlcGxhY2Uoc3RyaW5nID0gLixwYXR0ZXJuID0gJ1Bvb2wuKicscmVwbGFjZW1lbnQgPSAnUG9vbF9EREFfMDEnKQpjb2xuYW1lcyhkYXRhTm9NQlJSZWR1Y2VkKSA8LSBkYXRhTm9NQlJOYW1lcwpkYXRhTm9NQlIgPC0gZGF0YU5vTUJSICU+JSAKICB0cmFuc211dGUoUHJvdGVpbnM9UHJvdGVpbnMsTGVhZGluZy5SYXpvci5Qcm90ZWluPWBMZWFkaW5nIHJhem9yIHByb3RlaW5gLFNlcXVlbmNlPVNlcXVlbmNlLGRhdGFOb01CUlJlZHVjZWQpCmRhdGFOb01CUiA8LSBkYXRhTm9NQlIgJT4lIAogIGZpbHRlcihzdHJfZGV0ZWN0KHN0cmluZyA9IFByb3RlaW5zLHBhdHRlcm4gPSAnQ09OXFxfJyxuZWdhdGUgPSBUUlVFKSkgJT4lIAogIGZpbHRlcihzdHJfZGV0ZWN0KHN0cmluZyA9IFByb3RlaW5zLHBhdHRlcm4gPSAnQ09OVEFNXFxfJyxuZWdhdGUgPSBUUlVFKSkKCndyaXRlX3Rzdih4ID0gZGF0YU5vTUJSLGZpbGUgPSAnRERBL0RhdGFERS9NYXhRdWFudC9Ob01CUi9wZXB0aWRlc19ub21icl9zaG9ydG5hbWVzLnRzdicpCmBgYAoKIyMjIyBQZXB0aWRlLWxldmVsIE5vcm1hbGl6YXRpb24KYGBge3J9CmpvYk5vTUJSIDwtICdEREFfbm9fTUJSX3BlcHRpZGVfbm9ybWFsaXNhdGlvbicKZGF0YVBOb01CUiA8LSAnRERBL0RhdGFERS9NYXhRdWFudC9Ob01CUi9wZXB0aWRlc19ub21icl9zaG9ydG5hbWVzLnRzdicKZGVzaWduUCA8LSAnRERBL0Rlc2lnbkZpbGVzRERBL2Rlc2lnbl9haW0yX2NsaW5pY2FsLnRzdicKb3V0cHV0UE5vTUJSIDwtICdEREEvRGF0YURFL01heFF1YW50L05vTUJSJwoKbm9ybWFseXplcihqb2JOYW1lID0gam9iTm9NQlIsZGVzaWduUGF0aCA9IGRlc2lnblAsZGF0YVBhdGggPSBkYXRhUE5vTUJSLG91dHB1dERpciA9IG91dHB1dFBOb01CUixyZXF1aXJlUmVwbGljYXRlcyA9IEZBTFNFLG5vcm1hbGl6ZVJldGVudGlvblRpbWUgPSBGQUxTRSx6ZXJvVG9OQSA9IFRSVUUsc2FtcGxlQ29sTmFtZSA9ICdQb3NpdGlvbicsZ3JvdXBDb2xOYW1lID0gJ2dyb3VwJykKYGBgCgojIyMjIFNvcnQgUGVwdGlkZXMgQWxwaGFiZXRpY2FsbHkKYGBge3J9CnNvcnRJZHMgPC0gZnVuY3Rpb24oZGF0YSxwcm90ZWluQ29sdW1uID0gJ1Byb3RlaW5zJywuLi4pewogIGRhdGEgPC0gcHVsbChkYXRhLHByb3RlaW5Db2x1bW4pICU+JSAKICAgIHN0cl9zcGxpdChzdHJpbmcgPSAuLHBhdHRlcm4gPSAnOycpICU+JSAKICAgIG1hcCgueCA9IC4sLmYgPSBzdHJfc29ydCkgJT4lIAogICAgbWFwKC54ID0gLiwuZiA9IGZ1bmN0aW9uKHgpIHBhc3RlMCh4LGNvbGxhcHNlID0gJzsnKSkgJT4lIAogICAgdW5saXN0ICU+JSAKICAgIG11dGF0ZShkYXRhLFByb3RlaW4uSUQ9LiwuYmVmb3JlPWNvbG5hbWVzKGRhdGFbMl0pKSAlPiUgCiAgICBkcGx5cjo6c2VsZWN0KC1wcm90ZWluQ29sdW1uKQogIG5hbWVzKGRhdGEpW25hbWVzKGRhdGEpPT0nUHJvdGVpbi5JRCddIDwtICdQcm90ZWluJwogIHJldHVybihkYXRhKQp9CmBgYApgYGB7cn0KZGF0YU5vTUJSIDwtIHJlYWRfdHN2KGZpbGUgPSAnRERBL0RhdGFERS9NYXhRdWFudC9Ob01CUi9EREFfbm9fTUJSX3BlcHRpZGVfbm9ybWFsaXNhdGlvbi9DeWNMb2Vzcy1ub3JtYWxpemVkLnR4dCcpICU+JSAKICBzb3J0SWRzKGRhdGEgPSAuLHByb3RlaW5Db2x1bW4gPSAnUHJvdGVpbnMnKQoKd3JpdGVfdHN2KHggPSBkYXRhTm9NQlIsZmlsZSA9ICdEREEvRGF0YURFL01heFF1YW50L05vTUJSL0REQV9ub19NQlJfcGVwdGlkZV9ub3JtYWxpc2F0aW9uL0N5Y0xvZXNzX25vcm1fc29ydGVkLnRzdicpCmBgYAoKIyMjIyBQcm90ZWluIFJvbGx1cApgYGB7YmFzaH0KUnNjcmlwdCB+L1NDQU5CX1BVQkxJQ0FUSU9OL0FsZ29yaXRobXMvUHJvdGVpblJvbGx1cC1tYXN0ZXIvUi9wcm90ZWluX3JvbGx1cC5SIFwKICAgIC0tcmRmX2ZwIH4vU0NBTkJfUFVCTElDQVRJT04vRERBL0RhdGFERS9NYXhRdWFudC9Ob01CUi9EREFfbm9fTUJSX3BlcHRpZGVfbm9ybWFsaXNhdGlvbi9DeWNMb2Vzc19ub3JtX3NvcnRlZC50c3YgXAogICAgLS1kZGZfZnAgfi9TQ0FOQl9QVUJMSUNBVElPTi9EREEvRGVzaWduRmlsZXNEREEvZGVzaWduX2FpbTJfY2xpbmljYWwudHN2IFwKICAgIC0tc2FtcGxlX2NvbCBQb3NpdGlvbiBcCiAgICAtLXByb3RlaW5fY29sIFByb3RlaW4gXAogICAgLS1vdXRfZnAgfi9TQ0FOQl9QVUJMSUNBVElPTi9EREEvRGF0YURFL01heFF1YW50L05vTUJSL0REQV9ub01CUl9Qcm90ZWluX1JSb2xsdXAvRERBX25vTUJSX1JSb2xsdXAudHN2CmBgYAoKCiMjIFJOQS1TZXEKCiMjIyBQcmVwcm9jZXNzaW5nCiAgVGhpcyBwb3J0aW9uIGlzIHJ1biBpbiBvcmRlciB0byBnZW5lcmF0ZSBzbWFsbGVyIGRhdGEgZmlsZXMgY29udGFpbmluZyBvbmx5IFJOQS1zZXEgc2FtcGxlcyB0aGF0IG1hdGNoZXMgdGhvc2UgcHJlc2VudCBpbiBEREEgYW5kIERJQSBhbmFseXNlcy4KYGBge3J9CnJuYURhdGEgPC0gcmVhZF9jc3YoZmlsZSA9ICdSTkEtc2VxL0dTRTk2MDU4L0dTRTk2MDU4X2dlbmVfZXhwcmVzc2lvbl8zMjczX3NhbXBsZXNfYW5kXzEzNl9yZXBsaWNhdGVzX3RyYW5zZm9ybWVkLmNzdicpClJOQU5hbWVzIDwtIGNvbG5hbWVzKHJuYURhdGEpICU+JSAKICBzdHJfcmVwbGFjZShzdHJpbmcgPSAuLHBhdHRlcm4gPSAnXFwuLi4xJyxyZXBsYWNlbWVudCA9ICdHZW5lJykKY29sbmFtZXMocm5hRGF0YSkgPC0gUk5BTmFtZXMKCmRlc2lnblJOQSA8LSByZWFkX3RzdignUk5BLXNlcS9kZXNpZ25STkEudHN2JykKCnJuYURhdGFSZWR1Y2VkIDwtIHJuYURhdGEgJT4lIAogIGRwbHlyOjpzZWxlY3QoR2VuZSxkZXNpZ25STkEkU2FtcGxlX3RpdGxlKQp3cml0ZV90c3YoeCA9IHJuYURhdGFSZWR1Y2VkLGZpbGUgPSAnUk5BLXNlcS9HU0U5NjA1OF9nZW5lX2V4cHJlc3Npb25fUmVkdWNlZC50c3YnKQpgYGAKCgojIENvcnJlbGF0aW9ucwoKIyMgRnVuY3Rpb24KYGBge3J9CmNvcnJlbGF0aW9uX2RhdGFzZXRzIDwtIGZ1bmN0aW9uKGRhdGExLGRhdGEyLC4uLil7CiAgcmVxdWlyZShwc3ljaCkKICBkYXRhMSA8LSB0KGRhdGExKQogIGRhdGEyIDwtIHQoZGF0YTIpCiAgc3BlYXJtYW5fY29ycmVsYXRpb24gPC0gbWFwKC54ID0gMTpuY29sKGRhdGExKSwuZiA9IGZ1bmN0aW9uKGkpIGNvcnIudGVzdChkYXRhMVssaV0sZGF0YTJbLGldLG1ldGhvZD0nc3BlYXJtYW4nLGNpPUZBTFNFLGFkanVzdCA9ICdub25lJykpCiAgcGVhcnNvbl9jb3JyZWxhdGlvbiA8LSBtYXAoLnggPSAxOm5jb2woZGF0YTEpLC5mID0gZnVuY3Rpb24oaSkgY29yci50ZXN0KGRhdGExWyxpXSxkYXRhMlssaV0sbWV0aG9kPSdwZWFyc29uJyxjaT1GQUxTRSxhZGp1c3QgPSAnbm9uZScpKQogIHNwZWFybWFuX3IgPC0gbWFwKHNwZWFybWFuX2NvcnJlbGF0aW9uLH5wbHVjaygueCwncicpKSAlPiUgdW5saXN0KC4pCiAgc3BlYXJtYW5fcCA8LSBtYXAoc3BlYXJtYW5fY29ycmVsYXRpb24sfnBsdWNrKC54LCdwJykpICU+JSB1bmxpc3QoLikKICBwZWFyc29uX3IgPC0gbWFwKHBlYXJzb25fY29ycmVsYXRpb24sfnBsdWNrKC54LCdyJykpICU+JSB1bmxpc3QoLikKICBwZWFyc29uX3AgPC0gbWFwKHBlYXJzb25fY29ycmVsYXRpb24sfnBsdWNrKC54LCdwJykpICU+JSB1bmxpc3QoLikKICByZXN1bHRzIDwtIHRpYmJsZShTcGVhcm1hbl9TY29yZSA9IHNwZWFybWFuX3IscC52YWx1ZS5zcGVhcm1hbiA9IHNwZWFybWFuX3AsUGVhcnNvbl9TY29yZSA9IHBlYXJzb25fcixwLnZhbHVlLnBlYXJzb24gPSBwZWFyc29uX3ApCiAgcmV0dXJuKHJlc3VsdHMpCn0KYGBgCgojIyBPdmVyYWxsCgojIyMgRW5jeWNsb3BlRElBIHZzIFJOQS1zZXEKYGBge3J9CmRpYVAgPC0gJ0RJQS9EYXRhREUvRW5jeWNsb3BlRElBL0RJQV9FbmN5Y2xvcGVESUFfUHJvdGVpbl9SUm9sdXAvRElBX0VuY3ljbG9wZURJQV9SUm9sbHVwX0dlbmVzLnRzdicKZGVzaWduUCA8LSAnUk5BLXNlcS9kZXNpZ25STkEudHN2JwpybmFESUEgPC0gJ1JOQS1zZXEvR1NFOTYwNThfZ2VuZV9leHByZXNzaW9uX1JlZHVjZWQudHN2JyAlPiUgCiAgcmVhZF90c3YKCmRlc2lnbiA8LSByZWFkX3RzdihkZXNpZ25QKSAlPiUgCiAgZmlsdGVyKCFpcy5uYShTYW1wbGVfdGl0bGUpKQpkYXRhRElBIDwtIHJlYWRfdHN2KGRpYVApICU+JSAKICBkcGx5cjo6c2VsZWN0KDE6MyxHZW5lLGRlc2lnbiRQb3NpdGlvbikKCm1lcmdlZERhdGEgPC0gaW5uZXJfam9pbihkYXRhRElBLHJuYURJQSwiR2VuZSIpCgpkaWFEYXRhUmVkdWNlZCA8LSBtZXJnZWREYXRhICU+JSAKICBkcGx5cjo6c2VsZWN0KGRlc2lnbiRQb3NpdGlvbikKcm5hRGF0YVJlZHVjZWQgPC0gbWVyZ2VkRGF0YSAlPiUgCiAgZHBseXI6OnNlbGVjdChkZXNpZ24kU2FtcGxlX3RpdGxlKQoKY29ycmVsYXRpb25SbmFFbmN5Y2xvcGVkaWEgPC0gY29ycmVsYXRpb25fZGF0YXNldHMoZGlhRGF0YVJlZHVjZWQscm5hRGF0YVJlZHVjZWQpICU+JSAKICBtdXRhdGUoLixQcm90ZWluID0gbWVyZ2VkRGF0YSRQcm90ZWluLCBHZW5lPW1lcmdlZERhdGEkR2VuZSwgUC5QZXJhc29uLkFkaj1wLmFkanVzdChwLnZhbHVlLnBlYXJzb24sbWV0aG9kID0gJ2ZkcicpLFAuU3BlYXJtYW4uQWRqPXAuYWRqdXN0KHAudmFsdWUuc3BlYXJtYW4sbWV0aG9kID0gJ2ZkcicpKQp3cml0ZV90c3YoeCA9IGNvcnJlbGF0aW9uUm5hRW5jeWNsb3BlZGlhLGZpbGUgPSAnQ29ycmVsYXRpb25zL2NvcnJlbGF0aW9uX1JOQV9FbmN5Y2xvcGVESUEudHN2JykKYGBgCgojIyMgRElBLU5OIHZzIFJOQS1zZXEKYGBge3J9CmRpYVAgPC0gJ0RJQS9EYXRhREUvRElBTk4vRElBX0RJQU5OX1Byb3RlaW5fUlJvbGx1cC9ESUFfRElBTk5fUlJvbGx1cF9HZW5lcy50c3YnCmRlc2lnblAgPC0gJ1JOQS1zZXEvZGVzaWduUk5BLnRzdicKcm5hRElBIDwtICdSTkEtc2VxL0dTRTk2MDU4X2dlbmVfZXhwcmVzc2lvbl9SZWR1Y2VkLnRzdicgJT4lIAogIHJlYWRfdHN2CgpkZXNpZ24gPC0gcmVhZF90c3YoZGVzaWduUCkgJT4lIAogIGZpbHRlcighaXMubmEoU2FtcGxlX3RpdGxlKSkKZGF0YURJQSA8LSByZWFkX3RzdihkaWFQKSAlPiUgCiAgZHBseXI6OnNlbGVjdCgxOjMsR2VuZT1HZW5lcyxkZXNpZ24kUG9zaXRpb24pCgptZXJnZWREYXRhIDwtIGlubmVyX2pvaW4oZGF0YURJQSxybmFESUEsIkdlbmUiKQoKZGlhRGF0YVJlZHVjZWQgPC0gbWVyZ2VkRGF0YSAlPiUgCiAgZHBseXI6OnNlbGVjdChkZXNpZ24kUG9zaXRpb24pCnJuYURhdGFSZWR1Y2VkIDwtIG1lcmdlZERhdGEgJT4lIAogIGRwbHlyOjpzZWxlY3QoZGVzaWduJFNhbXBsZV90aXRsZSkKCmNvcnJlbGF0aW9uUm5hRElBTk4gPC0gY29ycmVsYXRpb25fZGF0YXNldHMoZGlhRGF0YVJlZHVjZWQscm5hRGF0YVJlZHVjZWQpICU+JSAKICBtdXRhdGUoLixQcm90ZWluID0gbWVyZ2VkRGF0YSRQcm90ZWluLCBHZW5lPW1lcmdlZERhdGEkR2VuZSwgUC5QZXJhc29uLkFkaj1wLmFkanVzdChwLnZhbHVlLnBlYXJzb24sbWV0aG9kID0gJ2ZkcicpLFAuU3BlYXJtYW4uQWRqPXAuYWRqdXN0KHAudmFsdWUuc3BlYXJtYW4sbWV0aG9kID0gJ2ZkcicpKQp3cml0ZV90c3YoeCA9IGNvcnJlbGF0aW9uUm5hRElBTk4sZmlsZSA9ICdDb3JyZWxhdGlvbnMvY29ycmVsYXRpb25fUk5BX0RJQU5OLnRzdicpCmBgYAoKIyMjIEREQSBNYXRjaCBCZXR3ZWVuIFJ1bnMgdnMgUk5BLXNlcQpgYGB7cn0KZGRhUCA8LSAnRERBL0RhdGFDb3JyZWxhdGlvbi9NQlIvRERBX01CUl9Qcm90ZWluX1JvbGx1cC9EREFfTUJSX1JSb2xsdXBfR2VuZXMudHN2JwpkZXNpZ25QIDwtICdSTkEtc2VxL2Rlc2lnblJOQS50c3YnCnJuYUREQSA8LSAnUk5BLXNlcS9HU0U5NjA1OF9nZW5lX2V4cHJlc3Npb25fUmVkdWNlZC50c3YnICU+JSAKICByZWFkX3RzdgoKZGVzaWduIDwtIHJlYWRfdHN2KGRlc2lnblApICU+JSAKICBmaWx0ZXIoIWlzLm5hKFNhbXBsZV90aXRsZSkpCmRhdGFEREEgPC0gcmVhZF90c3YoZGRhUCkgJT4lIAogIGRwbHlyOjpzZWxlY3QoMTozLEdlbmUsZGVzaWduJFBvc2l0aW9uKQoKbWVyZ2VkRGF0YSA8LSBpbm5lcl9qb2luKGRhdGFEREEscm5hRERBLCJHZW5lIikKCmRkYURhdGFSZWR1Y2VkIDwtIG1lcmdlZERhdGEgJT4lIAogIGRwbHlyOjpzZWxlY3QoZGVzaWduJFBvc2l0aW9uKQpybmFEYXRhUmVkdWNlZCA8LSBtZXJnZWREYXRhICU+JSAKICBkcGx5cjo6c2VsZWN0KGRlc2lnbiRTYW1wbGVfdGl0bGUpCgpjb3JyZWxhdGlvblJuYUREQU1CUiA8LSBjb3JyZWxhdGlvbl9kYXRhc2V0cyhkZGFEYXRhUmVkdWNlZCxybmFEYXRhUmVkdWNlZCkgJT4lIAogIG11dGF0ZSguLFByb3RlaW4gPSBtZXJnZWREYXRhJFByb3RlaW4sIEdlbmU9bWVyZ2VkRGF0YSRHZW5lLCBQLlBlcmFzb24uQWRqPXAuYWRqdXN0KHAudmFsdWUucGVhcnNvbixtZXRob2QgPSAnZmRyJyksUC5TcGVhcm1hbi5BZGo9cC5hZGp1c3QocC52YWx1ZS5zcGVhcm1hbixtZXRob2QgPSAnZmRyJykpCndyaXRlX3Rzdih4ID0gY29ycmVsYXRpb25SbmFEREFNQlIsZmlsZSA9ICdDb3JyZWxhdGlvbnMvY29ycmVsYXRpb25fUk5BX0REQV9NQlIudHN2JykKYGBgCgojIyMgRERBIG5vIE1hdGNoIEJldHdlZW4gUnVucyB2cyBSTkEtc2VxCmBgYHtyfQpkZGFQIDwtICdEREEvRGF0YUNvcnJlbGF0aW9uL25vTUJSL0REQV9ub01CUl9Qcm90ZWluX1JSb2xsdXAvRERBX25vTUJSX1JSb2x1cF9HZW5lcy50c3YnCmRlc2lnblAgPC0gJ1JOQS1zZXEvZGVzaWduUk5BLnRzdicKcm5hRERBIDwtICdSTkEtc2VxL0dTRTk2MDU4X2dlbmVfZXhwcmVzc2lvbl9SZWR1Y2VkLnRzdicgJT4lIAogIHJlYWRfdHN2CgpkZXNpZ24gPC0gcmVhZF90c3YoZGVzaWduUCkgJT4lIAogIGZpbHRlcighaXMubmEoU2FtcGxlX3RpdGxlKSkKZGF0YUREQSA8LSByZWFkX3RzdihkZGFQKSAlPiUgCiAgZHBseXI6OnNlbGVjdCgxOjMsR2VuZSxkZXNpZ24kUG9zaXRpb24pCgptZXJnZWREYXRhIDwtIGlubmVyX2pvaW4oZGF0YUREQSxybmFEREEsIkdlbmUiKQoKZGRhRGF0YVJlZHVjZWQgPC0gbWVyZ2VkRGF0YSAlPiUgCiAgZHBseXI6OnNlbGVjdChkZXNpZ24kUG9zaXRpb24pCnJuYURhdGFSZWR1Y2VkIDwtIG1lcmdlZERhdGEgJT4lIAogIGRwbHlyOjpzZWxlY3QoZGVzaWduJFNhbXBsZV90aXRsZSkKCmNvcnJlbGF0aW9uUm5hRERBTm9NQlIgPC0gY29ycmVsYXRpb25fZGF0YXNldHMoZGRhRGF0YVJlZHVjZWQscm5hRGF0YVJlZHVjZWQpICU+JQogIG11dGF0ZSguLFByb3RlaW4gPSBtZXJnZWREYXRhJFByb3RlaW4sIEdlbmU9bWVyZ2VkRGF0YSRHZW5lLCBQLlBlcmFzb24uQWRqPXAuYWRqdXN0KHAudmFsdWUucGVhcnNvbixtZXRob2QgPSAnZmRyJyksUC5TcGVhcm1hbi5BZGo9cC5hZGp1c3QocC52YWx1ZS5zcGVhcm1hbixtZXRob2QgPSAnZmRyJykpCndyaXRlX3Rzdih4ID0gY29ycmVsYXRpb25SbmFEREFOb01CUixmaWxlID0gJ0NvcnJlbGF0aW9ucy9jb3JyZWxhdGlvbl9STkFfRERBX05vTUJSLnRzdicpCmBgYAoKIyMgQWZ0ZXIgbG93IHZhcmlhbmNlIHJlbW92YWwKCiMjIyBGdW5jdGlvbgpgYGB7cn0KbWVkaWFuRmlsdGVyaW5nUHJvdFJOQSA8LSBmdW5jdGlvbihwcm90RGF0YSxybmFEYXRhLGRlc2lnbiwuLi4pewogIHZhcmlhbmNlTkEgPC0gZnVuY3Rpb24oZGF0YSkgdmFyKGRhdGEsbmEucm09VFJVRSkKCiAgbWVyZ2VkRGF0YSA8LSBpbm5lcl9qb2luKHByb3REYXRhLHJuYURhdGEsJ0dlbmUnKQogIAogIHZhcmlhbmNlUHJvdCA8LSBtZXJnZWREYXRhICU+JSAKICAgIGRwbHlyOjpzZWxlY3QoZGVzaWduJFBvc2l0aW9uKSAlPiUgCiAgICB0ICU+JSAKICAgIGFzX3RpYmJsZSAlPiUgCiAgICBtYXBfZGZjKC4sdmFyaWFuY2VOQSkgJT4lIAogICAgdAogICAgCiAgdmFyaWFuY2VSTkEgPC0gbWVyZ2VkRGF0YSAlPiUgCiAgICBkcGx5cjo6c2VsZWN0KGRlc2lnbiRTYW1wbGVfdGl0bGUpICU+JSAKICAgIHQgJT4lIAogICAgYXNfdGliYmxlICU+JSAKICAgIG1hcF9kZmMoLix2YXJpYW5jZU5BKSAlPiUgCiAgICB0CgogIG1lcmdlZERhdGEgPC0gbWVyZ2VkRGF0YSAlPiUgCiAgICBtdXRhdGUoLixWYXJQcm90PXZhcmlhbmNlUHJvdCxWYXJSTkE9dmFyaWFuY2VSTkEpCiAgCiAgbG93VmFyRGF0YSA8LSBtZXJnZWREYXRhICU+JSAKICAgIGZpbHRlcihWYXJQcm90ID4gbWVkaWFuKFZhclByb3QsbmEucm0gPSBUKSxWYXJSTkEgPiBtZWRpYW4oVmFyUk5BLG5hLnJtID0gVCkpCiAgCiAgbG93VmFyUHJvdCA8LSBsb3dWYXJEYXRhICU+JSAKICAgIGRwbHlyOjpzZWxlY3QoZGVzaWduJFBvc2l0aW9uKQogIGxvd1ZhclJOQSA8LSBsb3dWYXJEYXRhICU+JSAKICAgIGRwbHlyOjpzZWxlY3QoZGVzaWduJFNhbXBsZV90aXRsZSkKICAKICBjb3JyZWxhdGlvbkxvd1ZhckZpbHRlcmluZyA8LSBjb3JyZWxhdGlvbl9kYXRhc2V0cyhkYXRhMSA9IGxvd1ZhclByb3QsZGF0YTIgPSBsb3dWYXJSTkEpICU+JQogICAgbXV0YXRlKC4sR2VuZSA9IGxvd1ZhckRhdGEkR2VuZSwgUC5QZXJhc29uLkFkaj1wLmFkanVzdChwLnZhbHVlLnBlYXJzb24sbWV0aG9kID0gJ2ZkcicpLFAuU3BlYXJtYW4uQWRqPXAuYWRqdXN0KHAudmFsdWUuc3BlYXJtYW4sbWV0aG9kID0gJ2ZkcicpKQogIAogIHJlc3VsdHMgPC0gbGlzdChsb3dWYXI9Y29ycmVsYXRpb25Mb3dWYXJGaWx0ZXJpbmcpCiAgcmV0dXJuKHJlc3VsdHMpCn0KYGBgCgojIyMgRW5jeWNsb3BlRElBIHZzIFJOQS1zZXEKYGBge3J9CmRpYVAgPC0gJ0RJQS9EYXRhREUvRW5jeWNsb3BlRElBL0RJQV9FbmN5Y2xvcGVESUFfUHJvdGVpbl9SUm9sdXAvRElBX0VuY3ljbG9wZURJQV9SUm9sbHVwX0dlbmVzLnRzdicKZGVzaWduUCA8LSAnUk5BLXNlcS9kZXNpZ25STkEudHN2JwpybmFESUEgPC0gJ1JOQS1zZXEvR1NFOTYwNThfZ2VuZV9leHByZXNzaW9uX1JlZHVjZWQudHN2JyAlPiUgCiAgcmVhZF90c3YKCmRlc2lnbiA8LSByZWFkX3RzdihkZXNpZ25QKSAlPiUgCiAgZmlsdGVyKCFpcy5uYShTYW1wbGVfdGl0bGUpKQpkYXRhRElBIDwtIHJlYWRfdHN2KGRpYVApICU+JSAKICBkcGx5cjo6c2VsZWN0KDE6MyxHZW5lLGRlc2lnbiRQb3NpdGlvbikKCmNvcnJlbGF0aW9uRmlsdGVyRW5jeWNsb3BlZGlhUk5BIDwtIG1lZGlhbkZpbHRlcmluZ1Byb3RSTkEocHJvdERhdGEgPSBkYXRhRElBLHJuYURhdGEgPSBybmFESUEsZGVzaWduID0gZGVzaWduKQp3cml0ZV90c3YoeCA9IGNvcnJlbGF0aW9uRmlsdGVyRW5jeWNsb3BlZGlhUk5BJGxvd1ZhcixmaWxlID0gJ0NvcnJlbGF0aW9ucy9jb3JyZWxhdGlvbl9STkFfRW5jeWNsb3BlRElBX0xvd1Zhci50c3YnKQpgYGAKCiMjIyBESUEtTk4gdnMgUk5BLXNlcQpgYGB7cn0KZGlhUCA8LSAnRElBL0RhdGFERS9ESUFOTi9ESUFfRElBTk5fUHJvdGVpbl9SUm9sbHVwL0RJQV9ESUFOTl9SUm9sbHVwX0dlbmVzLnRzdicKZGVzaWduUCA8LSAnUk5BLXNlcS9kZXNpZ25STkEudHN2JwpybmFESUEgPC0gJ1JOQS1zZXEvR1NFOTYwNThfZ2VuZV9leHByZXNzaW9uX1JlZHVjZWQudHN2JyAlPiUgCiAgcmVhZF90c3YKCmRlc2lnbiA8LSByZWFkX3RzdihkZXNpZ25QKSAlPiUgCiAgZmlsdGVyKCFpcy5uYShTYW1wbGVfdGl0bGUpKQpkYXRhRElBIDwtIHJlYWRfdHN2KGRpYVApICU+JSAKICBkcGx5cjo6c2VsZWN0KDE6MyxHZW5lPUdlbmVzLGRlc2lnbiRQb3NpdGlvbikKCmNvcnJlbGF0aW9uRmlsdGVyRElBTk5STkEgPC0gbWVkaWFuRmlsdGVyaW5nUHJvdFJOQShwcm90RGF0YSA9IGRhdGFESUEscm5hRGF0YSA9IHJuYURJQSxkZXNpZ24gPSBkZXNpZ24pCndyaXRlX3Rzdih4ID0gY29ycmVsYXRpb25GaWx0ZXJESUFOTlJOQSRsb3dWYXIsZmlsZSA9ICdDb3JyZWxhdGlvbnMvY29ycmVsYXRpb25fRElBTk5fUk5BX0xvd1Zhci50c3YnKQpgYGAKCiMjIyBEREEgTWF0Y2ggQmV0d2VlbiBSdW5zIHZzIFJOQS1zZXEKYGBge3J9CmRkYVAgPC0gJ0REQS9EYXRhQ29ycmVsYXRpb24vTUJSL0REQV9NQlJfUHJvdGVpbl9Sb2xsdXAvRERBX01CUl9SUm9sbHVwX0dlbmVzLnRzdicKZGVzaWduUCA8LSdSTkEtc2VxL2Rlc2lnblJOQS50c3YnCnJuYUREQSA8LSAnUk5BLXNlcS9HU0U5NjA1OF9nZW5lX2V4cHJlc3Npb25fUmVkdWNlZC50c3YnICU+JSAKICByZWFkX3RzdgoKZGVzaWduIDwtIHJlYWRfdHN2KGRlc2lnblApICU+JSAKICBmaWx0ZXIoIWlzLm5hKFNhbXBsZV90aXRsZSkpCmRhdGFEREEgPC0gcmVhZF90c3YoZGRhUCkgJT4lIAogIGRwbHlyOjpzZWxlY3QoMTozLEdlbmUsZGVzaWduJFBvc2l0aW9uKQoKY29ycmVsYXRpb25SbmFEREFNQlIgPC0gbWVkaWFuRmlsdGVyaW5nUHJvdFJOQShwcm90RGF0YSA9IGRhdGFEREEscm5hRGF0YSA9IHJuYUREQSxkZXNpZ24gPSBkZXNpZ24pCndyaXRlX3Rzdih4ID0gY29ycmVsYXRpb25SbmFEREFNQlIkbG93VmFyLGZpbGUgPSAnQ29ycmVsYXRpb25zL2NvcnJlbGF0aW9uX1JOQV9EREFfTUJSX0xvd1Zhci50c3YnKQpgYGAKCiMjIyBEREEgbm8gTWF0Y2ggQmV0d2VlbiBSdW5zIHZzIFJOQS1zZXEKYGBge3J9CmRkYVAgPC0gJ0REQS9EYXRhQ29ycmVsYXRpb24vbm9NQlIvRERBX25vTUJSX1Byb3RlaW5fUlJvbGx1cC9EREFfbm9NQlJfUlJvbHVwX0dlbmVzLnRzdicKZGVzaWduUCA8LSAnUk5BLXNlcS9kZXNpZ25STkEudHN2JwpybmFEREEgPC0gJ1JOQS1zZXEvR1NFOTYwNThfZ2VuZV9leHByZXNzaW9uX1JlZHVjZWQudHN2JyAlPiUgCiAgcmVhZF90c3YKCmRlc2lnbiA8LSByZWFkX3RzdihkZXNpZ25QKSAlPiUgCiAgZmlsdGVyKCFpcy5uYShTYW1wbGVfdGl0bGUpKQpkYXRhRERBIDwtIHJlYWRfdHN2KGRkYVApICU+JSAKICBkcGx5cjo6c2VsZWN0KDE6MyxHZW5lLGRlc2lnbiRQb3NpdGlvbikKCmNvcnJlbGF0aW9uUm5hRERBTm9NQlIgPC0gbWVkaWFuRmlsdGVyaW5nUHJvdFJOQShwcm90RGF0YSA9IGRhdGFEREEscm5hRGF0YSA9IHJuYUREQSxkZXNpZ24gPSBkZXNpZ24pCndyaXRlX3Rzdih4ID0gY29ycmVsYXRpb25SbmFEREFOb01CUiRsb3dWYXIsZmlsZSA9ICdDb3JyZWxhdGlvbnMvY29ycmVsYXRpb25fUk5BX0REQV9Ob01CUl9Mb3dWYXIudHN2JykKYGBgCgoKIyMgQ29ycmVsYXRpb24gU3VtbWFyeQoKIyMjIEZ1bmN0aW9uCmBgYHtyfQpjb3JyU3VtbWFyeSA8LSBmdW5jdGlvbihkYXRhLCAuLi4pewogIGNvcnJlbGF0aW9uU3VtbWFyeSA8LSBkYXRhICU+JSAKICAgIHN1bW1hcmlzZSguLFBhaXJzPW5yb3coeCA9IC5bLDVdKSxQcm90ZWlucy5GRFIuU3BlYXJtYW4gPSBsZW5ndGgod2hpY2goUC5TcGVhcm1hbi5BZGo8MC4wNSkpLE1lZGlhbi5TcGVhcm1hbi5TY29yZT1tZWRpYW4oU3BlYXJtYW5fU2NvcmUsbmEucm0gPSBUKSxQcm90ZWlucy5GRFIuUGVhcnNvbiA9IGxlbmd0aCh3aGljaChQLlBlcmFzb24uQWRqPDAuMDUpKSxNZWRpYW4uUGVhcnNvbi5TY29yZT1tZWRpYW4oUGVhcnNvbl9TY29yZSxuYS5ybSA9IFQpKQp9CmBgYAoKIyMjIENvcnJlbGF0aW9uIFN1bW1hcnkgb2YgYWxsIGF2YWlsYWJsZSBmaWxlcwpgYGB7cn0KY29yckZpbGVzIDwtIGxpc3QuZmlsZXMocGF0dGVybiA9ICcqLnRzdicscGF0aCA9ICdDb3JyZWxhdGlvbnMnLGZ1bGwubmFtZXMgPSBUKQpjb3JyRGF0YSA8LSBtYXAoLnggPSBjb3JyRmlsZXMsLmYgPSByZWFkX3RzdikKbmFtZXMoY29yckRhdGEpIDwtIGNvcnJGaWxlcwpgYGAKYGBge3J9CmNvcnJOYW1lcyA8LSBuYW1lcyhjb3JyRGF0YSkgJT4lIAogIHN0cl9yZW1vdmVfYWxsKHBhdHRlcm4gPSAnQ29ycmVsYXRpb25zXFwvY29ycmVsYXRpb25cXF8nKSAlPiUgCiAgc3RyX3JlcGxhY2VfYWxsKHBhdHRlcm4gPSAnXFxfJyxyZXBsYWNlbWVudCA9ICcgJykgJT4lIAogIHN0cl9yZW1vdmVfYWxsKHBhdHRlcm4gPSAnXFwudHN2JykKICAKc3VtbWFyeVJlc3VsdHMgPC0gbWFwX2RmKC54ID0gY29yckRhdGEsLmYgPSBjb3JyU3VtbWFyeSkgJT4lIAogIG11dGF0ZSguLENvcnJlbGF0aW9uID0gY29yck5hbWVzLC5iZWZvcmU9J1BhaXJzJykKCndyaXRlX3Rzdih4ID0gc3VtbWFyeVJlc3VsdHMsZmlsZSA9ICdDb3JyZWxhdGlvbnMvQ29ycmVsYXRpb25fc3VtbWFyeS50c3YnKQpgYGAKCgojIEdlbmUgU2V0IEVucmljaG1lbnQgQW5hbHlzaXMgKEZpZ3VyZSAxKQoKIyMgRGlmZmVyZW50aWFsIEV4cHJlc3Npb24gQW5hbHlzaXMKCiAgVGhlIGRpZmZlcmVudGlhbCBleHByZXNzaW9uIGFuYWx5c2lzIHBlcmZvcm1lZCBoZXJlaW4gcGVydGFpbnMgdGhlIGRpZmZlcmVudCBpbnRyaW5zaWMgc3VidHlwZXMgKEFJTVMtUEFNNTApLCBpLmUuIGZlYXR1cmVzIGFyZSBkZXRlcm1pbmVkIGJhc2VkIG9uIDEtdnMtb3RoZXJzIGNvbXBhcmlzb25zLiBUaGUgc2FtcGxlcyB1c2VkIHdlcmUgZXhhY3RseSB0aGUgc2FtZSBhY3Jvc3MgZGlmZmVyZW50IGRhdGEgc291cmNlcy4KICAKIyMjIERJQS1OTiBQcm90ZW9taWNzCmBgYHtyfQojIEZvciBMdW1pbmFsIEEKZGF0YVAgPC0gJ0RJQS9EYXRhREUvRElBTk4vRElBX0RJQU5OX1Byb3RlaW5fUlJvbGx1cC9ESUFfRElBTk5fUlJvbGx1cF9HZW5lcy50c3YnCmRlc2lnblAgPC0gJ0RJQS9EZXNpZ25GaWxlc0RJQS9kZXNpZ25fYWltMl9jbGluaWNhbF9tYXRjaGVkX2luZGl2aWR1YWxzdWJ0eXBlcy50c3YnCm91dERpciA8LSAnREVfYW5hbHlzaXMvREVfZm9yX0dTRUEvJwpqREVOYW1lIDwtICJMdW1BIHZzIHJlc3QgUHJvdGVvbWljcyBBSU1TIFBBTTUwIgoKbm9ybWFseXplckRFKGpvYk5hbWUgPSBqREVOYW1lLCBkZXNpZ25QYXRoID0gZGVzaWduUCwgZGF0YVBhdGggPSBkYXRhUCwgbGVhc3RSZXBDb3VudCA9IDEsIHNhbXBsZUNvbD0iUG9zaXRpb24iLCBjb25kQ29sID0gIkFJTVNfUEFNNTAuTHVtQSIgLCBjb21wYXJpc29ucyA9IGMoJzEtMCcpLCBzaWdUaHJlcyA9IDAuMDUsIG91dHB1dERpciA9IG91dERpcixiYXRjaENvbCA9ICdncm91cCcpCmBgYApgYGB7cn0KIyBGb3IgTHVtaW5hbCBCCmRhdGFQIDwtICdESUEvRGF0YURFL0RJQU5OL0RJQV9ESUFOTl9Qcm90ZWluX1JSb2xsdXAvRElBX0RJQU5OX1JSb2xsdXBfR2VuZXMudHN2JwpkZXNpZ25QIDwtICdESUEvRGVzaWduRmlsZXNESUEvZGVzaWduX2FpbTJfY2xpbmljYWxfbWF0Y2hlZF9pbmRpdmlkdWFsc3VidHlwZXMudHN2JwpvdXREaXIgPC0gJ0RFX2FuYWx5c2lzL0RFX2Zvcl9HU0VBLycKakRFTmFtZSA8LSAiTHVtQiB2cyByZXN0IFByb3Rlb21pY3MgQUlNUyBQQU01MCIKCm5vcm1hbHl6ZXJERShqb2JOYW1lID0gakRFTmFtZSwgZGVzaWduUGF0aCA9IGRlc2lnblAsIGRhdGFQYXRoID0gZGF0YVAsIGxlYXN0UmVwQ291bnQgPSAyLCBzYW1wbGVDb2w9IlBvc2l0aW9uIiwgY29uZENvbCA9ICJBSU1TX1BBTTUwLkx1bUIiICwgY29tcGFyaXNvbnMgPSBjKCcxLTAnKSwgc2lnVGhyZXMgPSAwLjA1LCBvdXRwdXREaXIgPSBvdXREaXIsYmF0Y2hDb2wgPSAnZ3JvdXAnKQpgYGAKYGBge3J9CiMgRm9yIEJhc2FsCmRhdGFQIDwtICdESUEvRGF0YURFL0RJQU5OL0RJQV9ESUFOTl9Qcm90ZWluX1JSb2xsdXAvRElBX0RJQU5OX1JSb2xsdXBfR2VuZXMudHN2JwpkZXNpZ25QIDwtICdESUEvRGVzaWduRmlsZXNESUEvZGVzaWduX2FpbTJfY2xpbmljYWxfbWF0Y2hlZF9pbmRpdmlkdWFsc3VidHlwZXMudHN2JwpvdXREaXIgPC0gJ0RFX2FuYWx5c2lzL0RFX2Zvcl9HU0VBLycKakRFTmFtZSA8LSAiQmFzYWwgdnMgcmVzdCBQcm90ZW9taWNzIEFJTVMgUEFNNTAiCgpub3JtYWx5emVyREUoam9iTmFtZSA9IGpERU5hbWUsIGRlc2lnblBhdGggPSBkZXNpZ25QLCBkYXRhUGF0aCA9IGRhdGFQLCBsZWFzdFJlcENvdW50ID0gMiwgc2FtcGxlQ29sPSJQb3NpdGlvbiIsIGNvbmRDb2wgPSAiQUlNU19QQU01MC5CYXNhbCIgLCBjb21wYXJpc29ucyA9IGMoJzEtMCcpLCBzaWdUaHJlcyA9IDAuMDUsIG91dHB1dERpciA9IG91dERpcixiYXRjaENvbCA9ICdncm91cCcpCmBgYApgYGB7cn0KIyBGb3IgTm9ybWFsLWxpa2UKZGF0YVAgPC0gJ0RJQS9EYXRhREUvRElBTk4vRElBX0RJQU5OX1Byb3RlaW5fUlJvbGx1cC9ESUFfRElBTk5fUlJvbGx1cF9HZW5lcy50c3YnCmRlc2lnblAgPC0gJ0RJQS9EZXNpZ25GaWxlc0RJQS9kZXNpZ25fYWltMl9jbGluaWNhbF9tYXRjaGVkX2luZGl2aWR1YWxzdWJ0eXBlcy50c3YnCm91dERpciA8LSAnREVfYW5hbHlzaXMvREVfZm9yX0dTRUEvJwpqREVOYW1lIDwtICJOb3JtYWwgdnMgcmVzdCBQcm90ZW9taWNzIEFJTVMgUEFNNTAiCgpub3JtYWx5emVyREUoam9iTmFtZSA9IGpERU5hbWUsIGRlc2lnblBhdGggPSBkZXNpZ25QLCBkYXRhUGF0aCA9IGRhdGFQLCBsZWFzdFJlcENvdW50ID0gMSwgc2FtcGxlQ29sPSJQb3NpdGlvbiIsIGNvbmRDb2wgPSAiQUlNU19QQU01MC5Ob3JtYWwiICwgY29tcGFyaXNvbnMgPSBjKCcxLTAnKSwgc2lnVGhyZXMgPSAwLjA1LCBvdXRwdXREaXIgPSBvdXREaXIsYmF0Y2hDb2wgPSAnZ3JvdXAnKQpgYGAKYGBge3J9CiMgRm9yIEhFUjItZW5yaWNoZWQKZGF0YVAgPC0gJ0RJQS9EYXRhREUvRElBTk4vRElBX0RJQU5OX1Byb3RlaW5fUlJvbGx1cC9ESUFfRElBTk5fUlJvbGx1cF9HZW5lcy50c3YnCmRlc2lnblAgPC0gJ0RJQS9EZXNpZ25GaWxlc0RJQS9kZXNpZ25fYWltMl9jbGluaWNhbF9tYXRjaGVkX2luZGl2aWR1YWxzdWJ0eXBlcy50c3YnCm91dERpciA8LSAnREVfYW5hbHlzaXMvREVfZm9yX0dTRUEvJwpqREVOYW1lIDwtICJIZXIyIHZzIHJlc3QgUHJvdGVvbWljcyBBSU1TIFBBTTUwIgoKbm9ybWFseXplckRFKGpvYk5hbWUgPSBqREVOYW1lLCBkZXNpZ25QYXRoID0gZGVzaWduUCwgZGF0YVBhdGggPSBkYXRhUCwgbGVhc3RSZXBDb3VudCA9IDIsIHNhbXBsZUNvbD0iUG9zaXRpb24iLCBjb25kQ29sID0gIkFJTVNfUEFNNTAuSGVyMiIgLCBjb21wYXJpc29ucyA9IGMoJzEtMCcpLCBzaWdUaHJlcyA9IDAuMDUsIG91dHB1dERpciA9IG91dERpcixiYXRjaENvbCA9ICdncm91cCcpCmBgYAoKIyMjIFJOQS1zZXEKYGBge3J9CiMgRm9yIEx1bWluYWwgQQpkYXRhUCA8LSAnUk5BLXNlcS9HU0U5NjA1OF9nZW5lX2V4cHJlc3Npb25fUmVkdWNlZC50c3YnCmRlc2lnblAgPC0gJ0RJQS9EZXNpZ25GaWxlc0RJQS9kZXNpZ25fYWltMl9jbGluaWNhbF9tYXRjaGVkX2luZGl2aWR1YWxzdWJ0eXBlcy50c3YnCm91dERpciA8LSAnREVfYW5hbHlzaXMvREVfZm9yX0dTRUEvJwpqREVOYW1lIDwtICJMdW1BIHZzIHJlc3QgUk5Bc2VxIEFJTVMgUEFNNTAiCgpub3JtYWx5emVyREUoam9iTmFtZSA9IGpERU5hbWUsIGRlc2lnblBhdGggPSBkZXNpZ25QLCBkYXRhUGF0aCA9IGRhdGFQLCBsZWFzdFJlcENvdW50ID0gMSwgc2FtcGxlQ29sPSJTYW1wbGVfdGl0bGUiLCBjb25kQ29sID0gIkFJTVNfUEFNNTAuTHVtQSIgLCBjb21wYXJpc29ucyA9IGMoJzEtMCcpLCBzaWdUaHJlcyA9IDAuMDUsIG91dHB1dERpciA9IG91dERpcixiYXRjaENvbCA9ICdncm91cCcpCmBgYApgYGB7cn0KIyBGb3IgTHVtaW5hbCBCCmRhdGFQIDwtICdSTkEtc2VxL0dTRTk2MDU4X2dlbmVfZXhwcmVzc2lvbl9SZWR1Y2VkLnRzdicKZGVzaWduUCA8LSAnRElBL0Rlc2lnbkZpbGVzRElBL2Rlc2lnbl9haW0yX2NsaW5pY2FsX21hdGNoZWRfaW5kaXZpZHVhbHN1YnR5cGVzLnRzdicKb3V0RGlyIDwtICdERV9hbmFseXNpcy9ERV9mb3JfR1NFQS8nCmpERU5hbWUgPC0gIkx1bUIgdnMgcmVzdCBSTkFzZXEgQUlNUyBQQU01MCIKCm5vcm1hbHl6ZXJERShqb2JOYW1lID0gakRFTmFtZSwgZGVzaWduUGF0aCA9IGRlc2lnblAsIGRhdGFQYXRoID0gZGF0YVAsIGxlYXN0UmVwQ291bnQgPSAyLCBzYW1wbGVDb2w9IlNhbXBsZV90aXRsZSIsIGNvbmRDb2wgPSAiQUlNU19QQU01MC5MdW1CIiAsIGNvbXBhcmlzb25zID0gYygnMS0wJyksIHNpZ1RocmVzID0gMC4wNSwgb3V0cHV0RGlyID0gb3V0RGlyLGJhdGNoQ29sID0gJ2dyb3VwJykKYGBgCmBgYHtyfQojIEZvciBCYXNhbApkYXRhUCA8LSAnUk5BLXNlcS9HU0U5NjA1OF9nZW5lX2V4cHJlc3Npb25fUmVkdWNlZC50c3YnCmRlc2lnblAgPC0gJ0RJQS9EZXNpZ25GaWxlc0RJQS9kZXNpZ25fYWltMl9jbGluaWNhbF9tYXRjaGVkX2luZGl2aWR1YWxzdWJ0eXBlcy50c3YnCm91dERpciA8LSAnREVfYW5hbHlzaXMvREVfZm9yX0dTRUEvJwpqREVOYW1lIDwtICJCYXNhbCB2cyByZXN0IFJOQXNlcSBBSU1TIFBBTTUwIgoKbm9ybWFseXplckRFKGpvYk5hbWUgPSBqREVOYW1lLCBkZXNpZ25QYXRoID0gZGVzaWduUCwgZGF0YVBhdGggPSBkYXRhUCwgbGVhc3RSZXBDb3VudCA9IDIsIHNhbXBsZUNvbD0iU2FtcGxlX3RpdGxlIiwgY29uZENvbCA9ICJBSU1TX1BBTTUwLkJhc2FsIiAsIGNvbXBhcmlzb25zID0gYygnMS0wJyksIHNpZ1RocmVzID0gMC4wNSwgb3V0cHV0RGlyID0gb3V0RGlyLGJhdGNoQ29sID0gJ2dyb3VwJykKYGBgCmBgYHtyfQojIEZvciBOb3JtYWwtbGlrZQpkYXRhUCA8LSAnUk5BLXNlcS9HU0U5NjA1OF9nZW5lX2V4cHJlc3Npb25fUmVkdWNlZC50c3YnCmRlc2lnblAgPC0gJ0RJQS9EZXNpZ25GaWxlc0RJQS9kZXNpZ25fYWltMl9jbGluaWNhbF9tYXRjaGVkX2luZGl2aWR1YWxzdWJ0eXBlcy50c3YnCm91dERpciA8LSAnREVfYW5hbHlzaXMvREVfZm9yX0dTRUEvJwpqREVOYW1lIDwtICJOb3JtYWwgdnMgcmVzdCBSTkFzZXEgQUlNUyBQQU01MCIKCm5vcm1hbHl6ZXJERShqb2JOYW1lID0gakRFTmFtZSwgZGVzaWduUGF0aCA9IGRlc2lnblAsIGRhdGFQYXRoID0gZGF0YVAsIGxlYXN0UmVwQ291bnQgPSAxLCBzYW1wbGVDb2w9IlNhbXBsZV90aXRsZSIsIGNvbmRDb2wgPSAiQUlNU19QQU01MC5Ob3JtYWwiICwgY29tcGFyaXNvbnMgPSBjKCcxLTAnKSwgc2lnVGhyZXMgPSAwLjA1LCBvdXRwdXREaXIgPSBvdXREaXIsYmF0Y2hDb2wgPSAnZ3JvdXAnKQpgYGAKYGBge3J9CiMgRm9yIEhFUjItZW5yaWNoZWQKZGF0YVAgPC0gJ1JOQS1zZXEvR1NFOTYwNThfZ2VuZV9leHByZXNzaW9uX1JlZHVjZWQudHN2JwpkZXNpZ25QIDwtICdESUEvRGVzaWduRmlsZXNESUEvZGVzaWduX2FpbTJfY2xpbmljYWxfbWF0Y2hlZF9pbmRpdmlkdWFsc3VidHlwZXMudHN2JwpvdXREaXIgPC0gJ0RFX2FuYWx5c2lzL0RFX2Zvcl9HU0VBLycKakRFTmFtZSA8LSAiSGVyMiB2cyByZXN0IFJOQXNlcSBBSU1TIFBBTTUwIgoKbm9ybWFseXplckRFKGpvYk5hbWUgPSBqREVOYW1lLCBkZXNpZ25QYXRoID0gZGVzaWduUCwgZGF0YVBhdGggPSBkYXRhUCwgbGVhc3RSZXBDb3VudCA9IDIsIHNhbXBsZUNvbD0iU2FtcGxlX3RpdGxlIiwgY29uZENvbCA9ICJBSU1TX1BBTTUwLkhlcjIiICwgY29tcGFyaXNvbnMgPSBjKCcxLTAnKSwgc2lnVGhyZXMgPSAwLjA1LCBvdXRwdXREaXIgPSBvdXREaXIsYmF0Y2hDb2wgPSAnZ3JvdXAnKQpgYGAKCiMjIEZ1bmN0aW9uCmBgYHtyfQpnc2VhTXNpZ0RCIDwtIGZ1bmN0aW9uKGRhdGEsZ2VuZWNvbD0nR2VuZXMnLHF2YWx1ZWN1dG9mZixwYWRqdXN0bWV0aG9kPSdmZHInLHNwZWNpZXM9J0hvbW8gc2FwaWVucycsY2F0ZWdvcnk9J0gnLEZDY29sbmFtZT0nMS0wX2xvZzJGb2xkQ2hhbmdlJywuLi4pewogIGRhdGEgPC0gcmVhZF90c3YoZGF0YSkKICBGQ2NvbCA8LSBkYXRhICU+JSBkcGx5cjo6cHVsbChGQ2NvbG5hbWUpCiAgZGF0YSA8LSBkYXRhICU+JSBmaWx0ZXIoIWlzLm5hKEZDY29sKSkKICBnZW5lbGlzdCA8LSBkYXRhICU+JSBwdWxsKEZDY29sbmFtZSkKICBuYW1lcyhnZW5lbGlzdCkgPC0gZGF0YSAlPiUgZHBseXI6OnB1bGwoZ2VuZWNvbCkKICBnZW5lbGlzdCA8LSBuYS5vbWl0KGdlbmVsaXN0KQogIGdlbmVsaXN0IDwtIHNvcnQoeCA9IGdlbmVsaXN0LGRlY3JlYXNpbmcgPSBUUlVFKQogIAogIHRlcm0yZ2VuZSA8LSBtc2lnZGJyKHNwZWNpZXMgPSBzcGVjaWVzLGNhdGVnb3J5ID0gY2F0ZWdvcnkpICU+JSBkcGx5cjo6c2VsZWN0KGdzX25hbWUsaHVtYW5fZ2VuZV9zeW1ib2wpCiAgCiAgZ3NlUmVzdWx0cyA8LSBjbHVzdGVyUHJvZmlsZXI6OkdTRUEoZ2VuZUxpc3QgPSBnZW5lbGlzdCwKICAgICAgICAgICAgICAgICAgICAgICAgICAgICAgIHBBZGp1c3RNZXRob2QgPSAnZmRyJywKICAgICAgICAgICAgICAgICAgICAgICAgICAgICAgIGVwcyA9IDAsCiAgICAgICAgICAgICAgICAgICAgICAgICAgICAgICBwdmFsdWVDdXRvZmYgPSBxdmFsdWVjdXRvZmYsCiAgICAgICAgICAgICAgICAgICAgICAgICAgICAgICBURVJNMkdFTkUgPSB0ZXJtMmdlbmUsCiAgICAgICAgICAgICAgICAgICAgICAgICAgICAgICBieSA9ICdmZ3NlYScpCiAgCiAgcmV0dXJuKGdzZVJlc3VsdHMpCn0KYGBgCgojIyBHU0VBIERJQS1OTgpgYGB7cn0KI0x1bWluYWwgQQpkYXRhIDwtICdERV9hbmFseXNpcy9ERV9mb3JfR1NFQS9MdW1BIHZzIHJlc3QgUHJvdGVvbWljcyBBSU1TIFBBTTUwL0x1bUEgdnMgcmVzdCBQcm90ZW9taWNzIEFJTVMgUEFNNTBfc3RhdHMudHN2Jwpnc2VhTXNpZ0x1bUEgPC0gZ3NlYU1zaWdEQihkYXRhID0gZGF0YSxxdmFsdWVjdXRvZmYgPSAwLjI1LHBhZGp1c3RtZXRob2QgPSAnZmRyJyxzcGVjaWVzID0gJ0hvbW8gc2FwaWVucycsY2F0ZWdvcnkgPSAnSCcpICU+JSBkcGx5cjo6bXV0YXRlKFBBTTUwPSdMdW1BJykKCiNMdW1pbmFsIEIKZGF0YSA8LSAnREVfYW5hbHlzaXMvREVfZm9yX0dTRUEvTHVtQiB2cyByZXN0IFByb3Rlb21pY3MgQUlNUyBQQU01MC9MdW1CIHZzIHJlc3QgUHJvdGVvbWljcyBBSU1TIFBBTTUwX3N0YXRzLnRzdicKZ3NlYU1zaWdMdW1CIDwtIGdzZWFNc2lnREIoZGF0YSA9IGRhdGEscXZhbHVlY3V0b2ZmID0gMC4yNSxwYWRqdXN0bWV0aG9kID0gJ2Zkcicsc3BlY2llcyA9ICdIb21vIHNhcGllbnMnLGNhdGVnb3J5ID0gJ0gnKSAlPiUgZHBseXI6Om11dGF0ZShQQU01MD0nTHVtQicpCgojQmFzYWwKZGF0YSA8LSAnREVfYW5hbHlzaXMvREVfZm9yX0dTRUEvQmFzYWwgdnMgcmVzdCBQcm90ZW9taWNzIEFJTVMgUEFNNTAvQmFzYWwgdnMgcmVzdCBQcm90ZW9taWNzIEFJTVMgUEFNNTBfc3RhdHMudHN2Jwpnc2VhTXNpZ0Jhc2FsIDwtIGdzZWFNc2lnREIoZGF0YSA9IGRhdGEscXZhbHVlY3V0b2ZmID0gMC4yNSxwYWRqdXN0bWV0aG9kID0gJ2Zkcicsc3BlY2llcyA9ICdIb21vIHNhcGllbnMnLGNhdGVnb3J5ID0gJ0gnKSAlPiUgZHBseXI6Om11dGF0ZShQQU01MD0nQmFzYWwnKQoKI0hFUjItZW5yaWNoZWQKZGF0YSA8LSAnREVfYW5hbHlzaXMvREVfZm9yX0dTRUEvSGVyMiB2cyByZXN0IFByb3Rlb21pY3MgQUlNUyBQQU01MC9IZXIyIHZzIHJlc3QgUHJvdGVvbWljcyBBSU1TIFBBTTUwX3N0YXRzLnRzdicKZ3NlYU1zaWdIZXIyIDwtIGdzZWFNc2lnREIoZGF0YSA9IGRhdGEscXZhbHVlY3V0b2ZmID0gMC4yNSxwYWRqdXN0bWV0aG9kID0gJ2Zkcicsc3BlY2llcyA9ICdIb21vIHNhcGllbnMnLGNhdGVnb3J5ID0gJ0gnKSAlPiUgZHBseXI6Om11dGF0ZShQQU01MD0nSGVyMicpCgojTm9ybWFsLWxpa2UKZGF0YSA8LSAnREVfYW5hbHlzaXMvREVfZm9yX0dTRUEvTm9ybWFsIHZzIHJlc3QgUHJvdGVvbWljcyBBSU1TIFBBTTUwL05vcm1hbCB2cyByZXN0IFByb3Rlb21pY3MgQUlNUyBQQU01MF9zdGF0cy50c3YnCmdzZWFNc2lnTm9ybWFsIDwtIGdzZWFNc2lnREIoZGF0YSA9IGRhdGEscXZhbHVlY3V0b2ZmID0gMC4yNSxwYWRqdXN0bWV0aG9kID0gJ2Zkcicsc3BlY2llcyA9ICdIb21vIHNhcGllbnMnLGNhdGVnb3J5ID0gJ0gnKSAlPiUgZHBseXI6Om11dGF0ZShQQU01MD0nTm9ybWFsJykKYGBgCgojIyBHU0VBIFJOQS1zZXEKYGBge3J9CiNMdW1pbmFsIEEgCmRhdGEgPC0gJ0RFX2FuYWx5c2lzL0RFX2Zvcl9HU0VBL0x1bUEgdnMgcmVzdCBSTkFzZXEgQUlNUyBQQU01MC9MdW1BIHZzIHJlc3QgUk5Bc2VxIEFJTVMgUEFNNTBfc3RhdHMudHN2Jwpnc2VhTXNpZ0x1bUFSTkEgPC0gZ3NlYU1zaWdEQihkYXRhID0gZGF0YSxnZW5lY29sID0gJ0dlbmUnLHF2YWx1ZWN1dG9mZiA9IDAuMjUscGFkanVzdG1ldGhvZCA9ICdmZHInLHNwZWNpZXMgPSAnSG9tbyBzYXBpZW5zJyxjYXRlZ29yeSA9ICdIJykgJT4lIGRwbHlyOjptdXRhdGUoUEFNNTA9J0x1bUEnKQoKI0x1bWluYWwgQgpkYXRhIDwtICdERV9hbmFseXNpcy9ERV9mb3JfR1NFQS9MdW1CIHZzIHJlc3QgUk5Bc2VxIEFJTVMgUEFNNTAvTHVtQiB2cyByZXN0IFJOQXNlcSBBSU1TIFBBTTUwX3N0YXRzLnRzdicKZ3NlYU1zaWdMdW1CUk5BIDwtIGdzZWFNc2lnREIoZGF0YSA9IGRhdGEsZ2VuZWNvbCA9ICdHZW5lJyxxdmFsdWVjdXRvZmYgPSAwLjI1LHBhZGp1c3RtZXRob2QgPSAnZmRyJyxzcGVjaWVzID0gJ0hvbW8gc2FwaWVucycsY2F0ZWdvcnkgPSAnSCcpICU+JSBkcGx5cjo6bXV0YXRlKFBBTTUwPSdMdW1CJykKCiNCYXNhbCAKZGF0YSA8LSAnREVfYW5hbHlzaXMvREVfZm9yX0dTRUEvQmFzYWwgdnMgcmVzdCBSTkFzZXEgQUlNUyBQQU01MC9CYXNhbCB2cyByZXN0IFJOQXNlcSBBSU1TIFBBTTUwX3N0YXRzLnRzdicKZ3NlYU1zaWdCYXNhbFJOQSA8LSBnc2VhTXNpZ0RCKGRhdGEgPSBkYXRhLGdlbmVjb2wgPSAnR2VuZScscXZhbHVlY3V0b2ZmID0gMC4yNSxwYWRqdXN0bWV0aG9kID0gJ2Zkcicsc3BlY2llcyA9ICdIb21vIHNhcGllbnMnLGNhdGVnb3J5ID0gJ0gnKSAlPiUgZHBseXI6Om11dGF0ZShQQU01MD0nQmFzYWwnKQoKI0hFUjItZW5yaWNoZWQKZGF0YSA8LSAnREVfYW5hbHlzaXMvREVfZm9yX0dTRUEvSGVyMiB2cyByZXN0IFJOQXNlcSBBSU1TIFBBTTUwL0hlcjIgdnMgcmVzdCBSTkFzZXEgQUlNUyBQQU01MF9zdGF0cy50c3YnCmdzZWFNc2lnSGVyMlJOQSA8LSBnc2VhTXNpZ0RCKGRhdGEgPSBkYXRhLGdlbmVjb2wgPSAnR2VuZScscXZhbHVlY3V0b2ZmID0gMC4yNSxwYWRqdXN0bWV0aG9kID0gJ2Zkcicsc3BlY2llcyA9ICdIb21vIHNhcGllbnMnLGNhdGVnb3J5ID0gJ0gnKSAlPiUgZHBseXI6Om11dGF0ZShQQU01MD0nSGVyMicpCgojTm9ybWFsLWxpa2UKZGF0YSA8LSAnREVfYW5hbHlzaXMvREVfZm9yX0dTRUEvTm9ybWFsIHZzIHJlc3QgUk5Bc2VxIEFJTVMgUEFNNTAvTm9ybWFsIHZzIHJlc3QgUk5Bc2VxIEFJTVMgUEFNNTBfc3RhdHMudHN2Jwpnc2VhTXNpZ05vcm1hbFJOQSA8LSBnc2VhTXNpZ0RCKGRhdGEgPSBkYXRhLGdlbmVjb2wgPSAnR2VuZScscXZhbHVlY3V0b2ZmID0gMC4yNSxwYWRqdXN0bWV0aG9kID0gJ2Zkcicsc3BlY2llcyA9ICdIb21vIHNhcGllbnMnLGNhdGVnb3J5ID0gJ0gnKSAlPiUgZHBseXI6Om11dGF0ZShQQU01MD0nTm9ybWFsJykKYGBgCgojIyBQbG90dGluZwpgYGB7cn0KYWxsUmVzdWx0c0dTRUFNc2lnIDwtIGxpc3QoQmFzYWw9Z3NlYU1zaWdCYXNhbCxIZXIyPWdzZWFNc2lnSGVyMixMdW1BPWdzZWFNc2lnTHVtQSxMdW1CPWdzZWFNc2lnTHVtQixOb3JtYWw9Z3NlYU1zaWdOb3JtYWwpCmFsbFJlc3VsdHNHU0VBTXNpZyA8LSBtYXAoYWxsUmVzdWx0c0dTRUFNc2lnLH5wbHVjaygueCwncmVzdWx0JykpICU+JSAKICBtYXAoLixhc190aWJibGUpICU+JSAKICBiaW5kX3Jvd3MoLikKCmFsbFJlc3VsdHNHU0VBTXNpZ1JOQSA8LSBsaXN0KEJhc2FsPWdzZWFNc2lnQmFzYWxSTkEsSGVyMj1nc2VhTXNpZ0hlcjJSTkEsTHVtQT1nc2VhTXNpZ0x1bUFSTkEsTHVtQj1nc2VhTXNpZ0x1bUJSTkEsTm9ybWFsPWdzZWFNc2lnTm9ybWFsUk5BKQphbGxSZXN1bHRzR1NFQU1zaWdSTkEgPC0gbWFwKGFsbFJlc3VsdHNHU0VBTXNpZ1JOQSx+cGx1Y2soLngsJ3Jlc3VsdCcpKSAlPiUgCiAgbWFwKC4sYXNfdGliYmxlKSAlPiUgCiAgYmluZF9yb3dzKC4pCgpyZXN1bHRzQWxsIDwtIGZ1bGxfam9pbihhbGxSZXN1bHRzR1NFQU1zaWcsYWxsUmVzdWx0c0dTRUFNc2lnUk5BLGJ5PWMoJ0lEJywnUEFNNTAnKSxzdWZmaXg9YygnLkRJQScsJy5STkEnKSkKcmVzdWx0c0FsbCRJRCA8LSByZXN1bHRzQWxsJElEICU+JSAKICBzdHJfcmVtb3ZlKHN0cmluZyA9IC4scGF0dGVybiA9ICdIQUxMTUFSS1xcXycpICU+JSAKICBzdHJfcmVwbGFjZV9hbGwoLiwnXFxfJywnICcpCgpwbG90U2VwYXJhdGVTdWJ0eXBlcyA8LSBnZ3Bsb3QoZGF0YSA9IHJlc3VsdHNBbGwpICsgCiAgZ2VvbV9wb2ludChtYXBwaW5nID0gYWVzKHggPSBORVMuUk5BLHkgPSBmY3RfcmVvcmRlciguZiA9IElELC54ID0gMS0ocXZhbHVlcy5STkEpKSxzaXplID0gcXZhbHVlcy5STkEsZmlsbCA9ICdSTkEnKSxzaGFwZSA9IDIxLGFscGhhPTAuODUpCgpwbG90U2VwYXJhdGVTdWJ0eXBlcyA8LSBwbG90U2VwYXJhdGVTdWJ0eXBlcyArIAogIGdlb21fcG9pbnQobWFwcGluZyA9IGFlcyh4ID0gTkVTLkRJQSx5ID0gSUQsc2l6ZSA9IHF2YWx1ZXMuRElBLGZpbGwgPSAnRElBJyksc2hhcGUgPSAyMSxhbHBoYT0wLjg1KSArIAogIHRoZW1lX2J3KCkgKyAKICBsYWJzKCB4ID0gJ05vcm1hbGl6ZWQgRW5yaWNobWVudCBTY29yZScseT0nJyx0aXRsZSA9ICdHU0VBIC0gTVNpZ0RCIEhhbGxtYXJrcycpICsgCiAgc2NhbGVfcmFkaXVzKCJxLXZhbHVlcyIsIHRyYW5zPSJsb2cxMCIsIHJhbmdlPWMoNywgMykpKwogIHNjYWxlX2ZpbGxfZGlzY3JldGUoJ0V4cGVyaW1lbnQnLHR5cGUgPSBjKCcjY2EwMDIwJywnIzA1NzFiMCcpKSsKICBmYWNldF93cmFwKHZhcnMoUEFNNTApLG5jb2wgPSA1KQoKZ2dzYXZlKGZpbGVuYW1lID0gJ0ZpZ3VyZTEucG5nJyxwbG90ID0gcGxvdFNlcGFyYXRlU3VidHlwZXMsZGV2aWNlID0gJ3BuZycscGF0aCA9ICdHU0VBLycsdW5pdHMgPSAnaW4nLHdpZHRoID0gOSxoZWlnaHQgPSA3LGRwaSA9IDYwMCkKYGBgCgoKIyBEZWNpc2lvbiBUcmVlIChGaWd1cmVzIDIgYW5kIDMpCgojIyBEaWZmZXJlbnRpYWwgRXhwcmVzc2lvbiBBbmFseXNpcwoKICBJbiBvcmRlciB0byBnZW5lcmF0ZSB0aGUgZGVjaXNpb24gdHJlZSBtb2RlbCwgYSBtaXh0dXJlIG9mIGZlYXR1cmVzIGRpZmZlcmVudGlhbGx5IGV4cHJlc3NlZCBpbiBkaWZmZXJlbnQgc3VidHlwZXMgYXMgd2VsbCBhcyBpbiBjbGluaWNvcGF0aG9sb2dpY2FsIHBhcmFtZXRlcnMgd2FzIHVzZWQuIE9ubHkgcHJvdGVvbWljcyBkYXRhIHdlcmUgdXNlZCB0byBnZW5lcmF0ZSB0aGUgbW9kZWwuCmBgYHtyfQojIEVzdHJvZ2VuIHJlY2VwdG9yIHBvc2l0aXZlIHZzIEVzdHJvZ2VuIHJlY2VwdG9yIG5lZ2F0aXZlCmRhdGFQIDwtICdESUEvRGF0YURFL0RJQU5OL0RJQV9ESUFOTl9Qcm90ZWluX1JSb2xsdXAvRElBX0RJQU5OX1JSb2xsdXBfR2VuZXMudHN2JwpkZXNpZ25QIDwtICdESUEvRGVzaWduRmlsZXNESUEvZGVzaWduX2FpbTJfY2xpbmljYWxfRGVjaXNpb25UcmVlLnRzdicKb3V0RGlyIDwtICdERV9hbmFseXNpcy9ERV9mb3JfZGVjaXNpb250cmVlLycKakRFTmFtZSA8LSAiRVJwIHZzIEVSbiIKCm5vcm1hbHl6ZXJERShqb2JOYW1lID0gakRFTmFtZSxkZXNpZ25QYXRoID0gZGVzaWduUCxkYXRhUGF0aCA9IGRhdGFQLG91dHB1dERpciA9IG91dERpcixzYW1wbGVDb2wgPSAnUG9zaXRpb24nLGNvbmRDb2wgPSAnRVJfc3RhdHVzJyxjb21wYXJpc29ucyA9IGMoJ1Bvcy1OZWcnKSxsZWFzdFJlcENvdW50ID0gMykKYGBgCmBgYHtyfQojIEx5bXBobm9kZSBwb3NpdGl2ZSB2cyBMeW1waG5vZGUgbmVnYXRpdmUKZGF0YVAgPC0gJ0RJQS9EYXRhREUvRElBTk4vRElBX0RJQU5OX1Byb3RlaW5fUlJvbGx1cC9ESUFfRElBTk5fUlJvbGx1cF9HZW5lcy50c3YnCmRlc2lnblAgPC0gJ0RJQS9EZXNpZ25GaWxlc0RJQS9kZXNpZ25fYWltMl9jbGluaWNhbF9EZWNpc2lvblRyZWUudHN2JwpvdXREaXIgPC0gJ0RFX2FuYWx5c2lzL0RFX2Zvcl9kZWNpc2lvbnRyZWUvJwpqREVOYW1lIDwtICJOb2RlUG9zaXRpdmUgdnMgTm9kZU5lZ2F0aXZlIgoKbm9ybWFseXplckRFKGpvYk5hbWUgPSBqREVOYW1lLGRlc2lnblBhdGggPSBkZXNpZ25QLGRhdGFQYXRoID0gZGF0YVAsb3V0cHV0RGlyID0gb3V0RGlyLHNhbXBsZUNvbCA9ICdQb3NpdGlvbicsY29uZENvbCA9ICdMTl9zdGF0dXMnLGNvbXBhcmlzb25zID0gYygnTm9kZVBvc2l0aXZlLU5vZGVOZWdhdGl2ZScpLGxlYXN0UmVwQ291bnQgPSAzKQpgYGAKYGBge3J9CiMgSEVSMiByZWNlcHRvciBwb3NpdGl2ZSB2cyBIRVIyIHJlY2VwdG9yIG5lZ2F0aXZlCmRhdGFQIDwtICdESUEvRGF0YURFL0RJQU5OL0RJQV9ESUFOTl9Qcm90ZWluX1JSb2xsdXAvRElBX0RJQU5OX1JSb2xsdXBfR2VuZXMudHN2JwpkZXNpZ25QIDwtICdESUEvRGVzaWduRmlsZXNESUEvZGVzaWduX2FpbTJfY2xpbmljYWxfRGVjaXNpb25UcmVlLnRzdicKb3V0RGlyIDwtICdERV9hbmFseXNpcy9ERV9mb3JfZGVjaXNpb250cmVlLycKakRFTmFtZSA8LSAiSGVyMnAgdnMgSGVyMm4iCgpub3JtYWx5emVyREUoam9iTmFtZSA9IGpERU5hbWUsZGVzaWduUGF0aCA9IGRlc2lnblAsZGF0YVBhdGggPSBkYXRhUCxvdXRwdXREaXIgPSBvdXREaXIsc2FtcGxlQ29sID0gJ1Bvc2l0aW9uJyxjb25kQ29sID0gJ0hFUjJfc3RhdHVzJyxjb21wYXJpc29ucyA9IGMoJ1Bvcy1OZWcnKSxsZWFzdFJlcENvdW50ID0gMykKYGBgCmBgYHtyfQojIFR1bW9yIEdyYWRlIDMgdnMgVHVtb3IgR3JhZGUgMQpkYXRhUCA8LSAnRElBL0RhdGFERS9ESUFOTi9ESUFfRElBTk5fUHJvdGVpbl9SUm9sbHVwL0RJQV9ESUFOTl9SUm9sbHVwX0dlbmVzLnRzdicKZGVzaWduUCA8LSAnRElBL0Rlc2lnbkZpbGVzRElBL2Rlc2lnbl9haW0yX2NsaW5pY2FsX0RlY2lzaW9uVHJlZS50c3YnCm91dERpciA8LSAnREVfYW5hbHlzaXMvREVfZm9yX2RlY2lzaW9udHJlZS8nCmpERU5hbWUgPC0gIkdyYWRlMyB2cyBHcmFkZTEiCgpub3JtYWx5emVyREUoam9iTmFtZSA9IGpERU5hbWUsZGVzaWduUGF0aCA9IGRlc2lnblAsZGF0YVBhdGggPSBkYXRhUCxvdXRwdXREaXIgPSBvdXREaXIsc2FtcGxlQ29sID0gJ1Bvc2l0aW9uJyxjb25kQ29sID0gJ0dyYWRlJyxjb21wYXJpc29ucyA9IGMoJzEtMycpLGxlYXN0UmVwQ291bnQgPSAzKQpgYGAKYGBge3J9CiMgQmFzYWwgdnMgTHVtaW5hbCBBCmRhdGFQIDwtICdESUEvRGF0YURFL0RJQU5OL0RJQV9ESUFOTl9Qcm90ZWluX1JSb2xsdXAvRElBX0RJQU5OX1JSb2xsdXBfR2VuZXMudHN2JwpkZXNpZ25QIDwtICdESUEvRGVzaWduRmlsZXNESUEvZGVzaWduX2FpbTJfY2xpbmljYWxfRGVjaXNpb25UcmVlLnRzdicKb3V0RGlyIDwtICdERV9hbmFseXNpcy9ERV9mb3JfZGVjaXNpb250cmVlLycKakRFTmFtZSA8LSAiQmFzYWwgdnMgTHVtQSIKCm5vcm1hbHl6ZXJERShqb2JOYW1lID0gakRFTmFtZSxkZXNpZ25QYXRoID0gZGVzaWduUCxkYXRhUGF0aCA9IGRhdGFQLG91dHB1dERpciA9IG91dERpcixzYW1wbGVDb2wgPSAnUG9zaXRpb24nLGNvbmRDb2wgPSAnQUlNU19QQU01MCcsY29tcGFyaXNvbnMgPSBjKCdCYXNhbC1MdW1BJyksbGVhc3RSZXBDb3VudCA9IDMpCmBgYApgYGB7cn0KIyBMdW1pbmFsIEEgdnMgTHVtaW5hbCBCCmRhdGFQIDwtICdESUEvRGF0YURFL0RJQU5OL0RJQV9ESUFOTl9Qcm90ZWluX1JSb2xsdXAvRElBX0RJQU5OX1JSb2xsdXBfR2VuZXMudHN2JwpkZXNpZ25QIDwtICdESUEvRGVzaWduRmlsZXNESUEvZGVzaWduX2FpbTJfY2xpbmljYWxfRGVjaXNpb25UcmVlLnRzdicKb3V0RGlyIDwtICdERV9hbmFseXNpcy9ERV9mb3JfZGVjaXNpb250cmVlLycKakRFTmFtZSA8LSAiTHVtQSB2cyBMdW1CIgoKbm9ybWFseXplckRFKGpvYk5hbWUgPSBqREVOYW1lLGRlc2lnblBhdGggPSBkZXNpZ25QLGRhdGFQYXRoID0gZGF0YVAsb3V0cHV0RGlyID0gb3V0RGlyLHNhbXBsZUNvbCA9ICdQb3NpdGlvbicsY29uZENvbCA9ICdBSU1TX1BBTTUwJyxjb21wYXJpc29ucyA9IGMoJ0x1bUEtTHVtQicpLGxlYXN0UmVwQ291bnQgPSAzKQpgYGAKYGBge3J9CiMgTHVtaW5hbCBCIHZzIEhFUjItZW5yaWNoZWQKZGF0YVAgPC0gJ0RJQS9EYXRhREUvRElBTk4vRElBX0RJQU5OX1Byb3RlaW5fUlJvbGx1cC9ESUFfRElBTk5fUlJvbGx1cF9HZW5lcy50c3YnCmRlc2lnblAgPC0gJ0RJQS9EZXNpZ25GaWxlc0RJQS9kZXNpZ25fYWltMl9jbGluaWNhbF9EZWNpc2lvblRyZWUudHN2JwpvdXREaXIgPC0gJ0RFX2FuYWx5c2lzL0RFX2Zvcl9kZWNpc2lvbnRyZWUvJwpqREVOYW1lIDwtICJMdW1CIHZzIEhlcjIiCgpub3JtYWx5emVyREUoam9iTmFtZSA9IGpERU5hbWUsZGVzaWduUGF0aCA9IGRlc2lnblAsZGF0YVBhdGggPSBkYXRhUCxvdXRwdXREaXIgPSBvdXREaXIsc2FtcGxlQ29sID0gJ1Bvc2l0aW9uJyxjb25kQ29sID0gJ0FJTVNfUEFNNTAnLGNvbXBhcmlzb25zID0gYygnTHVtQi1IZXIyJyksbGVhc3RSZXBDb3VudCA9IDMpCmBgYApgYGB7cn0KIyBCYXNhbCB2cyBIRVIyLWVucmljaGVkCmRhdGFQIDwtICdESUEvRGF0YURFL0RJQU5OL0RJQV9ESUFOTl9Qcm90ZWluX1JSb2xsdXAvRElBX0RJQU5OX1JSb2xsdXBfR2VuZXMudHN2JwpkZXNpZ25QIDwtICdESUEvRGVzaWduRmlsZXNESUEvZGVzaWduX2FpbTJfY2xpbmljYWxfRGVjaXNpb25UcmVlLnRzdicKb3V0RGlyIDwtICdERV9hbmFseXNpcy9ERV9mb3JfZGVjaXNpb250cmVlLycKakRFTmFtZSA8LSAiQmFzYWwgdnMgSGVyMiIKCm5vcm1hbHl6ZXJERShqb2JOYW1lID0gakRFTmFtZSxkZXNpZ25QYXRoID0gZGVzaWduUCxkYXRhUGF0aCA9IGRhdGFQLG91dHB1dERpciA9IG91dERpcixzYW1wbGVDb2wgPSAnUG9zaXRpb24nLGNvbmRDb2wgPSAnQUlNU19QQU01MCcsY29tcGFyaXNvbnMgPSBjKCdCYXNhbC1IZXIyJyksbGVhc3RSZXBDb3VudCA9IDMpCmBgYApgYGB7cn0KIyBIRVIyLWVucmljaGVkIHZzIEx1bWluYWwgQQpkYXRhUCA8LSAnRElBL0RhdGFERS9ESUFOTi9ESUFfRElBTk5fUHJvdGVpbl9SUm9sbHVwL0RJQV9ESUFOTl9SUm9sbHVwX0dlbmVzLnRzdicKZGVzaWduUCA8LSAnRElBL0Rlc2lnbkZpbGVzRElBL2Rlc2lnbl9haW0yX2NsaW5pY2FsX0RlY2lzaW9uVHJlZS50c3YnCm91dERpciA8LSAnREVfYW5hbHlzaXMvREVfZm9yX2RlY2lzaW9udHJlZS8nCmpERU5hbWUgPC0gIkhlcjIgdnMgTHVtQSIKCm5vcm1hbHl6ZXJERShqb2JOYW1lID0gakRFTmFtZSxkZXNpZ25QYXRoID0gZGVzaWduUCxkYXRhUGF0aCA9IGRhdGFQLG91dHB1dERpciA9IG91dERpcixzYW1wbGVDb2wgPSAnUG9zaXRpb24nLGNvbmRDb2wgPSAnQUlNU19QQU01MCcsY29tcGFyaXNvbnMgPSBjKCdIZXIyLUx1bUEnKSxsZWFzdFJlcENvdW50ID0gMykKYGBgCgojIyBEYXRhIEltcG9ydCBhbmQgcHJlcGFyaW5nIHRyYWluaW5nIGFuZCB0ZXN0IHNldHMKCiAgVGhlIGlucHV0IGRhdGEgZm9yIHRoZSBkZWNpc2lvbiB0cmVlIG1vZGVsIGlzIGEgbGlzdCBvZiBkaWZmZXJlbnRpYWxseSBleHByZXNzZWQgcHJvdGVpbnMgYWNyb3NzIGRpZmZlcmVudCBjb25kaXRpb25zIGFmdGVyIGZpbHRlcmluZyBiYXNlZCBvbiBhZGp1c3RlZCBwLXZhbHVlIG9mIDAuMDEgYW5kIGFic29sdXRlIGxvZzJGb2xkQ2hhbmdlIG9mIDIgYW5kIGNvbGxhcHNpbmcgdGhlIGRhdGEgaW50byB1bmlxdWUgUHJvdGVpbiBHcm91cCBJRHMuIFRoZSBESUEtTk4gZXhwcmVzc2lvbiBkYXRhIGlzIHRoZW4gZmlsdGVyZWQgYmFzZWQgb24gdGhlc2UgSURzIGFuZCB0aGUgbm9ybWFsaXplZCBhYnVuZGFuY2UgdmFsdWVzIGFyZSB1c2VkIGZvciB0aGUgbW9kZWwuCmBgYHtiYXNofQojIE1vdmUgYWxsIC50c3YgZmlsZXMgdG8gYSBjb21tb24gZGlyZWN0b3J5CmNwIC1SdiB+L1NDQU5CX1BVQkxJQ0FUSU9OL0RFX2FuYWx5c2lzL0RFX2Zvcl9kZWNpc2lvbnRyZWUvKi8qLnRzdiB+L1NDQU5CX1BVQkxJQ0FUSU9OL0RFX2FuYWx5c2lzL0RFX2Zvcl9kZWNpc2lvbnRyZWUKYGBgCgpgYGB7cn0KIyBMaXN0IG9mIGRpZmZlcmVudGlhbGx5IGFidW5kYW50IHByb3RlaW5zCkRFRmlsZXMgPC0gbGlzdC5maWxlcyhwYXR0ZXJuID0gJyoudHN2JyxwYXRoID0gJ0RFX2FuYWx5c2lzL0RFX2Zvcl9kZWNpc2lvbnRyZWUvJykKREVEYXRhIDwtIG1hcCgueCA9IERFRmlsZXMsLmYgPSB+cmVhZF90c3YoZmlsZSA9IHBhc3RlMCgnREVfYW5hbHlzaXMvREVfZm9yX2RlY2lzaW9udHJlZS8nLC54KSkpCm5hbWVzKERFRGF0YSkgPC0gREVGaWxlcwoKREVEYXRhRmlsdGVyIDwtIG1hcCgueCA9IERFRGF0YSwuZiA9IH5maWx0ZXIoLmRhdGEgPSAueCwueFtzdHJfZGV0ZWN0KHN0cmluZyA9IGNvbG5hbWVzKC54KSxwYXR0ZXJuID0gJ0FkalBWYWwnKV08MC4wMSkpICU+JSAKICBtYXAoLnggPSAuLC5mID0gfmZpbHRlcigueCxhYnMoLnhbc3RyX2RldGVjdChzdHJpbmcgPSBjb2xuYW1lcygueCkscGF0dGVybiA9ICdsb2cyRm9sZENoYW5nZScpXSk+PTIpKSAlPiUgCiAgbWFwKC54ID0gLiwuZiA9IH5wdWxsKC54LFByb3RlaW4pKSAlPiUgCiAgdW5saXN0ICU+JSAKICB1bmlxdWUKYGBgCmBgYHtyfQojIERhdGEgaW1wb3J0CmRhdGEgPC0gJ0RJQS9EYXRhREUvRElBTk4vRElBX0RJQU5OX1Byb3RlaW5fUlJvbGx1cC9ESUFfRElBTk5fUlJvbGx1cF9HZW5lcy50c3YnICU+JSAKICByZWFkX3RzdgpkZXNpZ24gPC0gICdESUEvRGVzaWduRmlsZXNESUEvZGVzaWduX2FpbTJfY2xpbmljYWxfRGVjaXNpb25UcmVlLnRzdicgJT4lIAogIHJlYWRfdHN2CmBgYApgYGB7cn0KIyBTZXR0aW5nIHVwIHRoZSBpbnB1dCBkYXRhIGZyYW1lCmRhdGFDb21wbGV0ZSA8LSBkYXRhICU+JSAKICBkcGx5cjo6c2VsZWN0KFByb3RlaW4sZGVzaWduJFBvc2l0aW9uKSAlPiUKICBmaWx0ZXIoUHJvdGVpbiAlaW4lIERFRGF0YUZpbHRlcikKcHJvdE5hbWVzIDwtIGRhdGFDb21wbGV0ZSRQcm90ZWluCgpkYXRhQ29tcGxldGUgPC0gZGF0YUNvbXBsZXRlICU+JSAKICBkcGx5cjo6c2VsZWN0KGRlc2lnbiRQb3NpdGlvbikgJT4lIAogIHQKCmNvbG5hbWVzKGRhdGFDb21wbGV0ZSkgPC0gcHJvdE5hbWVzCgpvdXRWYXIgPC0gZGVzaWduJEFJTVNfUEFNNTAgJT4lIGFzLmZhY3RvcgpkYXRhQ29tcGxldGUgPC0gZGF0YS5mcmFtZShkYXRhQ29tcGxldGUsT3V0VmFyPW91dFZhcixQb3NpdGlvbj1yb3duYW1lcyhkYXRhQ29tcGxldGUpKSAlPiUgCiAgZmlsdGVyKCFpcy5uYShPdXRWYXIpKQpgYGAKYGBge3J9CiMgU3BsaXR0aW5nIHRoZSBkYXRhIGluIDcwJSBmb3IgdHJhaW5pbmcgYW5kIDMwJSBmb3IgdGVzdGluZyBiYXNlZCBvbiB0aGUgaW50cmluc2ljIHN1YnR5cGVzCnRyYWluU2V0IDwtIGRhdGFDb21wbGV0ZSAlPiUgZ3JvdXBfYnkoT3V0VmFyKSAlPiUgc2FtcGxlX2ZyYWMoMC43KQoKZGlmZlJvd3MgPC0gc2V0ZGlmZihkZXNpZ24kUG9zaXRpb24sdHJhaW5TZXQkUG9zaXRpb24pCnRlc3RTZXQgPC0gZGF0YUNvbXBsZXRlW2RpZmZSb3dzLF0KCmRhdGFDb21wbGV0ZUNsZWFuIDwtIGRhdGFDb21wbGV0ZSAlPiUgCiAgZHBseXI6OnNlbGVjdCgtUG9zaXRpb24pCnRyYWluU2V0Q2xlYW4gPC0gdHJhaW5TZXQgJT4lIAogIGRwbHlyOjpzZWxlY3QoLVBvc2l0aW9uKQp0ZXN0U2V0Q2xlYW4gPC0gdGVzdFNldCAlPiUgCiAgZHBseXI6OnNlbGVjdCgtUG9zaXRpb24pCmBgYAoKIyMgTW9kZWwgVHJhaW5pbmcgYW5kIFBsb3R0aW5nCgojIyMgVHJhaW5pbmcKYGBge3J9CnNldC5zZWVkKDQzMjEpCnByb3RUcmVlTW9kZWwgPC0gdHJhaW4oT3V0VmFyIH4gLiwgZGF0YSA9IHRyYWluU2V0Q2xlYW4sIG1ldGhvZCA9ICJjdHJlZSIsCiAgICB0ckNvbnRyb2wgPSB0cmFpbkNvbnRyb2wobWV0aG9kID0gImJvb3QiKSxuYS5hY3Rpb24gPSBuYS5wYXNzKQpwbG90KHByb3RUcmVlTW9kZWwkZmluYWxNb2RlbCkgI0ZpZ3VyZSAyCmBgYAoKIyMjIFBsb3R0aW5nCmBgYHtyfQpwbmcoZmlsZW5hbWUgPSAnZGVjaXNpb25fdHJlZXMvRmlndXJlMi5wbmcnLHdpZHRoID0gMTksaGVpZ2h0ID0gOSx1bml0cyA9ICdpbicscmVzID0gNjAwKQpwbG90KHByb3RUcmVlTW9kZWwkZmluYWxNb2RlbCkKZGV2Lm9mZigpCmBgYAoKIyMgTW9kZWwgVGVzdGluZwpgYGB7cn0KcHJlZFNjYW5iIDwtIHByZWRpY3QocHJvdFRyZWVNb2RlbCx0ZXN0U2V0Q2xlYW4sbmEuYWN0aW9uID0gbmEucGFzcykKY29uZnVzaW9uTWF0cml4KHRhYmxlKHRlc3RTZXQkT3V0VmFyLHByZWRTY2FuYikpCnNhdmUuaW1hZ2UoZmlsZSA9ICdkZWNpc2lvbl90cmVlcy9tb2RlbERhdGEuUkRhdGEnLHNhZmUgPSBUUlVFKQpgYGAKCgoKIyMgRGVjaXNpb24gVHJlZSBmcm9tIHByb3RlaW5zIGZvdW5kIGluIEJvdWNoYWwgUC4sIGV0LiBhbC4sICgyMDE5KQpgYGB7cn0KbG9hZChmaWxlID0gJ2RlY2lzaW9uX3RyZWVzL21vZGVsRGF0YS5SRGF0YScpCkVSQkIyIDwtICdQMDQ2MjYnCklOUFA0QiA8LSAnTzE1MzI3JwpDREsxIDwtICdQMDY0OTMnCnRyYWluU3dhdGggPC0gdHJhaW5TZXRDbGVhbiAlPiUgCiAgZHBseXI6OnNlbGVjdChFUkJCMixJTlBQNEIsQ0RLMSxPdXRWYXIpCnRlc3RTd2F0aCA8LSB0ZXN0U2V0Q2xlYW4gJT4lIAogIGRwbHlyOjpzZWxlY3QoRVJCQjIsSU5QUDRCLENESzEsT3V0VmFyKQoKc2V0LnNlZWQoNDMyMSkKZml0U3dhdGggPC0gdHJhaW4oT3V0VmFyIH4gLiwgZGF0YSA9IHRyYWluU3dhdGgsIG1ldGhvZCA9ICJjdHJlZSIsCiAgICAgICAgICAgICAgdHJDb250cm9sID0gdHJhaW5Db250cm9sKG1ldGhvZCA9ICJib290IiksbmEuYWN0aW9uID0gbmEucGFzcykKcGxvdChmaXRTd2F0aCRmaW5hbE1vZGVsKSAjRmlndXJlIDMKYGBgCgojIFVuc3VwZXJ2aXNlZCBIaWVyYXJjaGljYWwgQ2x1c3RlcmluZyAoRklHVVJFIDQgQU5EIFNVUFBMRU1FTlRBUlkgRklHVVJFUyBTMS0yKQoKIyMgTG9hZGluZyBEYXRhCmBgYHtyfQojIFByb3Rlb21pY3MgRGF0YQpwcm90ZWluRGF0YSA8LSAnRElBL0RhdGFERS9ESUFOTi9ESUFfRElBTk5fUHJvdGVpbl9SUm9sbHVwL0RJQV9ESUFOTl9SUm9sbHVwX0dlbmVzLnRzdicgJT4lIAogIHJlYWRfdHN2ICU+JSAKICBkcm9wX25hCmRlc2lnbiA8LSAnRElBL0Rlc2lnbkZpbGVzRElBL2Rlc2lnbl9haW0yX2NsaW5pY2FsX21hdGNoZWRfaW5kaXZpZHVhbHN1YnR5cGVzLnRzdicgJT4lIAogIHJlYWRfdHN2CmBgYApgYGB7cn0KIyBUcmFuc2NyaXB0b21pY3MgRGF0YQpybmFEYXRhIDwtICdSTkEtc2VxL0dTRTk2MDU4X2dlbmVfZXhwcmVzc2lvbl9SZWR1Y2VkLnRzdicgJT4lIAogIHJlYWRfdHN2ICU+JSAKICBkcm9wX25hCmBgYAojIyBDbHVzdGVyaW5nIGFmdGVyIGxvdyB2YXJpYW5jZSBmaWx0ZXJpbmcKIyMjIE1hdGNoaW5nIHNhbXBsZXMKYGBge3J9CnByb3RlaW5EYXRhTWF0Y2hlZCA8LSBwcm90ZWluRGF0YSAlPiUgCiAgZHBseXI6OnNlbGVjdChHZW5lcyxkZXNpZ24kUG9zaXRpb24pCnJuYURhdGFNYXRjaGVkIDwtIHJuYURhdGEgJT4lIAogIGRwbHlyOjpzZWxlY3QoR2VuZSxkZXNpZ24kU2FtcGxlX3RpdGxlKQpgYGAKCiMjIyBGdW5jdGlvbiB0byByZW1vdmUgbG93IHZhcmlhbmNlIGZlYXR1cmVzCmBgYHtyfQptZWRpYW5GaWx0ZXJpbmcgPC0gZnVuY3Rpb24oZGF0YSxkZXNpZ24sc2FtcGxlQ29sLGFubm90YXRpb24sLi4uKXsKICB2YXJpYW5jZU5BIDwtIGZ1bmN0aW9uKGRhdGEpIHZhcihkYXRhLG5hLnJtPVRSVUUpCiAgCiAgcm93TmFtZSA8LSBwdWxsKGRhdGEsYW5ub3RhdGlvbikKICB2YXJpYW5jZURhdGEgPC0gZGF0YSAlPiUgCiAgICBkcGx5cjo6c2VsZWN0KC4scHVsbChkZXNpZ24sc2FtcGxlQ29sKSkgJT4lIAogICAgdCAlPiUgCiAgICBhc190aWJibGUgJT4lIAogICAgbWFwX2RmYyguLHZhcmlhbmNlTkEpICU+JSAKICAgIHQKICAKICBkYXRhVmFyIDwtIGRhdGEgJT4lIAogICAgbXV0YXRlKC4sdmFyaWFuY2U9dmFyaWFuY2VEYXRhLFByb3RlaW49cm93TmFtZSkKICAKICBsb3dWYXJEYXRhIDwtIGRhdGFWYXIgJT4lIAogICAgZmlsdGVyKHZhcmlhbmNlID4gbWVkaWFuKHZhcmlhbmNlLG5hLnJtID0gVCkpCiAgCiAgcmV0dXJuKGxvd1ZhckRhdGEpCn0KYGBgCgojIyMgQ2x1c3RlcmluZyB3aXRoIGFubm90YXRpb25zCgojIyMjIERJQS1OTgpgYGB7cn0KcHJvdGVpbkRhdGFGaWx0ZXJlZCA8LSBtZWRpYW5GaWx0ZXJpbmcoZGF0YSA9IHByb3RlaW5EYXRhTWF0Y2hlZCxkZXNpZ24gPSBkZXNpZ24sc2FtcGxlQ29sID0gJ1Bvc2l0aW9uJyxhbm5vdGF0aW9uID0gJ0dlbmVzJykKCnByb3RlaW5EYXRhUmVkdWNlZCA8LSBwcm90ZWluRGF0YUZpbHRlcmVkICU+JSAKICBkcGx5cjo6c2VsZWN0KGRlc2lnbiRQb3NpdGlvbikKCiMgQ2FsY3VsYXRpb24gb2YgWi1TY29yZXMKY2FsWlNjb3JlIDwtIGZ1bmN0aW9uKHgpICh4LW1lYW4oeCkpL3NkKHgpCgpwcm90ZWluZGF0YU5vcm0gPC0gdChhcHBseShwcm90ZWluRGF0YVJlZHVjZWQsMSxjYWxaU2NvcmUpKQpjb2xuYW1lcyhwcm90ZWluZGF0YU5vcm0pIDwtIE5VTEwKCmFubm90YXRpb25Db2x1bW4gPC0gSGVhdG1hcEFubm90YXRpb24oUEFNNTA9YW5ub19zaW1wbGUoeCA9IGRlc2lnbiRQQU01MCwKICAgICAgICAgICAgICAgICAgICAgICAgICAgICAgICAgICAgICAgICAgICAgICAgICAgICAgICBjb2wgPSBjKCdBSU1TX1BBTTUwLkJhc2FsJz0nI2UzMWExYycsJ0FJTVNfUEFNNTAuSGVyMic9JyNmYjlhOTknLCdBSU1TX1BBTTUwLkx1bUEnPScjMWY3OGI0JywnQUlNU19QQU01MC5MdW1CJz0nI2E2Y2VlMycsJ0FJTVNfUEFNNTAuTm9ybWFsJz0nIzMzYTAyYycpLAogICAgICAgICAgICAgICAgICAgICAgICAgICAgICAgICAgICAgICAgICAgICAgICAgICAgICAgIG5hX2NvbCA9ICcjODA4MDgwJywKICAgICAgICAgICAgICAgICAgICAgICAgICAgICAgICAgICAgICAgICAgICAgICAgICAgICAgICBncCA9IGdwYXIodGl0bGU9J1BBTTUwJykpLAogICAgICAgICAgICAgICAgICAgICAgICAgICAgICAgICAgICAgIE5vZGVTdGF0dXM9YW5ub19zaW1wbGUoeCA9IGRlc2lnbiRgTE4gc3RhdHVzYCwKICAgICAgICAgICAgICAgICAgICAgICAgICAgICAgICAgICAgICAgICAgICAgICAgICAgICAgICAgICAgIGNvbCA9IGMoJ05vZGVQb3NpdGl2ZSc9JyMwMDAwMDAnLCdOb2RlTmVnYXRpdmUnPScjZmZmZmZmJyksCiAgICAgICAgICAgICAgICAgICAgICAgICAgICAgICAgICAgICAgICAgICAgICAgICAgICAgICAgICAgICBuYV9jb2wgPSAnIzgwODA4MCcsCiAgICAgICAgICAgICAgICAgICAgICAgICAgICAgICAgICAgICAgICAgICAgICAgICAgICAgICAgICAgICBncCA9IGdwYXIodGl0bGU9J05vZGVTdGF0dXMnKSksCiAgICAgICAgICAgICAgICAgICAgICAgICAgICAgICAgICAgICAgSEVSMj1hbm5vX3NpbXBsZSh4ID0gZGVzaWduJGBIRVIyIHN0YXR1c2AsCiAgICAgICAgICAgICAgICAgICAgICAgICAgICAgICAgICAgICAgICAgICAgICAgICAgICAgICBjb2wgPSBjKCdQb3MnPScjMDAwMDAwJywnTmVnJz0nI2ZmZmZmZicpLAogICAgICAgICAgICAgICAgICAgICAgICAgICAgICAgICAgICAgICAgICAgICAgICAgICAgICAgbmFfY29sID0gJyM4MDgwODAnLAogICAgICAgICAgICAgICAgICAgICAgICAgICAgICAgICAgICAgICAgICAgICAgICAgICAgICAgZ3AgPSBncGFyKHRpdGxlPSdIRVIyJykpLAogICAgICAgICAgICAgICAgICAgICAgICAgICAgICAgICAgICAgIEVSPWFubm9fc2ltcGxlKHggPSBkZXNpZ24kYEVSIHN0YXR1c2AsCiAgICAgICAgICAgICAgICAgICAgICAgICAgICAgICAgICAgICAgICAgICAgICAgICAgICAgY29sID0gYygnUG9zJz0nIzAwMDAwMCcsJ05lZyc9JyNmZmZmZmYnKSwKICAgICAgICAgICAgICAgICAgICAgICAgICAgICAgICAgICAgICAgICAgICAgICAgICAgICBuYV9jb2wgPSAnIzgwODA4MCcsCiAgICAgICAgICAgICAgICAgICAgICAgICAgICAgICAgICAgICAgICAgICAgICAgICAgICAgZ3AgPSBncGFyKHRpdGxlPSdFUicpKSwKICAgICAgICAgICAgICAgICAgICAgICAgICAgICAgICAgICAgICBQUj1hbm5vX3NpbXBsZSh4ID0gZGVzaWduJGBQUiBzdGF0dXNgLAogICAgICAgICAgICAgICAgICAgICAgICAgICAgICAgICAgICAgICAgICAgICAgICAgICAgICBjb2wgPSBjKCdQb3MnPScjMDAwMDAwJywnTmVnJz0nI2ZmZmZmZicpLAogICAgICAgICAgICAgICAgICAgICAgICAgICAgICAgICAgICAgICAgICAgICAgICAgICAgICBuYV9jb2wgPSAnIzgwODA4MCcsCiAgICAgICAgICAgICAgICAgICAgICAgICAgICAgICAgICAgICAgICAgICAgICAgICAgICAgIGdwID0gZ3Bhcih0aXRsZT0nUFInKSkpCgpsZWdlbmRQQU01MCA8LSBMZWdlbmQobGFiZWxzID0gYygnQmFzYWwnLCdIZXIyJywnTHVtQScsJ0x1bUInLCdOb3JtYWwnKSwKICAgICAgICAgICAgICAgICAgICAgIHRpdGxlID0gJ1BBTTUwIHN1YnR5cGUnLAogICAgICAgICAgICAgICAgICAgICAgYm9yZGVyID0gJ2JsYWNrJywKICAgICAgICAgICAgICAgICAgICAgIGxlZ2VuZF9ncCA9IGdwYXIoZmlsbD1jKCdCYXNhbCc9JyNlMzFhMWMnLCdIZXIyJz0nI2ZiOWE5OScsJ0x1bUEnPScjMWY3OGI0JywnTHVtQic9JyNhNmNlZTMnLCdOb3JtYWwnPScjMzNhMDJjJykpKQpsZWdlbmRIZXIyIDwtIExlZ2VuZChsYWJlbHMgPSBjKCdQb3NpdGl2ZScsJ05lZ2F0aXZlJywnTkEnKSwKICAgICAgICAgICAgICAgICAgICAgdGl0bGUgPSAnSEVSMiBzdGF0dXMnLAogICAgICAgICAgICAgICAgICAgICBib3JkZXIgPSAnYmxhY2snLAogICAgICAgICAgICAgICAgICAgICBsZWdlbmRfZ3AgPSBncGFyKGZpbGw9YygnUG9zaXRpdmUnPScjMDAwMDAwJywnTmVnYXRpdmUnPScjZmZmZmZmJywnTkEnPScjODA4MDgwJykpKQpsZWdlbmROb2RlU3RhdHVzIDwtIExlZ2VuZChsYWJlbHMgPSBjKCdQb3NpdGl2ZScsJ05lZ2F0aXZlJyksCiAgICAgICAgICAgICAgICAgICAgICAgICAgIHRpdGxlID0gJ05vZGUgc3RhdHVzJywKICAgICAgICAgICAgICAgICAgICAgICAgICAgYm9yZGVyID0gJ2JsYWNrJywKICAgICAgICAgICAgICAgICAgICAgICAgICAgbGVnZW5kX2dwID0gZ3BhcihmaWxsPWMoJ1Bvc2l0aXZlJz0nIzAwMDAwMCcsJ05lZ2F0aXZlJz0nI2ZmZmZmZicpKSkKbGVnZW5kRVIgPC0gTGVnZW5kKGxhYmVscyA9IGMoJ1Bvc2l0aXZlJywnTmVnYXRpdmUnKSwKICAgICAgICAgICAgICAgICAgIHRpdGxlID0gJ0VSIHN0YXR1cycsCiAgICAgICAgICAgICAgICAgICBib3JkZXIgPSAnYmxhY2snLAogICAgICAgICAgICAgICAgICAgbGVnZW5kX2dwID0gZ3BhcihmaWxsPWMoJ1Bvc2l0aXZlJz0nIzAwMDAwMCcsJ05lZ2F0aXZlJz0nI2ZmZmZmZicpKSkKbGVnZW5kUFIgPC0gTGVnZW5kKGxhYmVscyA9IGMoJ1Bvc2l0aXZlJywnTmVnYXRpdmUnKSwKICAgICAgICAgICAgICAgICAgICB0aXRsZSA9ICdQUiBzdGF0dXMnLAogICAgICAgICAgICAgICAgICAgIGJvcmRlciA9ICdibGFjaycsCiAgICAgICAgICAgICAgICAgICAgbGVnZW5kX2dwID0gZ3BhcihmaWxsPWMoJ1Bvc2l0aXZlJz0nIzAwMDAwMCcsJ05lZ2F0aXZlJz0nI2ZmZmZmZicpKSkKCmFsbExlZ2VuZHMgPC0gcGFja0xlZ2VuZChsZWdlbmRQQU01MCxsZWdlbmRIZXIyLGxlZ2VuZE5vZGVTdGF0dXMsbGVnZW5kRVIsbGVnZW5kUFIpCnByb3RlaW5IZWF0bWFwIDwtIEhlYXRtYXAobWF0cml4ID0gcHJvdGVpbmRhdGFOb3JtLHRvcF9hbm5vdGF0aW9uID0gYW5ub3RhdGlvbkNvbHVtbiwgbmFtZSA9ICdQcm90ZWluIGludGVuc2l0eScsaGVhdG1hcF9sZWdlbmRfcGFyYW0gPSBsaXN0KGxlZ2VuZF9kaXJlY3Rpb24gPSAnaG9yaXpvbnRhbCcpKQoKcHJvdGVpbkhlYXRtYXBEcmF3IDwtIGdyaWQuZ3JhYkV4cHIoZHJhdyhwcm90ZWluSGVhdG1hcCxhbm5vdGF0aW9uX2xlZ2VuZF9saXN0ID0gYWxsTGVnZW5kcywgaGVhdG1hcF9sZWdlbmRfc2lkZSA9ICdib3R0b20nLCBhbm5vdGF0aW9uX2xlZ2VuZF9zaWRlID0gJ3JpZ2h0JywgbGVnZW5kX2dyb3VwaW5nID0gJ29yaWdpbmFsJykpICU+JSBhcy5nZ3Bsb3QKCmdnc2F2ZTIoZmlsZW5hbWUgPSAnc3VwcGxlbWVudGFyeV9maWd1cmVTMUEucGRmJyxwbG90ID0gcHJvdGVpbkhlYXRtYXBEcmF3LGRldmljZSA9ICdwZGYnLHBhdGggPSAnY2x1c3RlcmluZy8nLGRwaSA9IDYwMCx3aWR0aCA9IDEwLGhlaWdodCA9IDEwLHVuaXRzID0gJ2luJykKYGBgCgojIyMjIFJOQS1zZXEKYGBge3J9CnJuYURhdGFGaWx0ZXJlZCA8LSBtZWRpYW5GaWx0ZXJpbmcoZGF0YSA9IHJuYURhdGFNYXRjaGVkLGRlc2lnbiA9IGRlc2lnbixzYW1wbGVDb2wgPSAnU2FtcGxlX3RpdGxlJyxhbm5vdGF0aW9uID0gJ0dlbmUnKQoKcm5hRGF0YVJlZHVjZWQgPC0gcm5hRGF0YUZpbHRlcmVkICU+JSAKICBkcGx5cjo6c2VsZWN0KGRlc2lnbiRTYW1wbGVfdGl0bGUpCgpybmFEYXRhTm9ybSA8LSB0KGFwcGx5KHJuYURhdGFSZWR1Y2VkLDEsY2FsWlNjb3JlKSkKY29sbmFtZXMocm5hRGF0YU5vcm0pIDwtIE5VTEwKCnJuYUhlYXRtYXAgPC0gSGVhdG1hcChtYXRyaXggPSBybmFEYXRhTm9ybSx0b3BfYW5ub3RhdGlvbiA9IGFubm90YXRpb25Db2x1bW4sIG5hbWUgPSAnR2VuZSBleHByZXNzaW9uJyxoZWF0bWFwX2xlZ2VuZF9wYXJhbSA9IGxpc3QobGVnZW5kX2RpcmVjdGlvbiA9ICdob3Jpem9udGFsJykpCnJuYUhlYXRtYXBEcmF3IDwtIGdyaWQuZ3JhYkV4cHIoZHJhdyhybmFIZWF0bWFwLGFubm90YXRpb25fbGVnZW5kX2xpc3QgPSBhbGxMZWdlbmRzLCBoZWF0bWFwX2xlZ2VuZF9zaWRlID0gJ2JvdHRvbScsIGFubm90YXRpb25fbGVnZW5kX3NpZGUgPSAncmlnaHQnLCBsZWdlbmRfZ3JvdXBpbmcgPSAnb3JpZ2luYWwnKSkgJT4lIGFzLmdncGxvdAoKZ2dzYXZlMihmaWxlbmFtZSA9ICdzdXBwbGVtZW50YXJ5X2ZpZ3VyZVMxQi5wZGYnLHBsb3QgPSBybmFIZWF0bWFwRHJhdyxkZXZpY2UgPSAncGRmJyxwYXRoID0gJ2NsdXN0ZXJpbmcvJyxkcGkgPSA2MDAsIHdpZHRoID0gMTAsaGVpZ2h0ID0gMTAsdW5pdHMgPSAnaW4nKQpgYGAKCiMjIyMgSm9pbnQgUGxvdHMKYGBge3J9Cmh0UHJvdCA8LSBwcm90ZWluSGVhdG1hcERyYXcKaHRSTkEgPC0gZ3JpZC5ncmFiRXhwcihkcmF3KHJuYUhlYXRtYXAsaGVhdG1hcF9sZWdlbmRfc2lkZSA9ICdib3R0b20nKSkgJT4lIGFzLmdncGxvdApqb2ludFBsb3RWYXIgPC0gcGxvdF9ncmlkKGh0UHJvdCxodFJOQSxsYWJlbHMgPSBMRVRURVJTWzE6Ml0pCmdnc2F2ZTIoZmlsZW5hbWUgPSAnc3VwcGxlbWVudGFyeV9maWd1cmVTMS5wZGYnLHBsb3QgPSBqb2ludFBsb3RWYXIsZGV2aWNlID0gJ3BkZicscGF0aCA9ICdjbHVzdGVyaW5nLycsZHBpID0gNjAwLHdpZHRoID0gMTUsaGVpZ2h0ID0gMTAsdW5pdHMgPSAnaW4nKQpgYGAKCiMjIENsdXN0ZXJpbmcgd2l0aCBmZWF0dXJlcyBmcm9tIERlY2lzaW9uIFRyZWUKCiMjIyBMaXN0IG9mIGRpZmZlcmVudGlhbGx5IGFidW5kYW50IGZlYXR1cmVzCmBgYHtyfQpERUZpbGVzIDwtIGxpc3QuZmlsZXMocGF0dGVybiA9ICcqLnRzdicscGF0aCA9ICdERV9hbmFseXNpcy9ERV9mb3JfZGVjaXNpb250cmVlLycpCkRFRGF0YSA8LSBtYXAoLnggPSBERUZpbGVzLC5mID0gfnJlYWRfdHN2KGZpbGUgPSBwYXN0ZTAoJ0RFX2FuYWx5c2lzL0RFX2Zvcl9kZWNpc2lvbnRyZWUvJywueCkpKQpuYW1lcyhERURhdGEpIDwtIERFRmlsZXMKCkRFRGF0YUZpbHRlciA8LSBtYXAoLnggPSBERURhdGEsLmYgPSB+ZmlsdGVyKC5kYXRhID0gLngsLnhbc3RyX2RldGVjdChzdHJpbmcgPSBjb2xuYW1lcygueCkscGF0dGVybiA9ICdBZGpQVmFsJyldPDAuMDEpKSAlPiUgCiAgbWFwKC54ID0gLiwuZiA9IH5maWx0ZXIoLngsYWJzKC54W3N0cl9kZXRlY3Qoc3RyaW5nID0gY29sbmFtZXMoLngpLHBhdHRlcm4gPSAnbG9nMkZvbGRDaGFuZ2UnKV0pPj0yKSkgJT4lIAogIG1hcCgueCA9IC4sLmYgPSB+cHVsbCgueCxQcm90ZWluKSkgJT4lIAogIHVubGlzdCAlPiUgCiAgdW5pcXVlCmBgYAoKIyMjIEZpbHRlciBEYXRhCmBgYHtyfQpwcm90RGF0YVRyZWUgPC0gcHJvdGVpbkRhdGEgJT4lCiAgZmlsdGVyKFByb3RlaW4gJWluJSBERURhdGFGaWx0ZXIpICU+JSAKICBkcm9wX25hCgpnZW5lTmFtZXMgPC0gcHJvdERhdGFUcmVlJEdlbmVzCgpybmFEYXRhVHJlZSA8LSBybmFEYXRhICU+JSAKICBmaWx0ZXIoR2VuZSAlaW4lIGdlbmVOYW1lcykgJT4lIAogIGRyb3BfbmEKYGBgCgojIyMgTWF0Y2hpbmcgRGF0YQpgYGB7cn0KcHJvdFRyZWVNYXRjaCA8LSBwcm90RGF0YVRyZWUgJT4lIAogIGRwbHlyOjpzZWxlY3QoR2VuZXMsZGVzaWduJFBvc2l0aW9uKQpybmFUcmVlTWF0Y2ggPC0gcm5hRGF0YVRyZWUgJT4lIAogIGRwbHlyOjpzZWxlY3QoR2VuZSxkZXNpZ24kU2FtcGxlX3RpdGxlKQpgYGAKCiMjIyBDbHVzdGVyaW5nCgojIyMjIERJQS1OTgpgYGB7cn0KcHJvdFRyZWVSZWR1Y2VkIDwtIHByb3RUcmVlTWF0Y2ggJT4lIAogIGRwbHlyOjpzZWxlY3QoZGVzaWduJFBvc2l0aW9uKQoKIyBDYWxjdWxhdGlvbiBvZiBaLVNjb3JlcwpjYWxaU2NvcmUgPC0gZnVuY3Rpb24oeCkgKHgtbWVhbih4KSkvc2QoeCkKCnByb3RUcmVlTm9ybSA8LSB0KGFwcGx5KHByb3RUcmVlUmVkdWNlZCwxLGNhbFpTY29yZSkpCmNvbG5hbWVzKHByb3RUcmVlTm9ybSkgPC0gTlVMTApyb3duYW1lcyhwcm90VHJlZU5vcm0pIDwtIHByb3RUcmVlTWF0Y2gkR2VuZXMKCmFubm90YXRpb25Db2x1bW4gPC0gSGVhdG1hcEFubm90YXRpb24oUEFNNTA9YW5ub19zaW1wbGUoeCA9IGRlc2lnbiRQQU01MCwKICAgICAgICAgICAgICAgICAgICAgICAgICAgICAgICAgICAgICAgICAgICAgICAgICAgICAgICBjb2wgPSBjKCdBSU1TX1BBTTUwLkJhc2FsJz0nI2UzMWExYycsJ0FJTVNfUEFNNTAuSGVyMic9JyNmYjlhOTknLCdBSU1TX1BBTTUwLkx1bUEnPScjMWY3OGI0JywnQUlNU19QQU01MC5MdW1CJz0nI2E2Y2VlMycsJ0FJTVNfUEFNNTAuTm9ybWFsJz0nIzMzYTAyYycpLAogICAgICAgICAgICAgICAgICAgICAgICAgICAgICAgICAgICAgICAgICAgICAgICAgICAgICAgIG5hX2NvbCA9ICcjODA4MDgwJywKICAgICAgICAgICAgICAgICAgICAgICAgICAgICAgICAgICAgICAgICAgICAgICAgICAgICAgICBncCA9IGdwYXIodGl0bGU9J1BBTTUwJykpLAogICAgICAgICAgICAgICAgICAgICAgICAgICAgICAgICAgICAgIE5vZGVTdGF0dXM9YW5ub19zaW1wbGUoeCA9IGRlc2lnbiRgTE4gc3RhdHVzYCwKICAgICAgICAgICAgICAgICAgICAgICAgICAgICAgICAgICAgICAgICAgICAgICAgICAgICAgICAgICAgIGNvbCA9IGMoJ05vZGVQb3NpdGl2ZSc9JyMwMDAwMDAnLCdOb2RlTmVnYXRpdmUnPScjZmZmZmZmJyksCiAgICAgICAgICAgICAgICAgICAgICAgICAgICAgICAgICAgICAgICAgICAgICAgICAgICAgICAgICAgICBuYV9jb2wgPSAnIzgwODA4MCcsCiAgICAgICAgICAgICAgICAgICAgICAgICAgICAgICAgICAgICAgICAgICAgICAgICAgICAgICAgICAgICBncCA9IGdwYXIodGl0bGU9J05vZGVTdGF0dXMnKSksCiAgICAgICAgICAgICAgICAgICAgICAgICAgICAgICAgICAgICAgSEVSMj1hbm5vX3NpbXBsZSh4ID0gZGVzaWduJGBIRVIyIHN0YXR1c2AsCiAgICAgICAgICAgICAgICAgICAgICAgICAgICAgICAgICAgICAgICAgICAgICAgICAgICAgICBjb2wgPSBjKCdQb3MnPScjMDAwMDAwJywnTmVnJz0nI2ZmZmZmZicpLAogICAgICAgICAgICAgICAgICAgICAgICAgICAgICAgICAgICAgICAgICAgICAgICAgICAgICAgbmFfY29sID0gJyM4MDgwODAnLAogICAgICAgICAgICAgICAgICAgICAgICAgICAgICAgICAgICAgICAgICAgICAgICAgICAgICAgZ3AgPSBncGFyKHRpdGxlPSdIRVIyJykpLAogICAgICAgICAgICAgICAgICAgICAgICAgICAgICAgICAgICAgIEVSPWFubm9fc2ltcGxlKHggPSBkZXNpZ24kYEVSIHN0YXR1c2AsCiAgICAgICAgICAgICAgICAgICAgICAgICAgICAgICAgICAgICAgICAgICAgICAgICAgICAgY29sID0gYygnUG9zJz0nIzAwMDAwMCcsJ05lZyc9JyNmZmZmZmYnKSwKICAgICAgICAgICAgICAgICAgICAgICAgICAgICAgICAgICAgICAgICAgICAgICAgICAgICBuYV9jb2wgPSAnIzgwODA4MCcsCiAgICAgICAgICAgICAgICAgICAgICAgICAgICAgICAgICAgICAgICAgICAgICAgICAgICAgZ3AgPSBncGFyKHRpdGxlPSdFUicpKSwKICAgICAgICAgICAgICAgICAgICAgICAgICAgICAgICAgICAgICBQUj1hbm5vX3NpbXBsZSh4ID0gZGVzaWduJGBQUiBzdGF0dXNgLAogICAgICAgICAgICAgICAgICAgICAgICAgICAgICAgICAgICAgICAgICAgICAgICAgICAgICBjb2wgPSBjKCdQb3MnPScjMDAwMDAwJywnTmVnJz0nI2ZmZmZmZicpLAogICAgICAgICAgICAgICAgICAgICAgICAgICAgICAgICAgICAgICAgICAgICAgICAgICAgICBuYV9jb2wgPSAnIzgwODA4MCcsCiAgICAgICAgICAgICAgICAgICAgICAgICAgICAgICAgICAgICAgICAgICAgICAgICAgICAgIGdwID0gZ3Bhcih0aXRsZT0nUFInKSkpCgpsZWdlbmRQQU01MCA8LSBMZWdlbmQobGFiZWxzID0gYygnQmFzYWwnLCdIZXIyJywnTHVtQScsJ0x1bUInLCdOb3JtYWwnKSwKICAgICAgICAgICAgICAgICAgICAgIHRpdGxlID0gJ1BBTTUwIHN1YnR5cGUnLAogICAgICAgICAgICAgICAgICAgICAgYm9yZGVyID0gJ2JsYWNrJywKICAgICAgICAgICAgICAgICAgICAgIGxlZ2VuZF9ncCA9IGdwYXIoZmlsbD1jKCdCYXNhbCc9JyNlMzFhMWMnLCdIZXIyJz0nI2ZiOWE5OScsJ0x1bUEnPScjMWY3OGI0JywnTHVtQic9JyNhNmNlZTMnLCdOb3JtYWwnPScjMzNhMDJjJykpKQpsZWdlbmRIZXIyIDwtIExlZ2VuZChsYWJlbHMgPSBjKCdQb3NpdGl2ZScsJ05lZ2F0aXZlJywnTkEnKSwKICAgICAgICAgICAgICAgICAgICAgdGl0bGUgPSAnSEVSMiBzdGF0dXMnLAogICAgICAgICAgICAgICAgICAgICBib3JkZXIgPSAnYmxhY2snLAogICAgICAgICAgICAgICAgICAgICBsZWdlbmRfZ3AgPSBncGFyKGZpbGw9YygnUG9zaXRpdmUnPScjMDAwMDAwJywnTmVnYXRpdmUnPScjZmZmZmZmJywnTkEnPScjODA4MDgwJykpKQpsZWdlbmROb2RlU3RhdHVzIDwtIExlZ2VuZChsYWJlbHMgPSBjKCdQb3NpdGl2ZScsJ05lZ2F0aXZlJyksCiAgICAgICAgICAgICAgICAgICAgICAgICAgIHRpdGxlID0gJ05vZGUgc3RhdHVzJywKICAgICAgICAgICAgICAgICAgICAgICAgICAgYm9yZGVyID0gJ2JsYWNrJywKICAgICAgICAgICAgICAgICAgICAgICAgICAgbGVnZW5kX2dwID0gZ3BhcihmaWxsPWMoJ1Bvc2l0aXZlJz0nIzAwMDAwMCcsJ05lZ2F0aXZlJz0nI2ZmZmZmZicpKSkKbGVnZW5kRVIgPC0gTGVnZW5kKGxhYmVscyA9IGMoJ1Bvc2l0aXZlJywnTmVnYXRpdmUnKSwKICAgICAgICAgICAgICAgICAgIHRpdGxlID0gJ0VSIHN0YXR1cycsCiAgICAgICAgICAgICAgICAgICBib3JkZXIgPSAnYmxhY2snLAogICAgICAgICAgICAgICAgICAgbGVnZW5kX2dwID0gZ3BhcihmaWxsPWMoJ1Bvc2l0aXZlJz0nIzAwMDAwMCcsJ05lZ2F0aXZlJz0nI2ZmZmZmZicpKSkKbGVnZW5kUFIgPC0gTGVnZW5kKGxhYmVscyA9IGMoJ1Bvc2l0aXZlJywnTmVnYXRpdmUnKSwKICAgICAgICAgICAgICAgICAgICB0aXRsZSA9ICdQUiBzdGF0dXMnLAogICAgICAgICAgICAgICAgICAgIGJvcmRlciA9ICdibGFjaycsCiAgICAgICAgICAgICAgICAgICAgbGVnZW5kX2dwID0gZ3BhcihmaWxsPWMoJ1Bvc2l0aXZlJz0nIzAwMDAwMCcsJ05lZ2F0aXZlJz0nI2ZmZmZmZicpKSkKCmFsbExlZ2VuZHMgPC0gcGFja0xlZ2VuZChsZWdlbmRQQU01MCxsZWdlbmRIZXIyLGxlZ2VuZE5vZGVTdGF0dXMsbGVnZW5kRVIsbGVnZW5kUFIpCgpwcm90VHJlZUhlYXRtYXAgPC0gSGVhdG1hcChtYXRyaXggPSBwcm90VHJlZU5vcm0sdG9wX2Fubm90YXRpb24gPSBhbm5vdGF0aW9uQ29sdW1uLG5hbWUgPSAnUHJvdGVpbiBpbnRlbnNpdHknLCBoZWF0bWFwX2xlZ2VuZF9wYXJhbSA9IGxpc3QobGVnZW5kX2RpcmVjdGlvbiA9ICdob3Jpem9udGFsJykpCgpwcm90VHJlZUhlYXRtYXBEcmF3IDwtIGdyaWQuZ3JhYkV4cHIoZHJhdyhwcm90VHJlZUhlYXRtYXAsYW5ub3RhdGlvbl9sZWdlbmRfbGlzdCA9IGFsbExlZ2VuZHMsIGhlYXRtYXBfbGVnZW5kX3NpZGUgPSAnYm90dG9tJywgYW5ub3RhdGlvbl9sZWdlbmRfc2lkZSA9ICdyaWdodCcsIGxlZ2VuZF9ncm91cGluZyA9ICdvcmlnaW5hbCcpKSAlPiUgYXMuZ2dwbG90CgpnZ3NhdmUyKGZpbGVuYW1lID0gJ2ZpZ3VyZTRBLnBkZicscGxvdCA9IHByb3RUcmVlSGVhdG1hcERyYXcsZGV2aWNlID0gJ3BkZicscGF0aCA9ICdjbHVzdGVyaW5nLycsZHBpID0gNjAwLHdpZHRoID0gMTAsaGVpZ2h0ID0gMTAsdW5pdHMgPSAnaW4nKQpgYGAKCiMjIyMgUk5BLXNlcQpgYGB7cn0Kcm5hVHJlZVJlZHVjZWQgPC0gcm5hVHJlZU1hdGNoICU+JSAKICBkcGx5cjo6c2VsZWN0KGRlc2lnbiRTYW1wbGVfdGl0bGUpCgpybmFUcmVlTm9ybSA8LSB0KGFwcGx5KHJuYVRyZWVSZWR1Y2VkLDEsY2FsWlNjb3JlKSkKY29sbmFtZXMocm5hVHJlZU5vcm0pIDwtIE5VTEwKcm93bmFtZXMocm5hVHJlZU5vcm0pIDwtIHJuYVRyZWVNYXRjaCRHZW5lCgpybmFUcmVlSGVhdG1hcCA8LSBIZWF0bWFwKG1hdHJpeCA9IHJuYVRyZWVOb3JtLHRvcF9hbm5vdGF0aW9uID0gYW5ub3RhdGlvbkNvbHVtbiwgbmFtZSA9ICdHZW5lIGV4cHJlc3Npb24nLCBoZWF0bWFwX2xlZ2VuZF9wYXJhbSA9IGxpc3QobGVnZW5kX2RpcmVjdGlvbiA9ICdob3Jpem9udGFsJykpCgpybmFUcmVlSGVhdG1hcERyYXcgPC0gZ3JpZC5ncmFiRXhwcihkcmF3KHJuYVRyZWVIZWF0bWFwLGFubm90YXRpb25fbGVnZW5kX2xpc3QgPSBhbGxMZWdlbmRzLCBoZWF0bWFwX2xlZ2VuZF9zaWRlID0gJ2JvdHRvbScsIGFubm90YXRpb25fbGVnZW5kX3NpZGUgPSAncmlnaHQnLCBsZWdlbmRfZ3JvdXBpbmcgPSAnb3JpZ2luYWwnKSkgJT4lIGFzLmdncGxvdAoKZ2dzYXZlMihmaWxlbmFtZSA9ICdmaWd1cmU0Qi5wZGYnLHBsb3QgPSBybmFUcmVlSGVhdG1hcERyYXcsZGV2aWNlID0gJ3BkZicscGF0aCA9ICdjbHVzdGVyaW5nLycsZHBpID0gNjAwLHdpZHRoID0gMTAsaGVpZ2h0ID0gMTAsdW5pdHMgPSAnaW4nKQpgYGAKCiMjIyMgSm9pbnQgUGxvdHMKYGBge3J9Cmh0UHJvdFRyZWUgPC0gcHJvdFRyZWVIZWF0bWFwRHJhdwpodFJOQVRyZWUgPC0gZ3JpZC5ncmFiRXhwcihkcmF3KHJuYVRyZWVIZWF0bWFwLGhlYXRtYXBfbGVnZW5kX3NpZGUgPSAnYm90dG9tJykpICU+JSBhcy5nZ3Bsb3QKam9pbnRQbG90VHJlZSA8LSBwbG90X2dyaWQoaHRQcm90VHJlZSxodFJOQVRyZWUsbGFiZWxzID0gTEVUVEVSU1sxOjJdKQpnZ3NhdmUyKGZpbGVuYW1lID0gJ2ZpZ3VyZTQucGRmJyxwbG90ID0gam9pbnRQbG90VHJlZSxkZXZpY2UgPSAncGRmJyxwYXRoID0gJ2NsdXN0ZXJpbmcvJyxkcGkgPSA2MDAsd2lkdGggPSAxNSxoZWlnaHQgPSAxMCx1bml0cyA9ICdpbicpCmBgYAoKIyMgQ2x1c3RlcmluZyB3aXRoIGZlYXR1cmVzIGZyb20gR1NFQQoKIyMjIExpc3Qgb2YgZmVhdHVyZXMgZGlmZmVyZW50aWFsbHkgZXhwcmVzc2VkCgojIyMjIFByb3Rlb21pY3MKYGBge2Jhc2h9Cm1rZGlyIH4vU0NBTkJfUFVCTElDQVRJT04vY2x1c3RlcmluZy9wcm90ZW9taWNzX2dzZWFfY2x1c3RlcmluZwpta2RpciB+L1NDQU5CX1BVQkxJQ0FUSU9OL2NsdXN0ZXJpbmcvdHJhbnNjcmlwdG9taWNzX2dzZWFfY2x1c3RlcmluZwoKY3AgLVJ2IH4vU0NBTkJfUFVCTElDQVRJT04vREVfYW5hbHlzaXMvREVfZm9yX0dTRUEvKlByb3Rlb21pY3MqLyoudHN2IH4vU0NBTkJfUFVCTElDQVRJT04vY2x1c3RlcmluZy9wcm90ZW9taWNzX2dzZWFfY2x1c3RlcmluZwpjcCAtUnYgfi9TQ0FOQl9QVUJMSUNBVElPTi9ERV9hbmFseXNpcy9ERV9mb3JfR1NFQS8qUk5BKi8qLnRzdiB+L1NDQU5CX1BVQkxJQ0FUSU9OL2NsdXN0ZXJpbmcvdHJhbnNjcmlwdG9taWNzX2dzZWFfY2x1c3RlcmluZwpgYGAKCmBgYHtyfQpERUZpbGVzUHJvdCA8LSBsaXN0LmZpbGVzKHBhdHRlcm4gPSAnKi50c3YnLHBhdGggPSAnY2x1c3RlcmluZy9wcm90ZW9taWNzX2dzZWFfY2x1c3RlcmluZy8nKQpERURhdGFQcm90IDwtIG1hcCgueCA9IERFRmlsZXNQcm90LC5mID0gfnJlYWRfdHN2KGZpbGUgPSBwYXN0ZTAoJ2NsdXN0ZXJpbmcvcHJvdGVvbWljc19nc2VhX2NsdXN0ZXJpbmcvJywueCkpKQpuYW1lcyhERURhdGFQcm90KSA8LSBERUZpbGVzUHJvdAoKREVEYXRhRmlsdGVyUHJvdCA8LSBtYXAoLnggPSBERURhdGFQcm90LC5mID0gfmZpbHRlcigueCwueFtzdHJfZGV0ZWN0KHN0cmluZyA9IGNvbG5hbWVzKC54KSxwYXR0ZXJuID0gJ0FkalBWYWwnKV08MC4xKSkgJT4lIAogIG1hcCgueCA9IC4sLmYgPSB+ZmlsdGVyKC54LGFicygueFtzdHJfZGV0ZWN0KHN0cmluZyA9IGNvbG5hbWVzKC54KSxwYXR0ZXJuID0gJ2xvZzJGb2xkQ2hhbmdlJyldKT49MS41KSkgJT4lIAogIG1hcCgueCA9IC4sLmYgPSB+cHVsbCgueCxQcm90ZWluKSkgJT4lIAogIHVubGlzdCAlPiUgCiAgdW5pcXVlCmBgYAoKIyMjIyBUcmFuc2NyaXB0b21pY3MKYGBge3J9CkRFRmlsZXNSTkEgPC0gbGlzdC5maWxlcyhwYXR0ZXJuID0gJyoudHN2JyxwYXRoID0gJ2NsdXN0ZXJpbmcvdHJhbnNjcmlwdG9taWNzX2dzZWFfY2x1c3RlcmluZy8nKQpERURhdGFSTkEgPC0gbWFwKC54ID0gREVGaWxlc1JOQSwuZiA9IH5yZWFkX3RzdihmaWxlID0gcGFzdGUwKCdjbHVzdGVyaW5nL3RyYW5zY3JpcHRvbWljc19nc2VhX2NsdXN0ZXJpbmcvJywueCkpKQpuYW1lcyhERURhdGFSTkEpIDwtIERFRmlsZXNSTkEKCkRFRGF0YUZpbHRlclJOQSA8LSBtYXAoLnggPSBERURhdGFSTkEsLmYgPSB+ZmlsdGVyKC54LC54W3N0cl9kZXRlY3Qoc3RyaW5nID0gY29sbmFtZXMoLngpLHBhdHRlcm4gPSAnQWRqUFZhbCcpXTwwLjEpKSAlPiUgCiAgbWFwKC54ID0gLiwuZiA9IH5maWx0ZXIoLngsYWJzKC54W3N0cl9kZXRlY3Qoc3RyaW5nID0gY29sbmFtZXMoLngpLHBhdHRlcm4gPSAnbG9nMkZvbGRDaGFuZ2UnKV0pPj0xLjUpKSAlPiUgCiAgbWFwKC54ID0gLiwuZiA9IH5wdWxsKC54LEdlbmUpKSAlPiUgCiAgdW5saXN0ICU+JSAKICB1bmlxdWUKYGBgCgojIyMgRmlsdGVyIERhdGEKCiMjIyMgUHJvdGVvbWljcwpgYGB7cn0KcHJvdGVpbkRhdGEgPC0gJ0RJQS9EYXRhREUvRElBTk4vRElBX0RJQU5OX1Byb3RlaW5fUlJvbGx1cC9ESUFfRElBTk5fUlJvbGx1cF9HZW5lcy50c3YnICU+JSAKICByZWFkX3Rzdgpwcm90ZWluRGF0YUZpbHRlcmVkIDwtIHByb3RlaW5EYXRhICU+JSAKICBmaWx0ZXIoUHJvdGVpbiAlaW4lIERFRGF0YUZpbHRlclByb3QpICU+JSAKICBkcm9wX25hCmBgYAoKIyMjIyBUcmFuc2NyaXB0b21pY3MKYGBge3J9CnJuYURhdGEgPC0gJ1JOQS1zZXEvR1NFOTYwNThfZ2VuZV9leHByZXNzaW9uX1JlZHVjZWQudHN2JyAlPiUgCiAgcmVhZF90c3YKcm5hRGF0YUZpbHRlcmVkIDwtIHJuYURhdGEgJT4lIAogIGZpbHRlcihHZW5lICVpbiUgREVEYXRhRmlsdGVyUk5BKSAlPiUgCiAgZHJvcF9uYQpgYGAKCiMjIyBNYXRjaGluZyBEYXRhCgojIyMjIFByb3Rlb21pY3MKYGBge3J9CnByb3RDb21tb25TYW1wbGVzIDwtIHByb3RlaW5EYXRhRmlsdGVyZWQgJT4lIAogIGRwbHlyOjpzZWxlY3QoR2VuZXMsZGVzaWduJFBvc2l0aW9uKQpgYGAKCiMjIyMgVHJhbnNjcmlwdG9taWNzCmBgYHtyfQpybmFDb21tb25TYW1wbGVzIDwtIHJuYURhdGFGaWx0ZXJlZCAlPiUgCiAgZHBseXI6OnNlbGVjdChHZW5lLGRlc2lnbiRTYW1wbGVfdGl0bGUpCmBgYAoKIyMjIENsdXN0ZXJpbmcKCiMjIyMgRElBLU5OCmBgYHtyfQpwcm90ZWluRGF0YVJlZHVjZWQgPC0gcHJvdENvbW1vblNhbXBsZXMgJT4lIAogIGRwbHlyOjpzZWxlY3QoZGVzaWduJFBvc2l0aW9uKQoKIyBDYWxjdWxhdGlvbiBvZiBaLVNjb3JlcwpjYWxaU2NvcmUgPC0gZnVuY3Rpb24oeCkgKHgtbWVhbih4KSkvc2QoeCkKCnByb3RlaW5kYXRhTm9ybSA8LSB0KGFwcGx5KHByb3RlaW5EYXRhUmVkdWNlZCwxLGNhbFpTY29yZSkpCmNvbG5hbWVzKHByb3RlaW5kYXRhTm9ybSkgPC0gTlVMTAoKYW5ub3RhdGlvbkNvbHVtbiA8LSBIZWF0bWFwQW5ub3RhdGlvbihQQU01MD1hbm5vX3NpbXBsZSh4ID0gZGVzaWduJFBBTTUwLAogICAgICAgICAgICAgICAgICAgICAgICAgICAgICAgICAgICAgICAgICAgICAgICAgICAgICAgIGNvbCA9IGMoJ0FJTVNfUEFNNTAuQmFzYWwnPScjZTMxYTFjJywnQUlNU19QQU01MC5IZXIyJz0nI2ZiOWE5OScsJ0FJTVNfUEFNNTAuTHVtQSc9JyMxZjc4YjQnLCdBSU1TX1BBTTUwLkx1bUInPScjYTZjZWUzJywnQUlNU19QQU01MC5Ob3JtYWwnPScjMzNhMDJjJyksCiAgICAgICAgICAgICAgICAgICAgICAgICAgICAgICAgICAgICAgICAgICAgICAgICAgICAgICAgbmFfY29sID0gJyM4MDgwODAnLAogICAgICAgICAgICAgICAgICAgICAgICAgICAgICAgICAgICAgICAgICAgICAgICAgICAgICAgIGdwID0gZ3Bhcih0aXRsZT0nUEFNNTAnKSksCiAgICAgICAgICAgICAgICAgICAgICAgICAgICAgICAgICAgICAgTm9kZVN0YXR1cz1hbm5vX3NpbXBsZSh4ID0gZGVzaWduJGBMTiBzdGF0dXNgLAogICAgICAgICAgICAgICAgICAgICAgICAgICAgICAgICAgICAgICAgICAgICAgICAgICAgICAgICAgICAgY29sID0gYygnTm9kZVBvc2l0aXZlJz0nIzAwMDAwMCcsJ05vZGVOZWdhdGl2ZSc9JyNmZmZmZmYnKSwKICAgICAgICAgICAgICAgICAgICAgICAgICAgICAgICAgICAgICAgICAgICAgICAgICAgICAgICAgICAgIG5hX2NvbCA9ICcjODA4MDgwJywKICAgICAgICAgICAgICAgICAgICAgICAgICAgICAgICAgICAgICAgICAgICAgICAgICAgICAgICAgICAgIGdwID0gZ3Bhcih0aXRsZT0nTm9kZVN0YXR1cycpKSwKICAgICAgICAgICAgICAgICAgICAgICAgICAgICAgICAgICAgICBIRVIyPWFubm9fc2ltcGxlKHggPSBkZXNpZ24kYEhFUjIgc3RhdHVzYCwKICAgICAgICAgICAgICAgICAgICAgICAgICAgICAgICAgICAgICAgICAgICAgICAgICAgICAgIGNvbCA9IGMoJ1Bvcyc9JyMwMDAwMDAnLCdOZWcnPScjZmZmZmZmJyksCiAgICAgICAgICAgICAgICAgICAgICAgICAgICAgICAgICAgICAgICAgICAgICAgICAgICAgICBuYV9jb2wgPSAnIzgwODA4MCcsCiAgICAgICAgICAgICAgICAgICAgICAgICAgICAgICAgICAgICAgICAgICAgICAgICAgICAgICBncCA9IGdwYXIodGl0bGU9J0hFUjInKSksCiAgICAgICAgICAgICAgICAgICAgICAgICAgICAgICAgICAgICAgRVI9YW5ub19zaW1wbGUoeCA9IGRlc2lnbiRgRVIgc3RhdHVzYCwKICAgICAgICAgICAgICAgICAgICAgICAgICAgICAgICAgICAgICAgICAgICAgICAgICAgICBjb2wgPSBjKCdQb3MnPScjMDAwMDAwJywnTmVnJz0nI2ZmZmZmZicpLAogICAgICAgICAgICAgICAgICAgICAgICAgICAgICAgICAgICAgICAgICAgICAgICAgICAgIG5hX2NvbCA9ICcjODA4MDgwJywKICAgICAgICAgICAgICAgICAgICAgICAgICAgICAgICAgICAgICAgICAgICAgICAgICAgICBncCA9IGdwYXIodGl0bGU9J0VSJykpLAogICAgICAgICAgICAgICAgICAgICAgICAgICAgICAgICAgICAgIFBSPWFubm9fc2ltcGxlKHggPSBkZXNpZ24kYFBSIHN0YXR1c2AsCiAgICAgICAgICAgICAgICAgICAgICAgICAgICAgICAgICAgICAgICAgICAgICAgICAgICAgIGNvbCA9IGMoJ1Bvcyc9JyMwMDAwMDAnLCdOZWcnPScjZmZmZmZmJyksCiAgICAgICAgICAgICAgICAgICAgICAgICAgICAgICAgICAgICAgICAgICAgICAgICAgICAgIG5hX2NvbCA9ICcjODA4MDgwJywKICAgICAgICAgICAgICAgICAgICAgICAgICAgICAgICAgICAgICAgICAgICAgICAgICAgICAgZ3AgPSBncGFyKHRpdGxlPSdQUicpKSkKCmxlZ2VuZFBBTTUwIDwtIExlZ2VuZChsYWJlbHMgPSBjKCdCYXNhbCcsJ0hlcjInLCdMdW1BJywnTHVtQicsJ05vcm1hbCcpLAogICAgICAgICAgICAgICAgICAgICAgdGl0bGUgPSAnUEFNNTAgc3VidHlwZScsCiAgICAgICAgICAgICAgICAgICAgICBib3JkZXIgPSAnYmxhY2snLAogICAgICAgICAgICAgICAgICAgICAgbGVnZW5kX2dwID0gZ3BhcihmaWxsPWMoJ0Jhc2FsJz0nI2UzMWExYycsJ0hlcjInPScjZmI5YTk5JywnTHVtQSc9JyMxZjc4YjQnLCdMdW1CJz0nI2E2Y2VlMycsJ05vcm1hbCc9JyMzM2EwMmMnKSkpCmxlZ2VuZEhlcjIgPC0gTGVnZW5kKGxhYmVscyA9IGMoJ1Bvc2l0aXZlJywnTmVnYXRpdmUnLCdOQScpLAogICAgICAgICAgICAgICAgICAgICB0aXRsZSA9ICdIRVIyIHN0YXR1cycsCiAgICAgICAgICAgICAgICAgICAgIGJvcmRlciA9ICdibGFjaycsCiAgICAgICAgICAgICAgICAgICAgIGxlZ2VuZF9ncCA9IGdwYXIoZmlsbD1jKCdQb3NpdGl2ZSc9JyMwMDAwMDAnLCdOZWdhdGl2ZSc9JyNmZmZmZmYnLCdOQSc9JyM4MDgwODAnKSkpCmxlZ2VuZE5vZGVTdGF0dXMgPC0gTGVnZW5kKGxhYmVscyA9IGMoJ1Bvc2l0aXZlJywnTmVnYXRpdmUnKSwKICAgICAgICAgICAgICAgICAgICAgICAgICAgdGl0bGUgPSAnTm9kZSBzdGF0dXMnLAogICAgICAgICAgICAgICAgICAgICAgICAgICBib3JkZXIgPSAnYmxhY2snLAogICAgICAgICAgICAgICAgICAgICAgICAgICBsZWdlbmRfZ3AgPSBncGFyKGZpbGw9YygnUG9zaXRpdmUnPScjMDAwMDAwJywnTmVnYXRpdmUnPScjZmZmZmZmJykpKQpsZWdlbmRFUiA8LSBMZWdlbmQobGFiZWxzID0gYygnUG9zaXRpdmUnLCdOZWdhdGl2ZScpLAogICAgICAgICAgICAgICAgICAgdGl0bGUgPSAnRVIgc3RhdHVzJywKICAgICAgICAgICAgICAgICAgIGJvcmRlciA9ICdibGFjaycsCiAgICAgICAgICAgICAgICAgICBsZWdlbmRfZ3AgPSBncGFyKGZpbGw9YygnUG9zaXRpdmUnPScjMDAwMDAwJywnTmVnYXRpdmUnPScjZmZmZmZmJykpKQpsZWdlbmRQUiA8LSBMZWdlbmQobGFiZWxzID0gYygnUG9zaXRpdmUnLCdOZWdhdGl2ZScpLAogICAgICAgICAgICAgICAgICAgIHRpdGxlID0gJ1BSIHN0YXR1cycsCiAgICAgICAgICAgICAgICAgICAgYm9yZGVyID0gJ2JsYWNrJywKICAgICAgICAgICAgICAgICAgICBsZWdlbmRfZ3AgPSBncGFyKGZpbGw9YygnUG9zaXRpdmUnPScjMDAwMDAwJywnTmVnYXRpdmUnPScjZmZmZmZmJykpKQoKYWxsTGVnZW5kcyA8LSBwYWNrTGVnZW5kKGxlZ2VuZFBBTTUwLGxlZ2VuZEhlcjIsbGVnZW5kTm9kZVN0YXR1cyxsZWdlbmRFUixsZWdlbmRQUikKCnByb3RlaW5IZWF0bWFwR1NFQSA8LSBIZWF0bWFwKG1hdHJpeCA9IHByb3RlaW5kYXRhTm9ybSx0b3BfYW5ub3RhdGlvbiA9IGFubm90YXRpb25Db2x1bW4sIG5hbWUgPSAnUHJvdGVpbiBpbnRlbnNpdHknLCBoZWF0bWFwX2xlZ2VuZF9wYXJhbSA9IGxpc3QobGVnZW5kX2RpcmVjdGlvbiA9ICdob3Jpem9udGFsJykpCgpwcm90ZWluSGVhdG1hcEdTRUFEcmF3IDwtIGdyaWQuZ3JhYkV4cHIoZHJhdyhwcm90ZWluSGVhdG1hcEdTRUEsYW5ub3RhdGlvbl9sZWdlbmRfbGlzdCA9IGFsbExlZ2VuZHMsIGhlYXRtYXBfbGVnZW5kX3NpZGUgPSAnYm90dG9tJywgYW5ub3RhdGlvbl9sZWdlbmRfc2lkZSA9ICdyaWdodCcsIGxlZ2VuZF9ncm91cGluZyA9ICdvcmlnaW5hbCcpKSAlPiUgYXMuZ2dwbG90CgpnZ3NhdmUyKGZpbGVuYW1lID0gJ3N1cHBsZW1lbnRhcnlfZmlndXJlUzJBLnBkZicscGxvdCA9IHByb3RlaW5IZWF0bWFwR1NFQURyYXcsZGV2aWNlID0gJ3BkZicscGF0aCA9ICdjbHVzdGVyaW5nLycsZHBpID0gNjAwLHdpZHRoID0gMTAsaGVpZ2h0ID0gMTAsdW5pdHMgPSAnaW4nKQpgYGAKCiMjIyMgUk5BLXNlcQpgYGB7cn0Kcm5hUmVkdWNlZCA8LSBybmFDb21tb25TYW1wbGVzICU+JSAKICBkcGx5cjo6c2VsZWN0KGRlc2lnbiRTYW1wbGVfdGl0bGUpCgpybmFOb3JtIDwtIHQoYXBwbHkocm5hUmVkdWNlZCwxLGNhbFpTY29yZSkpCmNvbG5hbWVzKHJuYU5vcm0pIDwtIE5VTEwKCnJuYUhlYXRtYXBHU0VBIDwtIEhlYXRtYXAobWF0cml4ID0gcm5hTm9ybSx0b3BfYW5ub3RhdGlvbiA9IGFubm90YXRpb25Db2x1bW4sbmFtZSA9ICdHZW5lIGV4cHJlc3Npb24nLCBoZWF0bWFwX2xlZ2VuZF9wYXJhbSA9IGxpc3QobGVnZW5kX2RpcmVjdGlvbiA9ICdob3Jpem9udGFsJykpCgpybmFIZWF0bWFwR1NFQURyYXcgPC0gZ3JpZC5ncmFiRXhwcihkcmF3KHJuYUhlYXRtYXBHU0VBLGFubm90YXRpb25fbGVnZW5kX2xpc3QgPSBhbGxMZWdlbmRzLCBoZWF0bWFwX2xlZ2VuZF9zaWRlID0gJ2JvdHRvbScsIGFubm90YXRpb25fbGVnZW5kX3NpZGUgPSAncmlnaHQnLCBsZWdlbmRfZ3JvdXBpbmcgPSAnb3JpZ2luYWwnKSkgJT4lIGFzLmdncGxvdAoKZ2dzYXZlMihmaWxlbmFtZSA9ICdzdXBwbGVtZW50YXJ5X2ZpZ3VyZVMyQi5wZGYnLHBsb3QgPSBybmFIZWF0bWFwR1NFQURyYXcsZGV2aWNlID0gJ3BkZicscGF0aCA9ICdjbHVzdGVyaW5nLycsZHBpID0gNjAwLHdpZHRoID0gMTAsaGVpZ2h0ID0gMTAsdW5pdHMgPSAnaW4nKQpgYGAKCiMjIyMgSm9pbnQgUGxvdHMKYGBge3J9Cmh0UHJvdEdTRUEgPC0gcHJvdGVpbkhlYXRtYXBHU0VBRHJhdwpodFJOQUdTRUEgPC0gZ3JpZC5ncmFiRXhwcihkcmF3KHJuYUhlYXRtYXBHU0VBLGhlYXRtYXBfbGVnZW5kX3NpZGUgPSAnYm90dG9tJykpICU+JSBhcy5nZ3Bsb3QKam9pbnRQbG90R1NFQSA8LSBwbG90X2dyaWQoaHRQcm90R1NFQSxodFJOQUdTRUEsbGFiZWxzID0gTEVUVEVSU1sxOjJdKQpnZ3NhdmUyKGZpbGVuYW1lID0gJ3N1cHBsZW1lbnRhcnlfZmlndXJlUzIucGRmJyxwbG90ID0gam9pbnRQbG90R1NFQSxkZXZpY2UgPSAncGRmJyxwYXRoID0gJ2NsdXN0ZXJpbmcvJyxkcGkgPSA2MDAsd2lkdGggPSAxNSxoZWlnaHQgPSAxMCx1bml0cyA9ICdpbicpCmBgYAoK
