# Supplementary figures and images for: Choice of High-Throughput Proteomics Method Affects Data Integration with Transcriptomics and the Potential Use in Biomarker Discovery
Source: Cancers (Basel). 2022 Nov 23;14(23):5761. doi: 10.3390/cancers14235761 (PMC9736226; doi:10.3390/cancers14235761)

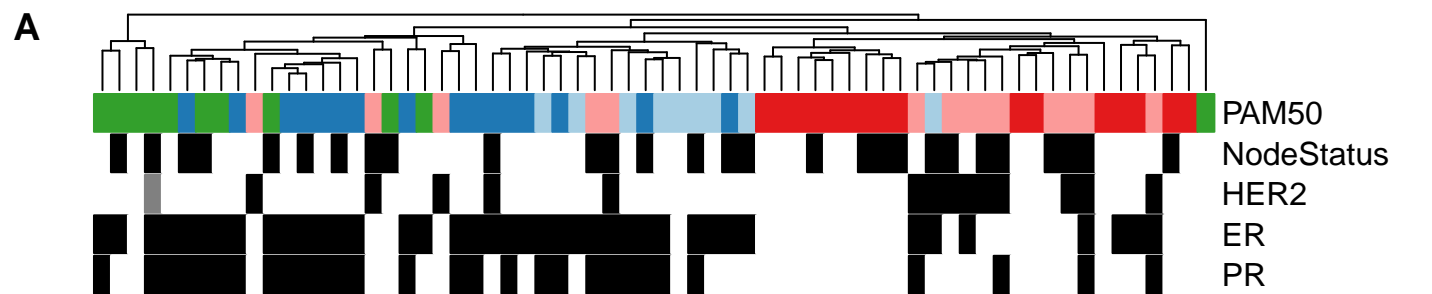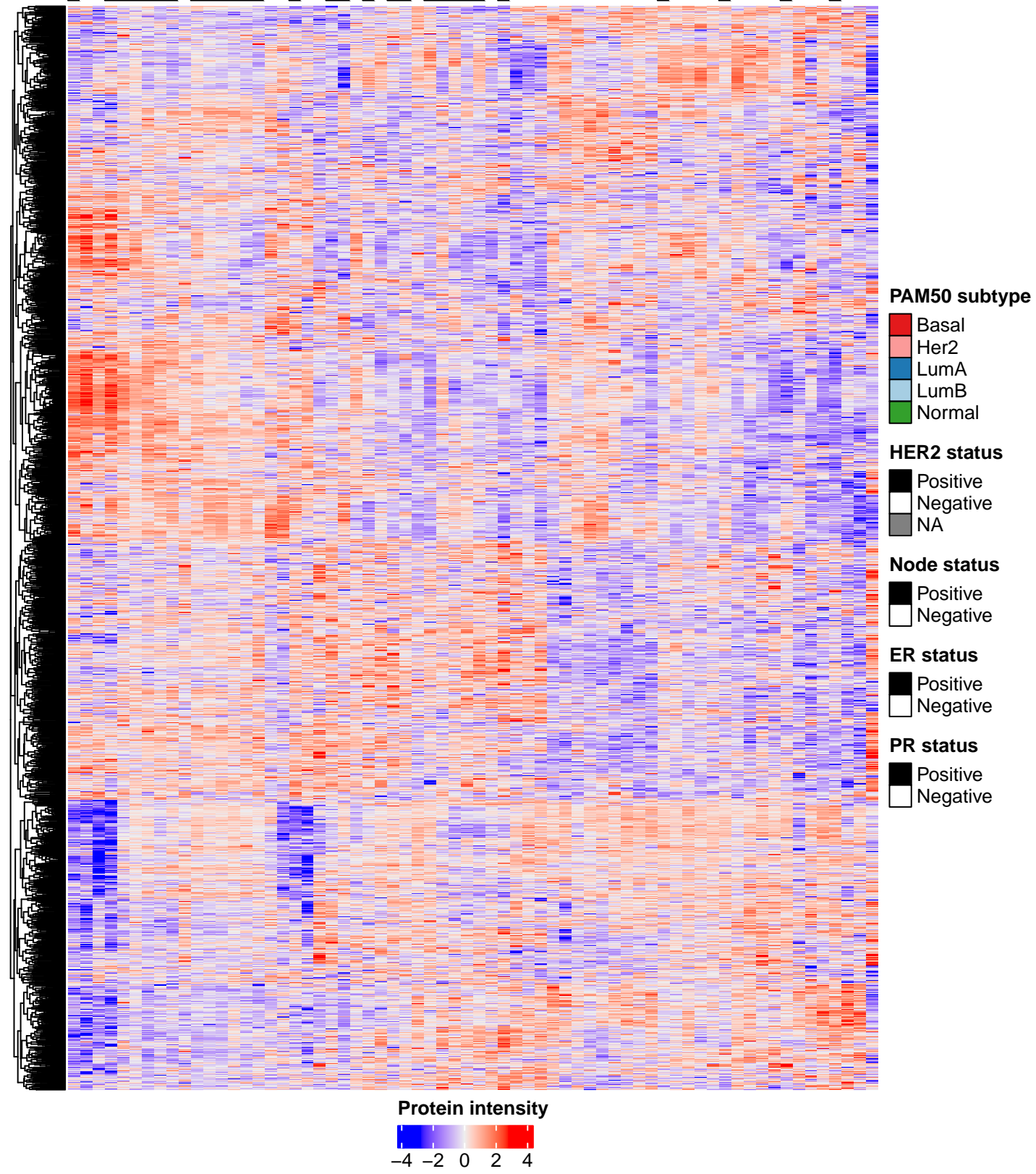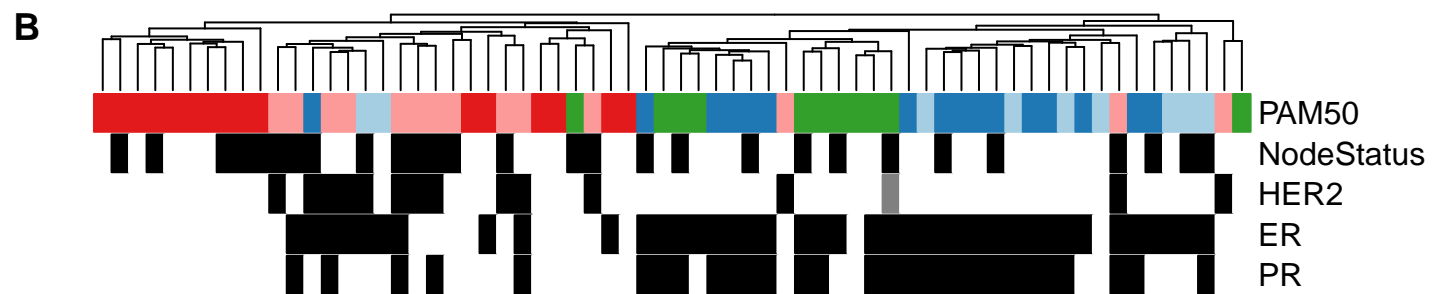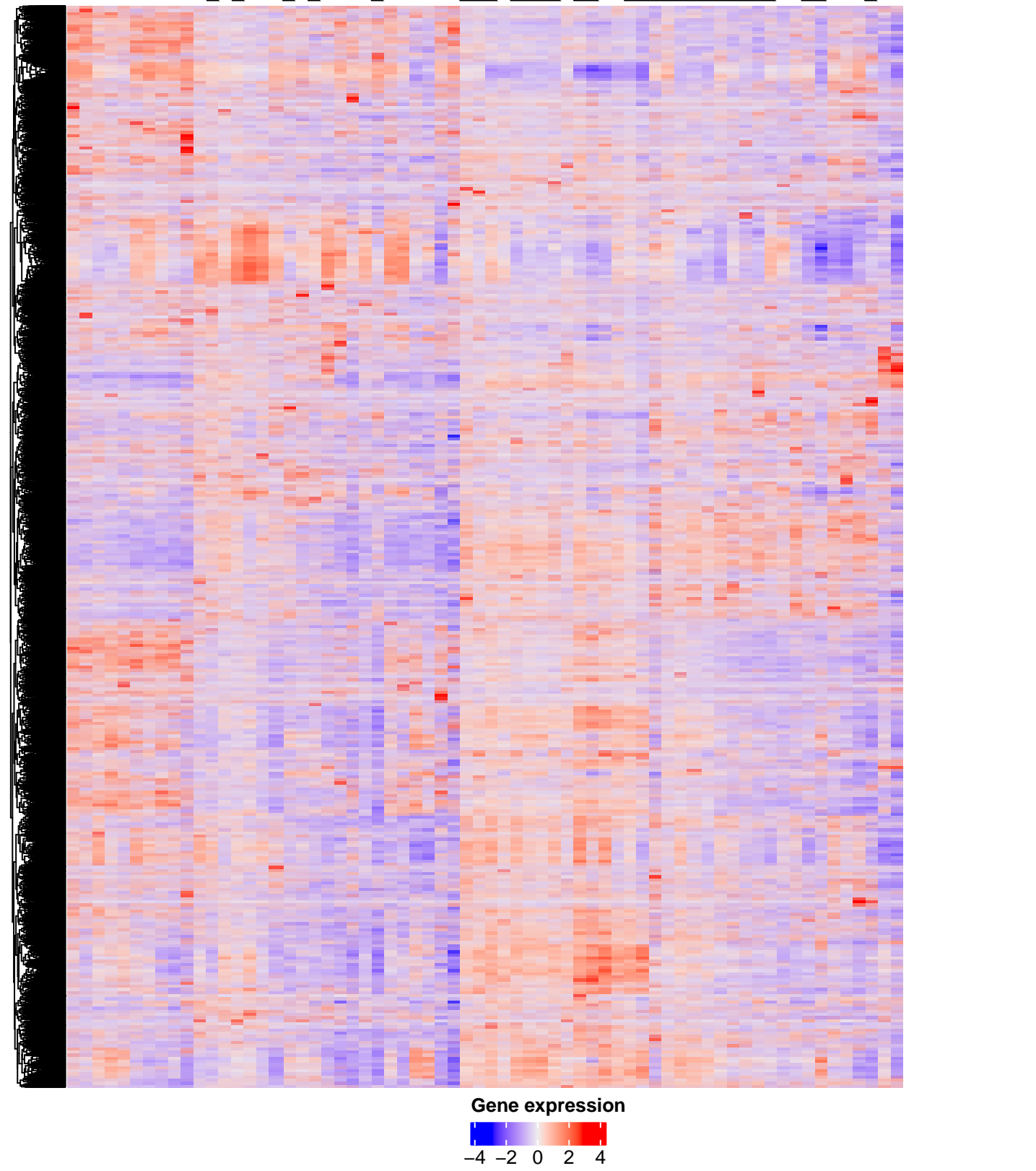

Supplement: Supplementary file 1 [file cancers-14-05761-s001.zip › supplementary_files/supplementary_figureS1.pdf]

A

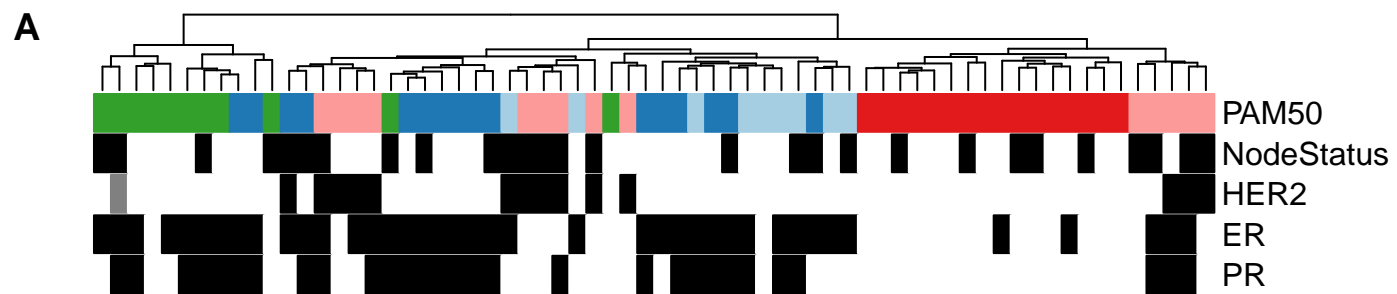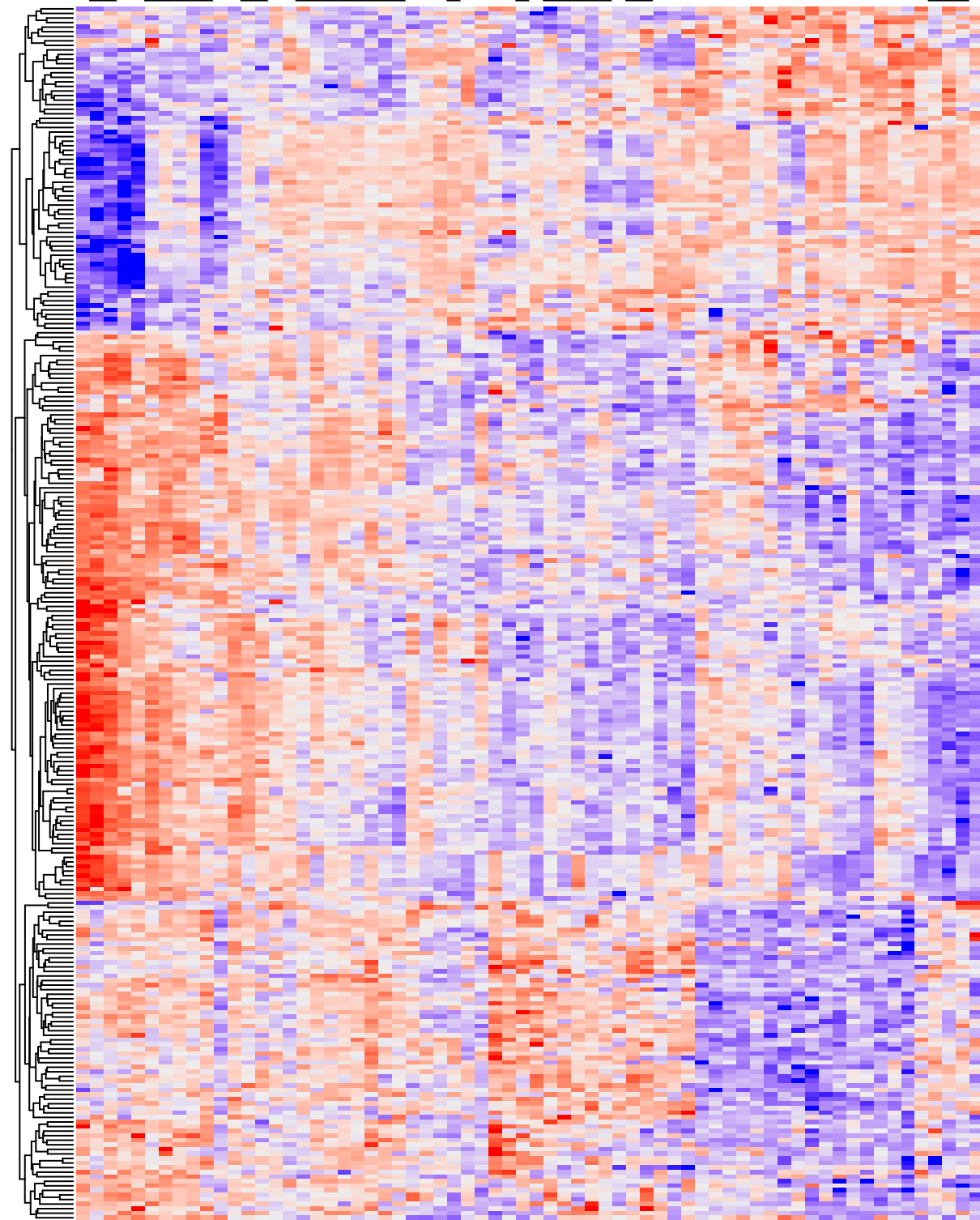

Protein intensity

-4 -2 0 2 4

B

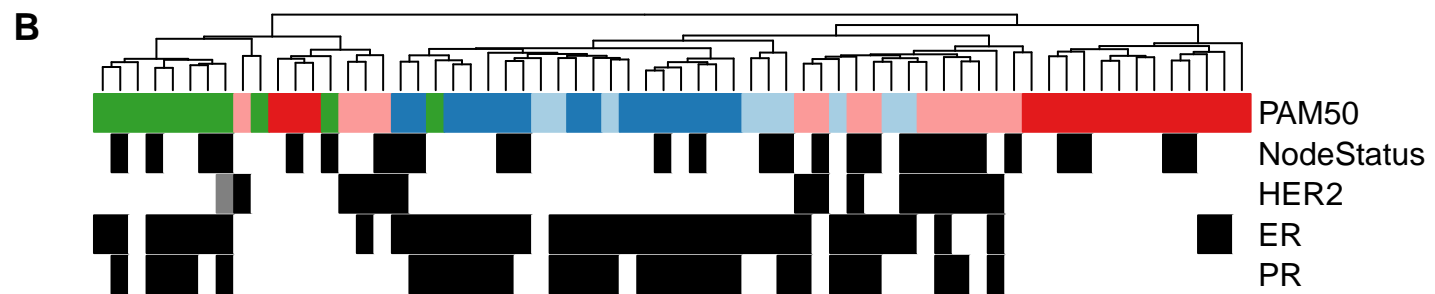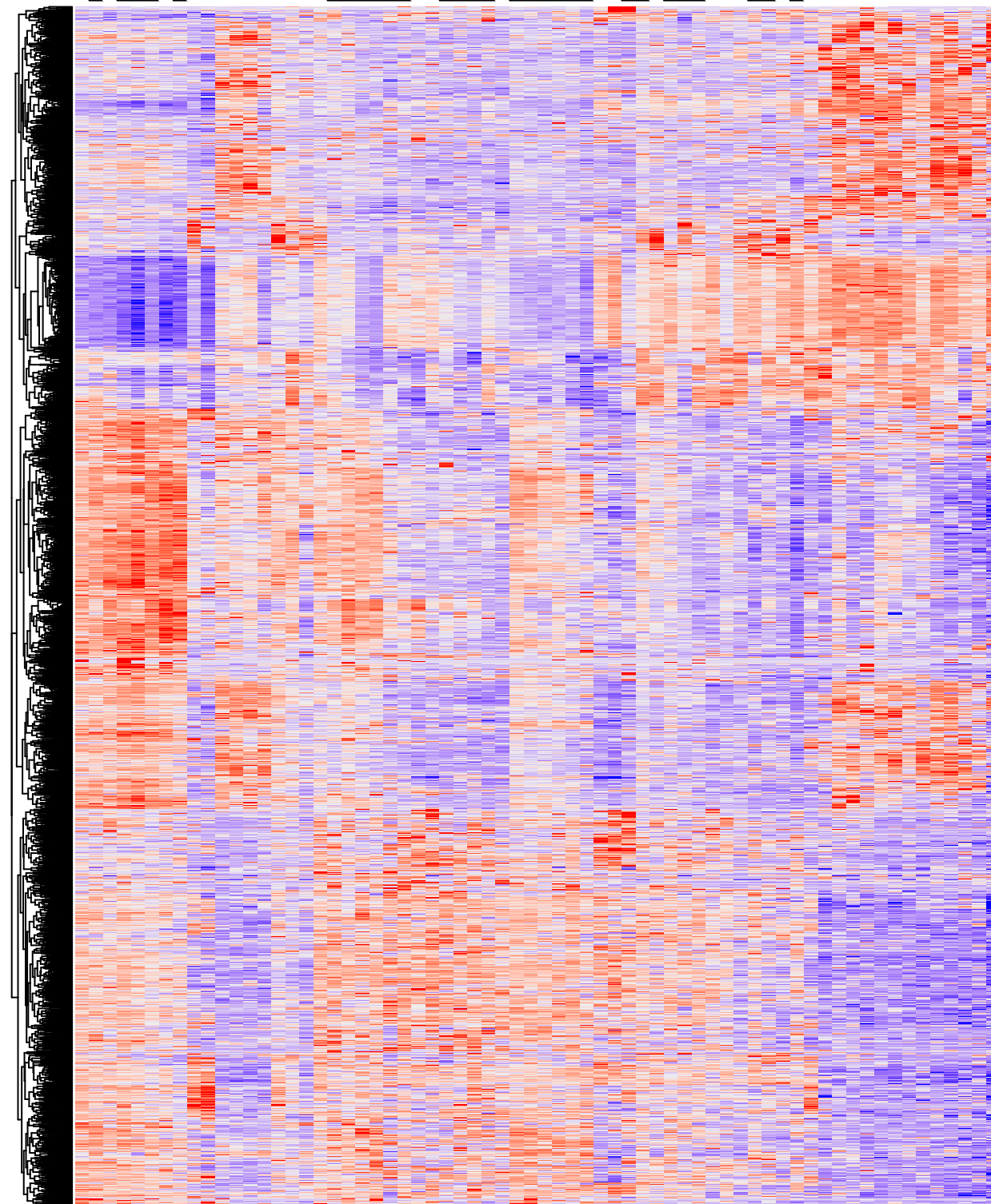

Gene expression

-4 -2 0 2 4

Supplement: Supplementary file 1 [file cancers-14-05761-s001.zip › supplementary_files/supplementary_figureS2.pdf]
